# Rapid genetic adaptation to recently colonized environments is driven by genes underlying life history traits

**DOI:** 10.1186/s12864-021-07553-x

**Published:** 2021-04-14

**Authors:** Xiaoshen Yin, Alexander S. Martinez, Maria S. Sepúlveda, Mark R. Christie

**Affiliations:** 1 Department of Biological Sciences, Purdue University, 915 W. State St., West Lafayette, Indiana, 47907-2054 USA; 2 Department of Forestry and Natural Resources, Purdue University, 715 W. State St., West Lafayette, Indiana, 47907-2054 USA

**Keywords:** Founder effects, Genetic bottleneck, Invasive species, Selection, Rapid genetic adaptation, RNA-seq, Sea lamprey *Petromyzon marinus*

## Abstract

**Background:**

Uncovering the mechanisms underlying rapid genetic adaptation can provide insight into adaptive evolution and shed light on conservation, invasive species control, and natural resource management. However, it can be difficult to experimentally explore rapid adaptation due to the challenges associated with propagating and maintaining species in captive environments for long periods of time. By contrast, many introduced species have experienced strong selection when colonizing environments that differ substantially from their native range and thus provide a “natural experiment” for studying rapid genetic adaptation. One such example occurred when sea lamprey (*Petromyzon marinus*), native to the northern Atlantic, naturally migrated into Lake Champlain and expanded their range into the Great Lakes via man-made shipping canals.

**Results:**

Utilizing 368,886 genome-wide single nucleotide polymorphisms (SNPs), we calculated genome-wide levels of genetic diversity (i.e.*,* heterozygosity and *π*) for sea lamprey collected from native (Connecticut River), native but recently colonized (Lake Champlain), and invasive (Lake Michigan) populations, assessed genetic differentiation between all populations, and identified candidate genes that responded to selection imposed by the novel environments. We observed a 14 and 24% reduction in genetic diversity in Lake Michigan and Lake Champlain populations, respectively, compared to individuals from the Connecticut River, suggesting that sea lamprey populations underwent a genetic bottleneck during colonization. Additionally, we identified 121 and 43 outlier genes in comparisons between Lake Michigan and Connecticut River and between Lake Champlain and Connecticut River, respectively. Six outlier genes that contained synonymous SNPs in their coding regions and two genes that contained nonsynonymous SNPs may underlie the rapid evolution of growth (i.e.*, GHR*), reproduction (i.e.*, PGR*, *TTC25*, *STARD10*), and bioenergetics (i.e.*, OXCT1*, *PYGL*, *DIN4*, *SLC25A15*).

**Conclusions:**

By identifying the genomic basis of rapid adaptation to novel environments, we demonstrate that populations of invasive species can be a useful study system for understanding adaptive evolution. Furthermore, the reduction in genome-wide levels of genetic diversity associated with colonization coupled with the identification of outlier genes underlying key life history traits known to have changed in invasive sea lamprey populations (e.g.*,* growth, reproduction) illustrate the utility in applying genomic approaches for the successful management of introduced species.

**Supplementary Information:**

The online version contains supplementary material available at 10.1186/s12864-021-07553-x.

## Background

Invasive species can rapidly establish and spread in new environments that often have substantially different abiotic and biotic conditions than found throughout their native range [1, 2]. This widely observed phenomenon has triggered investigations into how invasive species are able to quickly adapt to such different conditions [1]. Identifying the genes that drive rapid genetic adaptation can provide a better framework for understanding how and when rapid adaptation is likely to occur, shedding light on conservation, invasive species control, and natural resource management [3, 4]. For many species, experimental manipulations investigating rapid genetic adaptation can be difficult due to long generation times and the challenges associated with propagating species in captive environments. Introduced species, by contrast, can sometimes provide a natural “experiment” for identifying the genetic basis underlying the rapid genetic adaptation associated with the colonization of novel environments [5, 6]. One such example occurred when sea lamprey (*Petromyzon marinus*), a parasitic, jawless vertebrate native to the northern Atlantic, invaded the Laurentian Great Lakes.

In their native range, which includes most of the northern Atlantic and surrounding regions [7], sea lamprey are an anadromous, semelparous species with a bipartite life cycle consisting of distinct larval and adult stages. During their larval stages, sea lamprey burrow into soft and sandy substrates in freshwater streams and filter feed for an average of four to eight years (reported range is 2–19 years) [8]. After undergoing a series of behavioral and physiological modifications essential for a hematophagous, parasitic lifestyle, larval sea lamprey (i.e.*,* ammocoetes) transform into parasitic juveniles [9] and migrate out to the ocean. Once in the ocean, sea lamprey parasitize many host species such as herring (*Clupea harengus*), mackerel (*Scomber scombrus*), and Atlantic salmon (*Salmo salar*) [10]. Juvenile sea lamprey attach to their hosts using sharp teeth and are able to continuously feed on the blood and tissue of their hosts by secreting anticoagulants [11]. The parasitic feeding stage in sea lamprey lasts between 20 to 36 months, after which sea lamprey return to rivers and streams for spawning. Instead of returning to their natal streams, like many other anadromous fishes (e.g.*,* salmon), sea lamprey rely on a pheromone produced by larvae in streams and rivers as a cue for migration [12–15]. Thus, the choice of which stream to spawn in is largely driven by larval abundance, where high larval abundance results in more pheromone released and can generate a strong signal to attract adult sea lamprey for spawning. This process results in few lamprey returning to spawn in the populations where they were born and the subsequent high gene flow among populations means that sea lamprey populations are largely panmictic [7, 12, 16].

Sea lamprey are native to the northern Atlantic coast [17], and migrated into Lake Ontario and Lake Champlain, where they were first observed in 1835 and 1841, respectively [18–20], through natural migrations via the St. Lawrence River [7]. The construction and improvement of shipping canals in the early 1800s allowed sea lamprey to expand their ranges to Lake Erie and then colonize Lakes Huron, Michigan, and Superior [7, 21, 22]. The first documented observations of sea lamprey for Lake Erie was in 1921 [22], Lake Michigan in 1936 [21], Lake Huron in 1937 [20], and Lake Superior in 1946 [20]. These colonization events were followed by substantial declines in native, commercially and ecologically important fishes such as lake trout (*Salvelinus namaycush*), burbot (*Lota lota*), lake whitefish (*Coregonus clupeaformis*), and walleye (*Sander vitreus*). In order to rehabilitate local fisheries, 3-trifluoromethyl-4-nitrophenol (TFM), a pesticide that targets the larval stage of sea lamprey, has been applied in Lake Michigan since 1960 and in Lake Champlain since 1990 [23, 24].

The ecology and environmental conditions found in the Great Lakes are substantially different from those found throughout the sea lamprey’s native range [17], and have changed some of sea lamprey’s life history characteristics. For example, invasive sea lamprey spend their parasitic life history stage entirely in the freshwater environment of the Great Lakes instead of migrating to the ocean. Thus, invasive sea lamprey never acclimate to high-salinity ocean environments. Sea lamprey in the Great Lakes also exhibit a faster growth rate, a shorter larval stage, a smaller adult body size, and a lower fecundity in comparison to individuals from native populations [25, 26]. Lastly, invasive sea lamprey spend more time feeding parasitically on a single host individual, and greater numbers of individual sea lamprey are often found attached to a single adult fish in the Great Lakes than in the Atlantic Ocean.

**Fig. 1:**
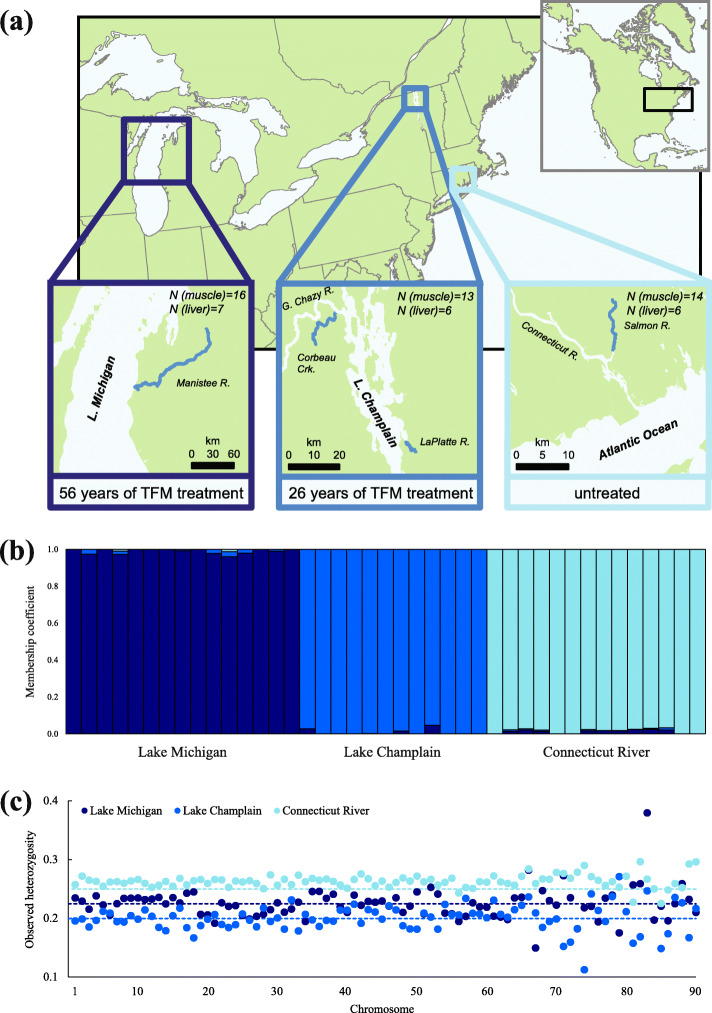
Map of sampling sites, population structure, and genetic diversity (observed heterozygosity). Larval sea lamprey were collected from Lake Michigan, Lake Champlain, and the Connecticut River, where the numbers reflect the number of muscle and liver samples of larval sea lamprey that were sequenced and used in this study (**a**). Larval sea lamprey collected from the three locations cluster clearly into three distinct groups (**b**). Mean observed heterozygosity across the genome is indicated by dashed lines and observed heterozygosity of chromosomes is indicated by points. Genetic diversity is the highest in Connecticut River, followed by Lake Michigan and Lake Champlain (**c**). Sea lamprey have 99 chromosomes, among which the first 90 are assembled. The map in (**a**) is modified with permission from Yin et al. [30]

While these differences in life history characteristics of invasive sea lamprey are suggestive of genetic adaptation, many of these traits may simply be a plastic response to different environments [27–29]. Determining which genes have responded to selection in the novel, recently colonized environment is valuable for understanding rapid genetic adaptation (i.e.*,* occurring in fewer than 200 years in invasive sea lamprey) to new environments in both sea lamprey and other species. To achieve this objective, we sampled larval sea lamprey from three locations: (1) the Connecticut River, a native-range tributary of the Atlantic Ocean, (2) Lake Michigan, where sea lamprey are invasive, and (3) Lake Champlain, an additional freshwater environment that sea lamprey colonized through natural migrations (Fig. 1a). Although sea lamprey may be native to Lake Champlain [7], they still needed to adjust to a novel, entirely-freshwater environment with vastly different ecological conditions. We used genome-wide single nucleotide polymorphisms (SNPs) identified via RNA-seq to calculate genome-wide levels of genetic diversity, assess genetic differentiation among populations, and identify genes that may have responded to selection imposed by the novel environments. By conducting this set of analyses, we aim to answer two primary questions: (1) Did sea lamprey populations colonizing Lake Michigan and Lake Champlain undergo a genetic bottleneck during colonization (i.e.*,* founder effects), and (2) what genes have responded to selection in the novel environments and how do they relate to the documented phenotypic shifts in life history traits?

## Results

### Population structure and observed heterozygosity

We collected 565 larval sea lamprey from the Manistee River in Lake Michigan, 517 larval sea lamprey from Corbeau Creek and the LaPlatte River in Lake Champlain, and 404 larval sea lamprey from the Connecticut River in 2016 (Fig. 1a). After a four-month acclimation period at the Purdue Aquaculture Research Laboratory, we sampled muscle tissues from a total of 43 individuals for RNA-seq (Lake Michigan: *n* = 16, Lake Champlain: *n* = 13, Connecticut River: *n* = 14). We also sampled liver tissue from a subset of the same individuals (42%), but those data were only used for validating our outlier loci (see Methods for details). After sequencing, alignment, and SNP calling and filtering, we obtained 368,886 SNPs, among which 359,025, 358,708 and 358,268 were in Hardy-Weinberg equilibrium in Lake Michigan, Lake Champlain, and Connecticut River populations, respectively. Using the model-based clustering methods implemented in STRUCTURE (version 2.3) [31–34], all sea lamprey were assigned to their collection locations (population membership coefficients ranged from 0.961 to 1 for Lake Michigan population, 0.954 to 1 for Lake Champlain population, and 0.969 to 1 for Connecticut River population) with *K* set to 3 (Fig. 1b). If we assumed all 41 samples originated from two populations (i.e.*, K* = 2), sea lamprey collected from Lake Michigan and Connecticut River clustered as one population while those from Lake Champlain were assigned to the other (Fig. S1). If we assumed these 41 samples were from four or more (i.e.*, K* = 4 or *K* = 10) populations, Lake Michigan and Lake Champlain individuals were still correctly assigned to their sampling locations, although there may be some subtle population structure within Connecticut River (Fig. S1). Here, we determined that *K* = 4 had the highest likelihood value but by only a small margin (Fig. S1). We presented *K* = 3 in the main text because the difference in likelihood values between *K* = 3 and *K* = 4 was small (−11,052,842 vs. −11,023,345) and the additional cluster only illustrates a small number of Connecticut River individuals with mixed ancestry.

Mean observed heterozygosity (*Ho*) across the genome was 0.261 for Connecticut River, 0.225 for Lake Michigan, and 0.200 for Lake Champlain. Using a randomization test (see Methods for details on the randomization test), mean heterozygosity across each chromosome was significantly different among three populations, with the highest genetic diversity in the Connecticut River population and the lowest genetic diversity in Lake Champlain (Fig. 1c, Fig. S2; *p*-value < 0.0001 for all three pairwise comparisons). These differences represent a 14% reduction in genome-wide levels of genetic diversity for Lake Michigan and a 24% reduction for Lake Champlain compared to the native range, Connecticut River. Mean observed heterozygosity and mean nucleotide diversity (*π*) for each chromosome were highly correlated (Fig. S3).

### Genetic differentiation and the identification of outlier genes

**Fig. 2:**
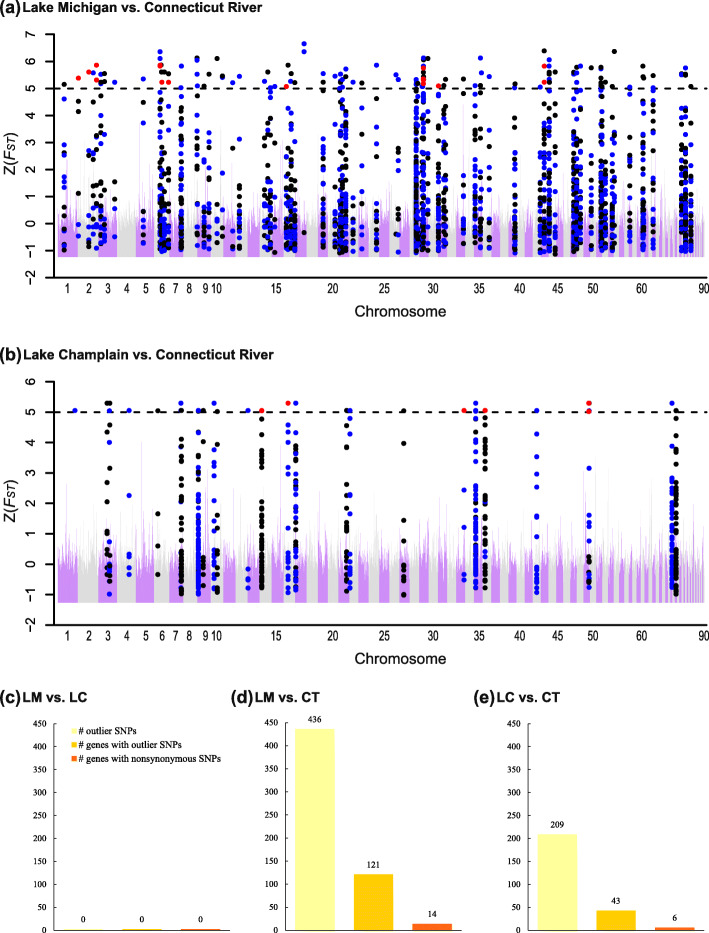
Genetic differentiation (*F*_*ST*_) and outlier genes for the three pairwise comparisons. *F*_*ST*_ is plotted across the 90 assembled chromosomes for the comparison between Lake Michigan and Connecticut River (**a**) and the comparison between Lake Champlain and Connecticut River (**b**). All SNPs on neighboring outlier genes are indicated by points in alternating colors, blue and black, with SNPs causing nonsynonymous mutations in red. *F*_*ST*_ averaged across SNPs in 100 Kb windows is plotted for chromosomes 1–90 in alternating colors, purple and grey. The dashed line indicates a threshold Z(*F*_*ST*_) of 5 for identifying outlier SNPs. While no outlier SNPs were detected in the comparison between Lake Michigan and Lake Champlain (c; LM vs. LC), 436 outlier SNPs, corresponding to 121 outlier genes, among which 14 contain outlier SNPs causing nonsynonymous substitutions, and 209 outlier SNPs, corresponding to 43 outlier genes, among which 6 contain outlier SNPs causing nonsynonymous substitutions, were detected between Lake Michigan and Connecticut River (d; LM vs. CT) and between Lake Champlain and Connecticut River (e; LC vs. CT)

In total, 290,739, 342,311 and 336,596 loci were kept for pairwise analyses because they were shared in comparisons between Lake Michigan and Lake Champlain, Lake Michigan and Connecticut River, and Lake Champlain and Connecticut River, respectively. Mean *F*_*ST*_ among all three populations equaled 0.136. For pairwise comparisons, *F*_*ST*_ between Lake Michigan and Connecticut River was 0.098, *F*_*ST*_ between Lake Champlain and Connecticut River was 0.123, and *F*_*ST*_ between Lake Michigan and Lake Champlain was 0.150. We detected 436 outlier SNPs (see Methods for details on identifying outlier SNPs) in 121 genes when comparing Lake Michigan and Connecticut River populations and 209 outlier SNPs in 43 genes when comparing Lake Champlain and Connecticut River populations (Fig. 2, Fig. S4, Table S2). We did not find a single outlier SNP (and consequently outlier gene) when comparing the Lake Michigan and Lake Champlain populations (Fig. 2c, Fig. S4) despite nearly identical sample sizes, read depth, and numbers of SNPs for all pairwise comparisons (Fig. S5). In the comparison between Lake Michigan and Connecticut River, 14 out of 121 outlier genes contained outlier SNPs causing nonsynonymous changes (Fig. 2d, Table S2). Similarly, six out of 43 genes contained nonsynonymous outlier SNPs in the comparison between Lake Champlain and Connecticut River (Fig. 2e, Table S2). Two genes, *CLTC* (clathrin heavy chain) and *RALA* (RAS like proto-oncogene A), were found to be in common in both comparisons involving the native, anadromous population (i.e.*,* Lake Michigan vs. Connecticut River and Lake Champlain vs. Connecticut River) (Table S2; see Table S3 for all gene names and identities). The identification of outlier SNPs and corresponding genes was supported by *F*_*ST*_, allele frequency difference (*AFD*), and genome scans based on *k*-nearest neighbor (kNN) techniques (Fig. S6, Table S2; see Methods below and Results in Supplementary Information for details).

### Gene ontology (GO) hierarchy networks of outlier genes

**Fig. 3:**
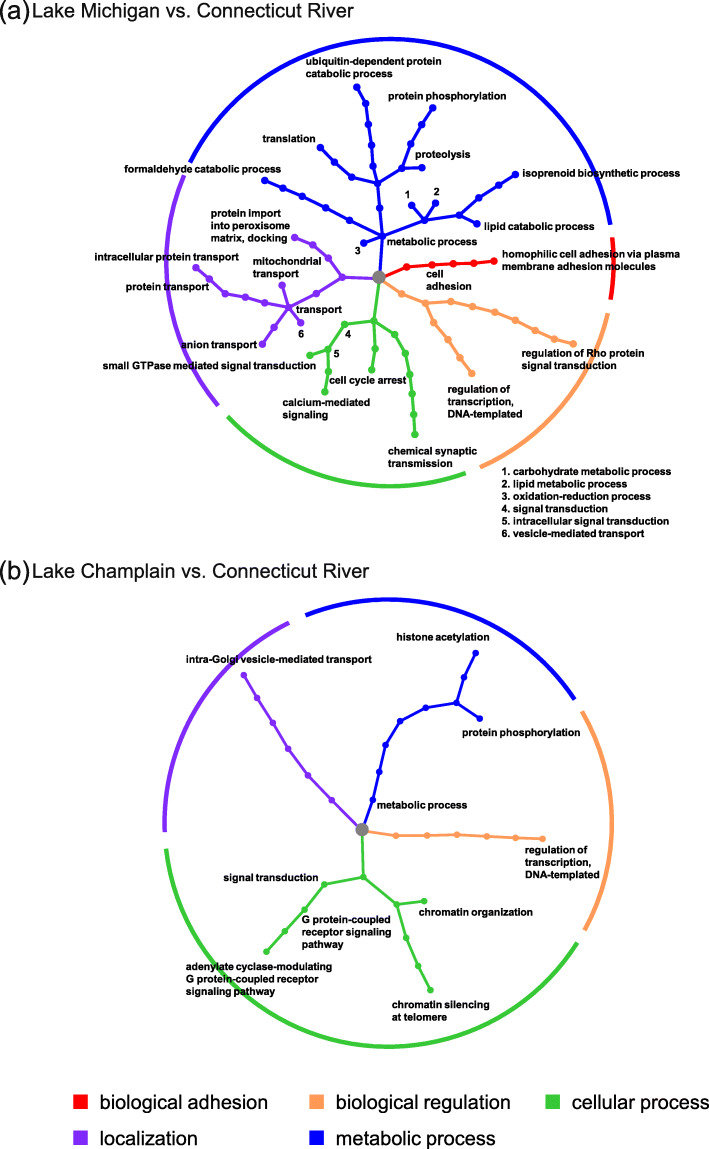
Gene ontology (GO) hierarchy networks. The GO hierarchy networks are constructed from GO terms associated with two or more outlier genes in the comparisons between Lake Michigan and Connecticut River (**a**) and between Lake Champlain and Connecticut River (**b**). Branch and node colors indicate the biological process child term to which distal nodes belong and the central grey node represents the biological process level of the GO hierarchy

With GO terms associated with outlier genes (Table S4), we identified 57 and 26 biological processes from GO hierarchy networks in comparisons between Lake Michigan and Connecticut River and between Lake Champlain and Connecticut River, respectively, among which 13 were common to both pairwise comparisons (Fig. 3, Fig. S7). According to the hierarchy networks of GO terms associated with one (Fig. S7) and more than one (Fig. 3) outlier gene, all biological processes can be categorized into six broad groups: biological adhesion, biological regulation, cellular process, localization, metabolic process, and growth (note that “growth” is in Fig. S7). Two of these biological processes, biological adhesion (Fig. 3) and growth (Fig. S7), only appear when comparing Lake Michigan and Connecticut River sea lamprey, suggesting that Lake Michigan sea lamprey may have adapted to their novel, introduced range by adjusting their growth (Fig. S7) and cell adhesion-mediated signal transduction pathways (Fig. 3). In contrast, the other four groups, cellular process, metabolic process, localization, and biological regulation, are common in both pairwise comparisons, with cellular and metabolic processes containing the largest number of biological processes (Fig. 3, Fig. S7). These four groups of biological processes may potentially indicate that the adaptation to the recently colonized, freshwater environments in Lake Michigan and Lake Champlain populations may be achieved through changes in DNA transcription and translation, cellular component organization, signal transduction, within-cell transport, and metabolism (Fig. 3, Fig. S7). Among biological processes corresponding to GO terms associated with more than one outlier gene, regulation of DNA transcription was common to both pairwise comparisons, while translation was only detected in comparison between Lake Michigan and Connecticut River (Fig. 3). This difference may reflect different evolutionary strategies the two recently colonized sea lamprey populations adopt to adapt to their novel environments, one of which is mediated exclusively at the transcriptomic level while the other is mediated through both DNA transcription and translation.

### Outlier genes underlying key life history differences and bioenergetics

**Fig. 4:**
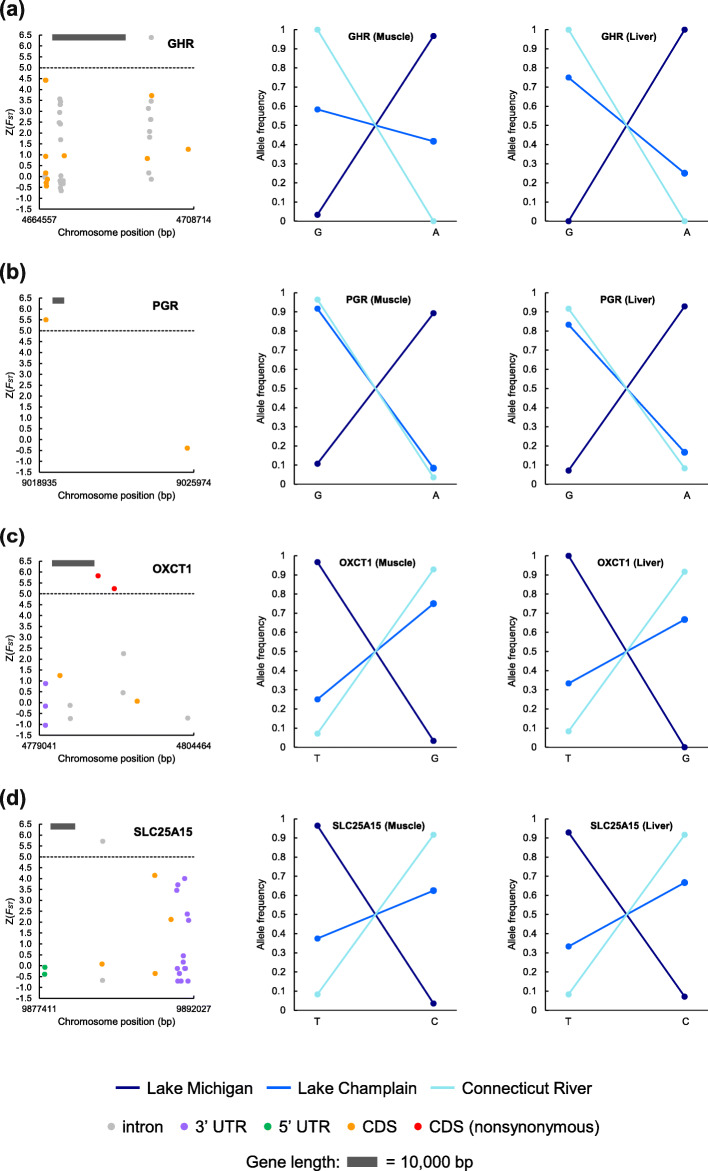
Z-transformed *F*_*ST*_, genomic regions, and allele frequencies of SNPs on outlier genes. Z(*F*_*ST*_) of all SNPs on outlier genes identified with muscle samples that may contribute to adaptation to novel environments (i.e.*,* growth, reproduction, bioenergetics) in sea lamprey from Lake Michigan, Lake Champlain, and Connecticut River are shown along corresponding chromosomes with SNPs located on introns, untranslated regions (UTR), and coding regions (CDS) indicated with different colors (left panels). Shifts in allele frequency of SNPs with the highest Z(*F*_*ST*_) or causing nonsynonymous mutations on outlier genes in Lake Michigan, Lake Champlain, and Connecticut River populations are almost identical across different tissue types (i.e.*,* muscle and liver shown in middle and right panels, respectively). The dark grey horizontal bar on the top left of the left panel represents the length of an outlier gene. *GHR* is associated with growth (**a**), *PGR* with reproduction (**b**), and *OXCT1* and *SLC25A15* with bioenergetics (**c**, **d**)

Six out of 121 outlier genes in comparison between Lake Michigan and Connecticut River (i.e.*, GHR*, *PGR*, *TTC25*, *STARD10*, *OXCT1*, *SLC25A15*) and two out of 43 outlier genes in comparison between Lake Champlain and Connecticut River (i.e.*, DIN4*, *PYGL*) may potentially explain changes in growth, reproduction, and bioenergetics in sea lamprey in response to novel ecological conditions (Fig. 4, Fig. S8). For all eight of these outlier genes, both *F*_*ST*_ and nucleotide diversity between populations increased while nucleotide diversity within populations decreased at outlier SNPs, further suggesting that genetic differentiation at these genes are driven by local adaptation (Fig. S9) [35].

*GHR*, which has a Z(*F*_*ST*_) of 6.39 and codes for a transmembrane receptor for growth hormone, can directly affect the growth rate of fish [36] (Fig. 4a). Lake Michigan and Connecticut River populations are fixed for alternative alleles at this gene (Fig. 4a). *PGR*, which has a Z(*F*_*ST*_) of 5.51 and encodes a nuclear progesterone receptor, plays a crucial role in the development of testis, the arrangement of spermatogenic cysts, sperm count, sperm motility, and ovulation [37, 38] (Fig. 4b). Lake Champlain and Connecticut River are almost fixed for the same *PGR* allele, while Lake Michigan is nearly fixed for the other (Fig. 4b). Two additional outlier genes, *STARD10* (Z(*F*_*ST*_) = 5.05), encoding a STAR-related lipid transfer protein, and cilia-related *TTC25* (Z(*F*_*ST*_) = 6.13), encoding a tetratricopeptide repeat domain-containing protein, may also affect the motility, maturation, or fertilization ability of sperm [39–42] (Fig. S8a, b).

We next identified a number of outlier genes directly related to bioenergetics. We first identified a gene containing nonsynonymous outlier SNPs when comparing Lake Michigan and Connecticut River populations, *OXCT1*, which has a Z(*F*_*ST*_) of 5.83 and codes for enzymes essential for the catabolism of ketone bodies that serve as an important fuel during starvation (Fig. 4c, Table S2) [43, 44]. Since ketone bodies serve as a primary fuel during starvation, functional differences at this gene may suggest a lower abundance or lower energetic quality of food in Lake Michigan. Another outlier gene containing nonsynonymous outlier SNPs, *PYGL*, was detected in the comparison between Lake Champlain and Connecticut River with a Z(*F*_*ST*_) of 5.05, which encodes an enzyme that promotes the release of glucose-1-phosphate from liver glycogen stores (Fig. S8c, Table S2) [45]. An additional gene containing synonymous outlier SNPs detected in comparison between Lake Champlain and Connecticut River with a Z(*F*_*ST*_) of 5.05, *DIN4*, is found to be activated under sugar starvation (Fig. S8d, Table S2) [46]. Metabolisms of ketone bodies and glycogen, which may compensate for energy deficiency under starvation, are potential indicators of adaptation to a different (and potentially lower quality) food supply in Lake Michigan and Lake Champlain.

Lastly, *SLC25A15*, which is detected in comparison between Lake Michigan and Connecticut River with a Z(*F*_*ST*_) of 5.72, codes for an ornithine mitochondrial transporter, which is a key component in urea cycle essential for nitrogen metabolism, and may suggest a shift in diet [47] (Fig. 4d). It has been shown that the urea excretion rate of sea lamprey removed from sharks is ~ 5 to 30 times higher than that of those removed from rainbow trout [48], suggesting that urea excretion in sea lamprey is affected by what prey they are feeding on. Thus, adaptive differentiation at *SLC25A15* may be a reflection of changes in sea lamprey’s prey and their capacity of adjusting nitrogen metabolism depending on which host fishes are available. Additionally, *SLC25A15* could also be potentially related to osmoregulation, ammonia detoxification, or even as a response to TFM treatment since the protein is located within mitochondria [30].

## Discussion

### Genetic differentiation and genetic diversity

A total of 121 outlier genes were identified in the comparison between Lake Michigan and Connecticut River and a total of 43 outlier genes were identified in the comparison between Lake Champlain and Connecticut River. While the highest genome-wide level of genetic differentiation across all loci were detected in the comparison between Lake Michigan and Lake Champlain (mean *F*_*ST*_ = 0.150), not a single outlier SNP was found between these two entirely-freshwater populations (Fig. S4a). These results were not driven by differences in sample sizes, read depth and numbers of loci in common, or insufficient power to detect outliers (Fig. S5). This striking result suggests that sea lamprey in Lake Michigan and Lake Champlain may have experienced parallel evolution whereby the differences in ecological conditions between the two lakes may not be large enough to exert strong differential selection when adapting to the novel environment. Furthermore, shared coancestry among Lake Michigan and Lake Champlain populations may further decrease the likelihood of detecting outliers between these populations. Parallel evolution and shared coanscestry are not mutually exclusive processes (i.e.*,* both phenomena could be at work in this system), yet we think the absence of outliers in the comparison between Lake Michigan and Lake Champlain may have been more likely to be driven by relatively high coancestry. Nevertheless, the two entirely-freshwater populations have likely experienced moderate levels of genetic drift, either via founder effects or higher variance in reproductive success, given the observation that mean *F*_*ST*_ was highest between these two populations.

The average *F*_*ST*_ of all three pairwise population comparisons was moderately high (Lake Michigan vs. Lake Champlain: mean *F*_*ST*_ = 0.150; Lake Michigan vs. Connecticut River: mean *F*_*ST*_ = 0.098; Lake Champlain vs. Connecticut River: mean *F*_*ST*_ = 0.123) and sea lamprey collected from Lake Michigan, Lake Champlain and Connecticut River clearly clustered into three distinct groups (Fig. 1b), suggesting that there is little ongoing gene flow among these populations. Genome-wide measurements of genetic diversity (measured as observed heterozygosity; *Ho*) were significantly different from each other (*p*-value < 0.0001 for all three pairwise comparisons), with the highest genetic diversity in the native-range Connecticut River population, followed by the Lake Michigan and Lake Champlain populations (Fig. 1c, Fig. S2). The reduction in genome-wide levels of genetic diversity in Lake Michigan and Lake Champlain are likely to be a result of founder effects; only a small number of sea lamprey may have been able to successfully colonize these environments [49].

### Candidate genes underlying rapid genetic adaptation

Invasive sea lamprey in the Great Lakes exhibit a faster growth rate, a shorter larval stage, a smaller adult body size, and a lower fecundity [25, 26]. Sea lamprey have very large effective population sizes, so we suggest that, at genes related to these changes, selection is most likely to have acted on the standing genetic variation already present within the population and did not depend on de novo mutations. Body size has been repeatedly shown to be a highly heritable trait in fishes [50, 51]. *GHR* (growth hormone receptor) was identified as an outlier in the comparison between Lake Michigan and Connecticut River. *GHR* encodes a transmembrane growth hormone receptor. The dimerization of the growth hormone receptor occurs upon the binding of growth hormone, and a signal transduction pathway involved in growth is turned on. As such, *GHR* plays a crucial role in fish growth [36] and may account for the faster growth rate and smaller adult size of sea lamprey colonizing the Great Lakes and Lake Champlain. Additionally, selection at the outlier genes, *PGR*, *STARD10*, and *TTC25*, could explain the lower fecundity observed in Lake Michigan sea lamprey since these genes are known to affect ovulation and sperm production and quality [37, 38]. The Lake Champlain population showed no evidence of changes in allele frequencies compared to the native Connecticut River population at *PGR* and *STARD10*, but did show evidence of adaptive evolution at *TTC25* (Fig. 4b, Fig. S8a, b). Together, these candidate genes may underlie a novel life history strategy that optimizes the classic tradeoff between growth and reproduction [52]. Lake Champlain and Connecticut River populations are genetically distinct, but no outlier gene related to life history traits (e.g.*,* growth, reproduction) was detected in the comparison between these two populations. This result may suggest that changes in life history strategies essential for sea lamprey’s colonization in Lake Champlain may be mediated by plasticity or the genetic differentiation between these two populations driving changes in life history strategies is not strong enough to be detected.

In the Great Lakes, sea lamprey spend more time feeding on a single host, and more sea lamprey are found to attach to one adult fish than in the Atlantic Ocean [25, 26]. This observation could be driven by the high ratio of sea lamprey to host fishes, suggesting that food sources may have been limited in the invasive range given the high abundance of sea lamprey prior to treatment with lampricides. Interestingly, we find two candidate genes containing nonsynonymous outlier SNPs (i.e.*, OXCT1* and *PYGL*) and one containing synonymous outlier SNPs (i.e.*, DIN4*) that support this hypothesis at the genomic level. *OXCT1* is involved in the catabolism of a primary fuel under starvation (i.e.*,* ketone bodies), *PYGL* participates in the production of glucose from liver glycogen stores, and *DIN4* is activated under sugar starvation. While *OXCT1* is identified in the comparison between Lake Michigan and Connecticut River, *PYGL* and *DIN4* are detected in the comparison between Lake Champlain and Connecticut River, suggesting that sea lamprey in both Lake Michigan and Lake Champlain have responded to the selection imposed by the differences in food sources and availability in these novel, freshwater environments.

Instead of migrating to the ocean, Lake Michigan and Lake Champlain sea lamprey spend their entire life in freshwater and never acclimate to salt water environments. Previous work has shown that 11-deoxycortisol, Na-K-Cl cotransporter 1, and Na^+^-K^+^-ATPase may engage in sea lamprey’s osmoregulation [53–55]. We would expect to detect genetic differentiation at genes encoding these components involved in osmoregulation, but we did not find any direct evidence at the genetic level (i.e.*,* SNPs) to support this hypothesis. However, both growth hormone and glycogen metabolism have been shown to be related to seawater adaptation [56–58], suggesting that *GHR* and *PYGL* may also play a role in the successful colonization of freshwater environments. Our inability to uncover a clearer genetic signal related to sea lamprey’s adaptation to freshwater environments could be due to a lack of power (e.g., sample sizes). Alternatively, sea lamprey may respond to changes in salinity plastically by differentially expressing relevant genes at the transcriptomic level, and the selection imposed by differences in salinity may not be strong enough to shift allele frequencies among populations at the genomic level [54, 59].

### Future directions

While we have identified a series of outlier genes that may be driving sea lamprey’s rapid adaption to novel environments, several outstanding questions remain. Although our study identified outlier genes that are likely driving rapid adaptation at the genomic level, we do not know yet how these genes perform at the proteomic and metabolic level to support variation at the physiological, individual, and population level in recently colonized sea lamprey populations. Additionally, four large classes of genes were found to be related to cell cycle progression, cellular component assembly, cellular transporting, and cellular signaling. These genes together regulate the replication, transcription, and translation of DNA, the assembly of chromatin and ribosome, and some cellular enzymatic reactions. How these genes work concurrently and coordinate to help sea lamprey colonize new environments remains unknown and merits further investigation. Lastly, these genetic changes in life history traits may be driven by selection to escape treatment from the lampricide 3-trifluoromethyl-4-nitrophenol (TFM), which is applied in both Lake Michigan and Lake Champlain. Recent theoretical work suggests that a rapid decrease in the size and age at metamorphosis could be driven by TFM-induced selection [60]. Determining whether these changes in life history associated genes we documented here are driven by the natural environment, treatment with TFM, or both will be important for the successful management of invasive sea lamprey.

We cannot exclude the possibility that some of the outlier genes detected using *F*_*ST*_ are false positives, and caution is warranted when using *F*_*ST*_ as an indicator for local adaptation [35, 61, 62]. Although *F*_*ST*_ has been widely used to assess genetic differentiation between populations, there are some caveats for using it to detect local adaptation. The correlated coancestry among organisms living in the long one-dimensional landscapes may generate many false positives [61, 62]. It is therefore important to take the characteristics of the study system into consideration when using *F*_*ST*_ as an estimator for genetic differentiation. Furthermore, a *F*_*ST*_ outlier may not necessarily indicate local adaptation because it could also be driven by global adaptation or genetic drift [35]. Therefore, we suggest a combination of *F*_*ST*_, *AFD*, kNN-based approach, and nucleotide diversity within and between populations as an approach to discriminate genetic differentiation arising from local adaptation from that driven by other mechanisms. We also recommend using strict filtering criteria (here outliers were only considered if *F*_*ST*_ was greater than five standard deviations from the mean). This process almost certainly increases the type II error rate (i.e.*,* many true outlier loci remain undetected in our data set), but importantly serves to decrease the type I error rate (i.e.*,* reduces the number of loci which are identified as outliers but, in reality, have not been driven by a response to selection).

Biological invasion has been closely observed in many systems [1, 2, 6, 63, 64], and can have devastating effects on agriculture, fisheries, ecosystem biodiversity, and public health. The colonization of introduced species into novel environments that are vastly different from their native range in both biotic and abiotic conditions could be facilitated by plasticity or rapid adaptation [63, 64]. To gain insight into how invasive species rapidly adapt to novel environments, further investigation is required to determine whether selection acts on standing genetic variation or new mutations and how various genetic and evolutionary processes (e.g.*,* bottlenecks, hybridization, polyploidy, genome modification) may affect rapid adaptation [2]. As global climate change tends to increase the uncertainty in evaluating the cost of biological invasion [64, 65], a better understanding of what genetic factors and how they promote invasion is essential for illuminating integrative strategies to invasive species control and ecosystem conservation and restoration.

## Conclusions

Utilizing genome-wide SNPs called from RNA-seq data, we found that two sea lamprey populations lost genome-wide genetic diversity when colonizing novel, entirely freshwater environments. Evidence of a response to selection at eight outlier genes suggest that sea lamprey rapidly adapted to their novel, freshwater environments with changes in growth, reproduction, and bioenergetics. Overall, our study enhances our understanding of the genes underlying sea lamprey’s life history traits, which may aid with invasive species control in the Great Lakes, and also demonstrates how introduced species can be a useful study system for shedding light on rapid genetic adaptation.

## Methods

### Sample collection and experimental design

We collected 565 larval sea lamprey from the Manistee River in Lake Michigan, 517 larval sea lamprey from Corbeau Creek and the LaPlatte River in Lake Champlain, and 404 larval sea lamprey from the Connecticut River in 2016 (Fig. 1a), among which 283 individuals from each population were used to test the sensitivity of sea lamprey to TFM (see [30] for details). We sampled a total of 43 individuals (Lake Michigan: *n* = 16, Lake Champlain: *n* = 13, Connecticut River: *n* = 14) for RNA-seq after a four-month acclimation period in a common environment at the Aquaculture Research Lab at Purdue University (see Methods of Supplementary Information for details). All samples were collected on the same day and immediately frozen with liquid nitrogen in cryovials, which were later transferred to a −80 °C freezer. These individuals were previously used to determine whether invasive sea lamprey are evolving resistance to the pesticide TFM (see [30] for details).

### RNA-seq and single nucleotide polymorphism (SNP) calling

To extract tissue-specific (i.e.*,* muscle and liver tissues) mRNA, we transferred samples from the −80 °C freezer into RNAlater-ICE solution (Ambion Inc., Austin, TX, USA), with a ratio of ten volumes of solution to one volume of sample mass, and kept the tissue samples in the solution for 16 h at −20 °C. We next weighed the thawed tissues, submerged them in Qiagen (Chatsworth, CA) lysis buffer, and homogenized them using a rotor-stator homogenizer. After extracting mRNA from 43 muscle and 19 liver tissue samples using Qiagen RNeasy Mini Kit, we prepared mRNA libraries and sequenced them at the Purdue Genomics Core Facility to generate a total of 1,385,780,178 paired-end RNA-seq reads (average 22,351,293 reads per sample) (Table S1). We trimmed RNA-seq reads using Trimmomatic v0.36 [66] with parameters suggested by MacManes [67].

With trimmed RNA-seq reads, we called SNPs with the variant discovery pipeline provided by the Genome Analysis Toolkit (GATK 3.8) [68]. We first called SNPs following the joint genotyping workflow for muscle (*n* = 43; Lake Michigan: *n* = 16, Lake Champlain: *n* = 13, Connecticut River: *n* = 14) and liver (*n* = 19; Lake Michigan: *n* = 7, Lake Champlain: *n* = 6, Connecticut River: *n* = 6) tissue samples, separately [69]. We started by mapping RNA-seq reads to the sea lamprey genome [70] following the STAR 2-pass alignment steps. We set --outFilterMultimapNmax to 1, --outSJfilterReads to Unique, --alignEndsType to EndToEnd, --chimMainSegmentMultNmax to 1, --limitGenomeGenerateRAM to 250,000,000,000, --sjdbOverhang to 149, --runThreadN to 19, and --limitSjdbInsertNsj to 25,000,000. All other parameters were set to default values [68]. By adding read groups, sorting, marking duplicates, and creating indices, we obtained BAM files from SAM files generated by the STAR 2-pass alignment steps. After generating BAM files, we applied the GATK tool, SplitNCigarReads, to BAM files, to split reads into exon segments and cut sequences extending to intronic regions. Next, we called SNPs using the GATK tool, HaplotypeCaller, in which we set --genotyping_mode to DISCOVERY, --emitRefConfidence to GVCF, --variant_index_type to LINEAR, --variant_index_parameter to 128,000, -pairHMM to VECTOR_LOGLESS_CACHING, -ploidy to 2, and -maxAltAlleles to 100. Lastly, we performed joint genotyping using the GATK tool, GenotypeGVCFs, with --max_alternate_alleles set to 100, and removed all indels. To validate SNPs called from the GATK joint genotyping workflow with RNA-seq reads, we additionally called SNPs following the RNA-seq variant calling pipeline. The differences between the joint genotyping workflow and the RNA-seq variant calling pipeline begin with the application of HaplotypeCaller. In comparison to the joint genotyping workflow, we supplied one sample to HaplotypeCaller at a time when applying the RNA-seq variant calling pipeline, in which we picked the option -dontUseSoftClippedBases, and set -stand_call_conf to 20.0 and -maxAltAlleles to 100. Finally, we filtered variants using the GATK tool, VariantFiltration, by setting -window to 35 and -cluster to 3 and applying filters “FS > 30.0” and “QD < 2.0”, and removed indels.

We used the SNPs that were identified in both the GATK joint genotyping workflow and the RNA-seq pipeline in all subsequent analyses. For a subset of 18 individuals, we sequenced both muscle and liver tissues. By independently calling SNPs with muscle and liver tissue samples from the same individuals, we were able to validate our SNP calling pipeline (i.e.*,* we used genotypes called from liver tissue as a validation for those called from muscle tissue, see Fig. 4). Genotypes were identical between muscle and liver tissues of the same individual at 93% of loci. For each individual, genotypes called at loci with fewer than five reads were set as missing values. Muscle samples from two individuals 401 (Lake Champlain) and 402 (Lake Michigan) (Table S1) had over 90% missing data and thus were excluded from further analyses. Finally, we retained loci genotyped across at least 80% of all muscle samples or all liver samples and removed loci with a minor allele frequency smaller than 0.025 (i.e.*,* an alternative allele had to be present in at least two out of 41 muscle samples and one out of 19 liver samples).

### Assessment of population structure, Hardy-Weinberg equilibrium, and genetic differentiation

We first tested population structure using the program STRUCTURE (version 2.3) [31–34] using the options “admixture model” and “correlated allele frequencies among populations”. When running STRUCTURE, we set the length of burnin period to 50,000, the number of MCMC reps after burnin to 50,000, and the number of populations assumed (*K*) to 2 to 10. Since sampling locations can assist the clustering for datasets with a relatively weak signal of structure when used as prior information, we chose to use location information (i.e.*,* Lake Michigan, Lake Champlain, Connecticut River).

With the 41 muscle samples (Lake Michigan: *n* = 15; Lake Champlain: *n* = 12; Connecticut River: *n* = 14), we first excluded loci out of Hardy-Weinberg equilibrium identified using the package *HWxtest* in R 3.6.1 (likelihood ratio *p*-value < 0.05) [71, 72] for each population. We calculated observed heterozygosity at each locus for each population with SNPs that were in Hardy-Weinberg equilibrium and common to all three populations. Sea lamprey have 99 chromosomes [70], 90 of which are assembled, so we next calculated observed heterozygosity of each of the 90 assembled chromosomes for each population by averaging observed heterozygosity across all loci within each chromosome in a population. We ran randomization tests to test whether observed heterozygosity at each locus and each chromosome varied among the Lake Michigan, Lake Champlain, and Connecticut River populations. For a randomization test, we pooled the heterozygosity of all samples from two populations, and randomly generated 10,000 pairs of subsamples of heterozygosity with two subsample sizes equal to sizes of two populations in a particular comparison by sampling from the pooled heterozygosity without replacement. For each of the 10,000 draws, we calculated the absolute difference between the mean heterozygosity of two subsamples as our test statistic, and compared the absolute difference between each pair of mean subsampled heterozygosity to the observed differences in heterozygosity (Fig. S2). Additionally, we calculated nucleotide diversity within each of the three populations (i.e.*,* Lake Michigan, Lake Champlain, Connecticut River; *π*) and between all three pairwise comparisons (i.e.*,* Lake Michigan vs. Lake Champlain, Lake Michigan vs. Connecticut River, Lake Champlain vs. Connecticut River) at each locus using the R package *PopGenome* [73]. We averaged nucleotide diversity within populations across all loci on a chromosome and compared mean observed heterozygosity and mean nucleotide diversity for each chromosome.

We next calculated pairwise, unbiased *F*_*ST*_ for each locus in Hardy-Weinberg equilibrium among all three populations (i.e.*,* Lake Michigan vs. Lake Champlain, Lake Michigan vs. Connecticut River, and Lake Champlain vs. Connecticut River) using vcftools (version 0.1.16) [74]. To assess genetic differentiation at the population level, we averaged *F*_*ST*_ across all loci in each comparison. To detect significant outliers, we z-transformed *F*_*ST*_ in each comparison to get Z(*F*_*ST*_), and defined loci with *F*_*ST*_ greater than five standard deviations from the mean as outlier loci (i.e.*,* Z(*F*_*ST*_) > 5) [6]. To validate outliers identified by *F*_*ST*_, we also calculated pairwise allele frequency difference (*AFD*) at each locus, z-transformed *AFD* to get Z (*AFD*), and compared analysis results based on *F*_*ST*_ and *AFD* [75]. We additionally ran genome scans based on *k*-nearest neighbor (kNN) techniques to assess whether an outlier locus is driven by selection [76]. With pairwise *F*_*ST*_ of all population comparisons, the kNN-based approach, which is implemented in the R package *PopGenome* [73, 77], assigns all SNPs to multidimensional space [76]. SNPs that cluster together are located on potential genomic regions evolving neutrally, while SNPs that are detected as outliers in this space are likely to be under selection. The entry of the ∆*F*_*ST*_ vector, which is calculated by subtracting the medoid from the pairwise *F*_*ST*_ vector, indicates local adaptation when positive but introgression or selection reducing genetic divergence when negative [76]. After identifying outlier SNPs, we identified corresponding genes (i.e.*,* outlier genes) on which outlier loci were located by mapping outlier loci to the sea lamprey genome [70] and calculated the allele frequency at each outlier locus. Lastly, we constructed gene ontology (GO) hierarchy networks of GO terms in the domain of “biological process” associated with outlier genes, which were retrieved from Gene Ontology and GO Annotations (https://www.ebi.ac.uk/QuickGO/), for each pairwise comparison using the *metacoder* package [78].

### Candidate genes

By examining the position of outlier loci on the annotated sea lamprey genome [70], we determined whether an outlier SNP was located on introns, coding regions (CDS), or untranslated regions (3′ or 5′ UTR). For those on CDS of an outlier gene, we found open reading frames using the NCBI Open Reading Frame Finder by setting minimal ORF length (nt) to 30 and ORF start codon to “ATG and alternative initiation codons”. When there were multiple open reading frames for a coding region, we picked the longest one [6]. For the identified open reading frame, we found the position where the outlier SNP was located, set the outlier locus to reference (i.e.*,* ref ORF) and alternative (i.e.*,* alt ORF) alleles, and translated the ref and alt ORFs for the same coding region into reference and alternative amino acid sequences, respectively. If reference and alternative amino acid sequences were identical, the SNP located at the outlier locus was classified as a synonymous change; otherwise, the SNP was classified as nonsynonymous. All outlier genes were assigned to one of two categories: those containing outlier SNPs causing synonymous substitutions or outlier SNPs causing nonsynonymous substitutions.

## Supplementary Information

**Additional file 1: Figure S1.** Population structure of Lake Michigan, Lake Champlain, and Connecticut River sea lamprey (*K* = 2, 4, 10). Likelihood values for *K* = 2, *K* = 4 and *K* = 10 are −11,693,170, −11,023,345, and −11,043,300, respectively. **Figure S2.** Randomization tests on observed heterozygosity among three populations. Observed heterozygosity (*Ho*) of each chromosome (by chr; sea lamprey have 99 chromosomes, 90 of which are assembled) and at SNPs (by SNP) calculated using 346,280 SNPs across the genome that are in Hardy-Weinberg equilibrium and in common to Lake Michigan (LM), Lake Champlain (LC) and Connecticut River (CT) populations differs significantly between each pairwise comparison (LM vs. LC, LM vs. CT, LC vs. CT; *p* < 0.0001 for all three pairwise comparisons). **Figure S3.** Observed heterozygosity and nucleotide diversity (*π*) of three populations. Sea lamprey have 99 chromosomes, of which the first 90 are assembled. Mean observed heterozygosity and mean nucleotide diversity across each chromosome are highly correlated in Lake Michigan (a), Lake Champlain (b), and Connecticut River (c) populations. Mean nucleotide diversity across the genome is indicated by dashed lines and nucleotide diversity of chromosomes is indicated by points (d). Nucleotide diversity (*π*) shows a similar pattern as observed heterozygosity, which is the highest in Connecticut River, followed by Lake Michigan and Lake Champlain (d). **Figure S4.** Z(*F*_*ST*_) along 90 chromosomes in three pairwise comparisons. Sea lamprey have 99 chromosomes, 90 of which are assembled. In comparison to Fig. 2a & b, this figure shows Z(*F*_*ST*_) of all SNPs in comparisons between Lake Michigan and Lake Champlain (a), Lake Michigan and Connecticut River (b), and Lake Champlain and Connecticut River (c), rather than mean Z(*F*_*ST*_) (Fig. 2 main text) of SNPs on genes without outlier SNPs. Alternating colors purple and grey represent Z(*F*_*ST*_) of SNPs on different chromosomes and alternating colors blue and black represent Z(*F*_*ST*_) of SNPs on neighboring outlier genes. Outlier SNPs with Z(*F*_*ST*_) but in purple and grey are those that are not located on any annotated gene. **Figure S5.** Mean read depth of each population and number of shared loci in each pairwise comparison. Mean read depths in three populations are almost identical (a) and numbers of shared loci used to calculate *F*_*ST*_ and *AFD* for three pairwise comparisons among Lake Michigan (LM), Lake Champlain (LC), and Connecticut River (CT) are almost identical (b). **Figure S6.** Correlations between *F*_*ST*_ and *AFD* in three pairwise comparisons. **Figure S7.** Gene ontology (GO) hierarchy networks. The GO hierarchy networks are constructed from GO terms associated with one outlier gene in comparisons between Lake Michigan and Connecticut River (a) and between Lake Champlain and Connecticut River (b) using the *metacoder* package in R. Branch and node colors indicate the biological process child term to which distal nodes belong and the central grey node represents the biological process level of the GO hierarchy. **Figure S8.** Z-transformed *F*_*ST*_ (i.e., Z(*F*_*ST*_)), genomic regions, and allele frequency of SNPs for additional outlier genes. Z(*F*_*ST*_) of all SNPs on outlier genes identified with muscle samples that may contribute to adaptation to local environments (i.e., reproduction, bioenergetics) in sea lamprey are shown along corresponding chromosomes with SNPs on introns, untranslated regions (UTR), and coding regions (CDS) indicated in different colors (left panels). Shifts in allele frequency of SNPs with the highest Z(*F*_*ST*_) or causing nonsynonymous mutations on outlier genes in Lake Michigan, Lake Champlain, and Connecticut River populations are almost identical across different tissue types (i.e., muscle and liver shown in middle and right panels, respectively). The dark grey horizontal bar on the top left of the left panel represents the length of an outlier gene. *STARD10* and *TTC25* are associated with reproduction (a, b), and *PYGL* and *DIN4* with bioenergetics (c, d). **Figure S9.** *F*_*ST*_, nucleotide diversity within populations, and nucleotide diversity between populations at outlier and surrounding SNPs. Dashed lines represent average *F*_*ST*_ (black lines), nucleotide diversity within populations (blue lines), and nucleotide diversity between populations (red lines) of all SNPs 50 Kb upstream and downstream (i.e.*,* 100 Kb window) from the outlier SNP with the highest *F*_*ST*_. Points indicate *F*_*ST*_ (black), nucleotide diversity within populations (blue), and nucleotide diversity between populations (red) of outlier SNPs. Plots were qualitatively and nearly numerically identical for ±10 Kb, ±100 Kb, and ± 500 Kb windows (with the exception of *PGR* at the ±10 Kb and ± 500 Kb windows, which had slightly higher within population nucleotide diversity than the ±10 Kb and ± 500 Kb averages). Single window averages were used instead of a standard sliding window approach because RNA-seq data generates SNP loci that are highly clustered within, but not between, genic regions. Where necessary, points were jittered for visual clarity. At all eight outlier genes, *F*_*ST*_ and nucleotide diversity between populations tend to increase while nucleotide diversity within populations tends to decrease at outlier SNPs in compa_*ST*_rison to the average, suggesting that divergence at these genes is driven by local adaptation. **Table S1.** Summary of tissue samples used for RNA-seq. **Table S2.** Summary of outlier SNPs and outlier genes. LM vs. CT stands for the pairwise comparison between Lake Michigan and Connecticut River populations, and LC vs. CT stands for the pairwise comparison between Lake Champlain and Connecticut River populations. See Table S3 for full gene names. **Table S3.** Full gene names of outlier genes retrieved from https://david.ncifcrf.gov/list.jsp. **Table S4.** Gene ontology (GO) terms of biological process associated with outlier genes retrieved from https://www.ebi.ac.uk/QuickGO/.

## Data Availability

Code and scripts are available at https://github.com/ChristieLab/sea_lamprey_adaptation. Trimmed reads are available via https://www.ncbi.nlm.nih.gov/sra with BioProject accession number PRJNA667554.
