# Supplementary material for: Rapid genetic adaptation to recently colonized environments is driven by genes underlying life history traits
Source: BMC Genomics. 2021 Apr 14;22:269. doi: 10.1186/s12864-021-07553-x (PMC8048285; doi:10.1186/s12864-021-07553-x)
Supplement: Supplementary file 1 — Additional file 1: Figure S1. Population structure of Lake Michigan, Lake Champlain, and Connecticut River sea lamprey (K = 2, 4, 10). Likelihood values for K = 2, K = 4 and K = 10 are −11,693,170, −11,023,345, and −11,043,300, respectively. Figure S2. Randomization tests on observed heterozygosity among three populations. Observed heterozygosity (Ho) of each chromosome (by chr; sea lamprey have 99 chromosomes, 90 of which are assembled) and at SNPs (by SNP) calculated using 346,280 SNPs across the genome that are in Hardy-Weinberg equilibrium and in common to Lake Michigan (LM), Lake Champlain (LC) and Connecticut River (CT) populations differs significantly between each pairwise comparison (LM vs. LC, LM vs. CT, LC vs. CT; p < 0.0001 for all three pairwise comparisons). Figure S3. Observed heterozygosity and nucleotide diversity (π) of three populations. Sea lamprey have 99 chromosomes, of which the first 90 are assembled. Mean observed heterozygosity and mean nucleotide diversity across each chromosome are highly correlated in Lake Michigan (a), Lake Champlain (b), and Connecticut River (c) populations. Mean nucleotide diversity across the genome is indicated by dashed lines and nucleotide diversity of chromosomes is indicated by points (d). Nucleotide diversity (π) shows a similar pattern as observed heterozygosity, which is the highest in Connecticut River, followed by Lake Michigan and Lake Champlain (d). Figure S4. Z(FST) along 90 chromosomes in three pairwise comparisons. Sea lamprey have 99 chromosomes, 90 of which are assembled. In comparison to Fig. 2a & b, this figure shows Z(FST) of all SNPs in comparisons between Lake Michigan and Lake Champlain (a), Lake Michigan and Connecticut River (b), and Lake Champlain and Connecticut River (c), rather than mean Z(FST) (Fig. 2 main text) of SNPs on genes without outlier SNPs. Alternating colors purple and grey represent Z(FST) of SNPs on different chromosomes and alternating colors blue and black represent Z(FST [file 12864_2021_7553_MOESM1_ESM.docx]

**Rapid genetic adaptation to recently colonized environments is driven by genes underlying life history traits**

Xiaoshen Yin^a^, Alexander S. Martinez^a^, Maria S. Sepúlveda^b^, Mark R. Christie^a,b,*^

^a^Department of Biological Sciences, Purdue University; 915 W. State St., West Lafayette, Indiana 47907-2054, USA

^b^Department of Forestry and Natural Resources, Purdue University; 715 W. State St., West Lafayette, Indiana 47907-2054, USA

*To whom correspondence should be addressed: Mark Christie, 915 W. State Street, Department of Biological Sciences, Purdue University, West Lafayette, IN 47907-2054. Phone: (765) 494-2070, E-mail: [markchristie@purdue.edu](mailto:markchristie@purdue.edu).

# Supplementary Information

# Methods

## **Gene expression experiments**

After a four-month acclimation, we set up two gene expression trials, gene expression experiment 1 (GE 1) on November 21, 2016 and gene expression experiment 2 (GE 2) on November 30, 2016 at the Aquaculture Research Laboratory at Purdue University to obtain RNA-seq data. In GE 1, we allocated 12, 2.5-gallon glass tanks to each of three populations (*i.e.,* Lake Michigan, Lake Champlain and Connecticut River populations), totaling 36 tanks. Each tank received seven ammocoetes from the corresponding population, 12 tanks for each population were divided into three groups with four in each, and TFM concentrations in each group of four tanks were maintained at 0, 0.2 mg/L or 0.3 mg/L, respectively. In GE 2, we allocated 10, 2.5-gallon glass tanks to each population, totaling 30 tanks, and each tank received seven ammocoetes from the corresponding population. Two out of ten tanks for each population did not receive any TFM and served as control. The other eight tanks were evenly divided into two groups with TFM concentration of each group maintained at 0.2 mg/L or 0.3 mg/L. In both GE 1 and GE 2, we pre-filled each tank with 7 L of water, and control (0 mg/L TFM) and treatment (0.2 or 0.3 mg/L) tanks received an equal volume of water or TFM solution, respectively, to achieve the predetermined TFM concentration. After a one-hour acclimation, all ammocoetes were first exposed to corresponding TFM concentrations (*i.e.,* 0, 0.2 or 0.3 mg/L) for ten hours and then received a washout for two hours, mimicking the TFM stream application in practice. At hour 6 during the 12-hour gene expression trial (*i.e.,* 10-hour TFM exposure and 2-hour washout), we sampled one ammocoete from each tank and euthanized it in a lethal dose of MS-222, followed by weighing, measuring, and dissecting.

# Results

## **Identification of outlier genes by F_ST_, AFD, and kNN approach**

Correlation coefficients (*r*) between *F_ST_* and *AFD*, an alternative measure of genetic differentiation, ranged from 0.95 to 0.97 for the three pairwise comparisons among Lake Michigan, Lake Champlain, and Connecticut River (Fig. S6). In the comparison between Lake Michigan and Connecticut River, 402 out of 436 outlier SNPs detected according to *F_ST_* ranked as the top 436 with *AFD*, and all 209 outlier SNPs detected using *F_ST_* in comparison between Lake Champlain and Connecticut River were among the top 209 SNPs ranked by *AFD* (Table S2). The remaining 34 outlier SNPs identified by *F_ST_* in comparison between Lake Michigan and Connecticut River have Z(*AFD*) ranging from 4.41 to 4.55, which is close to our cutoff value of five standard deviations. Therefore, the comparison between *F_ST_* and *AFD* demonstrates that *F_ST_* is reliable in detecting genetic differentiation between populations to identify outlier SNPs. To test whether the outlier SNPs were driven by a response to selection, we additionally ran genome scans based on a *k*‐nearest neighbor (kNN) approach (see **Methods** in the main text for details on kNN techniques). According to the kNN-based genome scans, except for 16 out of 436 outlier SNPs in the comparison between Lake Michigan and Connecticut River and six out of 209 outlier SNPs in the comparison between Lake Champlain and Connecticut River, at which ∆*F_ST_* could not be calculated, all remaining outlier SNPs detected in these two pairwise comparisons have a positive ∆*F_ST_* ranging from 0.7 to 1, suggesting that genetic differentiation at these outlier SNPs is likely to be driven by selection (Table S2).

(a) *K* = 2


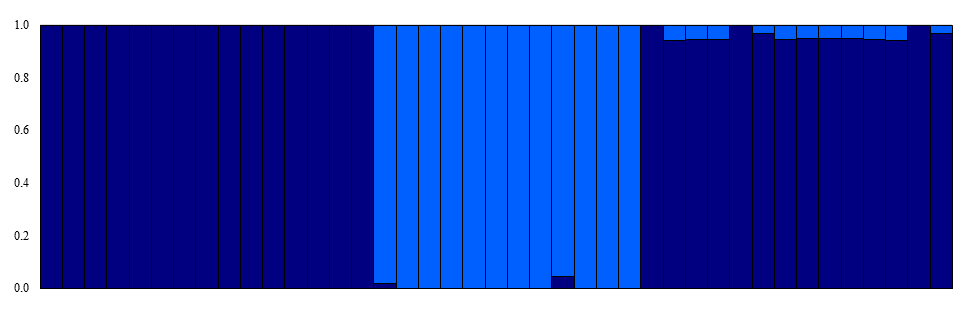


Lake Michigan Lake Champlain Connecticut River

Membership coefficient

(b) *K* = 4


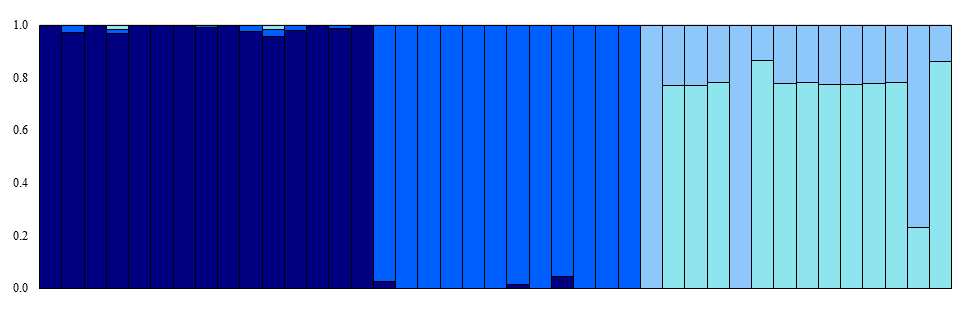


Lake Michigan Lake Champlain Connecticut River

Membership coefficient

(c) *K* = 10

**
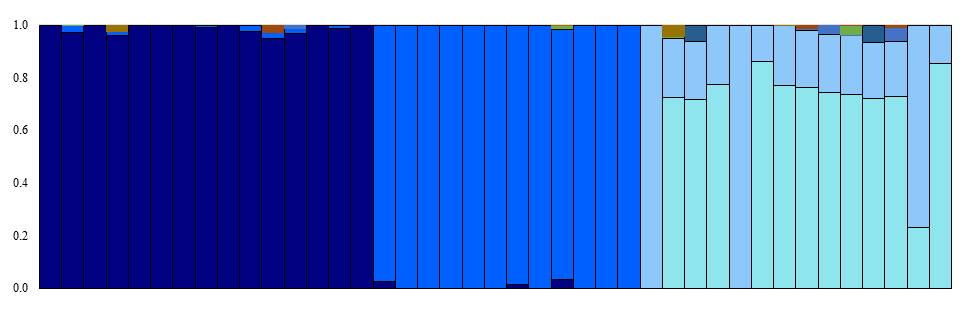
**

Lake Michigan Lake Champlain Connecticut River

Membership coefficient

**Figure S1.** Population structure of Lake Michigan, Lake Champlain, and Connecticut River sea lamprey (*K* = 2, 4, 10). Likelihood values for *K* = 2, *K* = 4 and *K* = 10 are -11693170, -11023345, and -11043300, respectively.


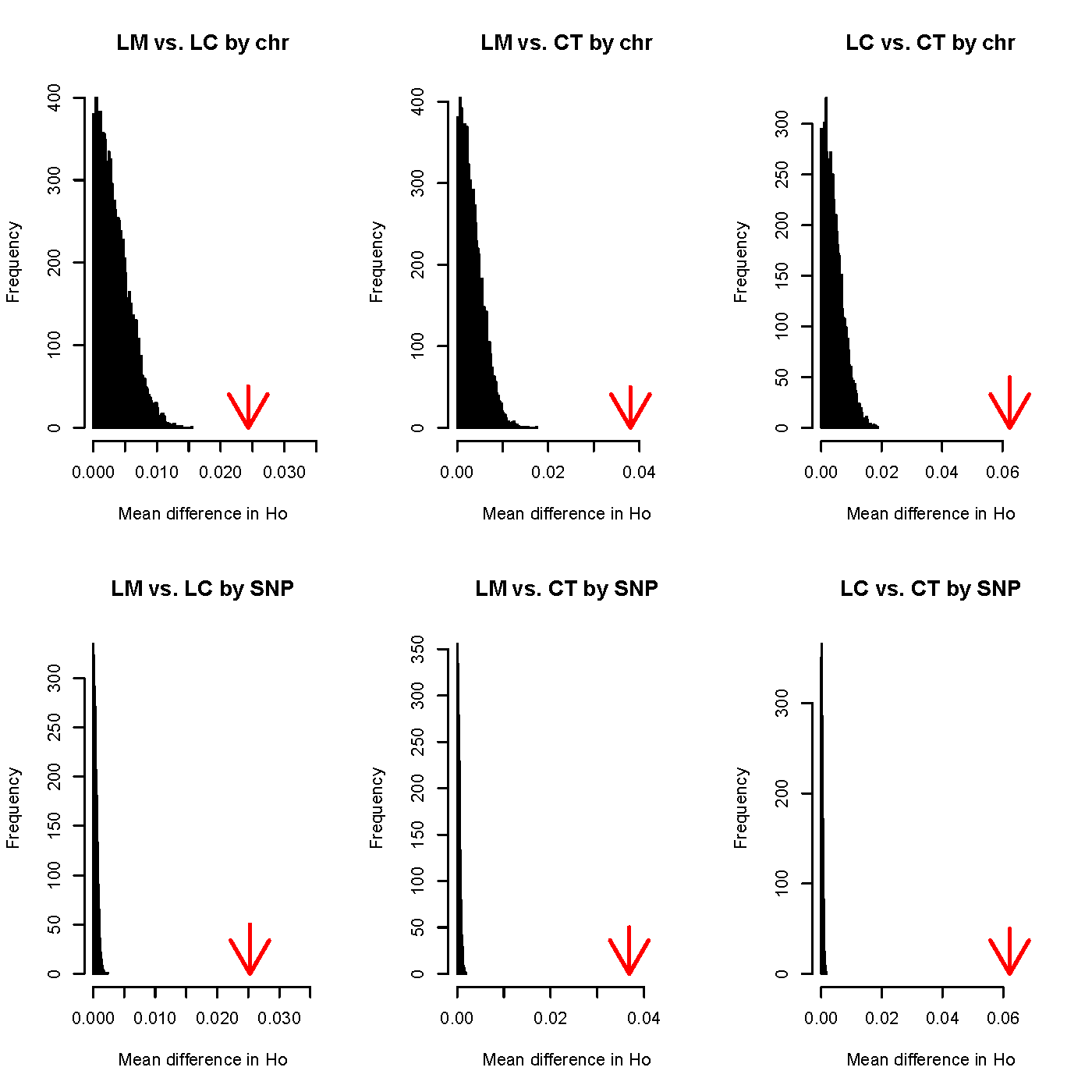


**Figure S2.** Randomization tests on observed heterozygosity among three populations. Observed heterozygosity (*Ho*) of each chromosome (by chr; sea lamprey have 99 chromosomes, 90 of which are assembled) and at SNPs (by SNP) calculated using 346,280 SNPs across the genome that are in Hardy-Weinberg equilibrium and in common to Lake Michigan (LM), Lake Champlain (LC) and Connecticut River (CT) populations differs significantly between each pairwise comparison (LM vs. LC, LM vs. CT, LC vs. CT; *p* < 0.0001 for all three pairwise comparisons).

(a) (b) (c)

(d)


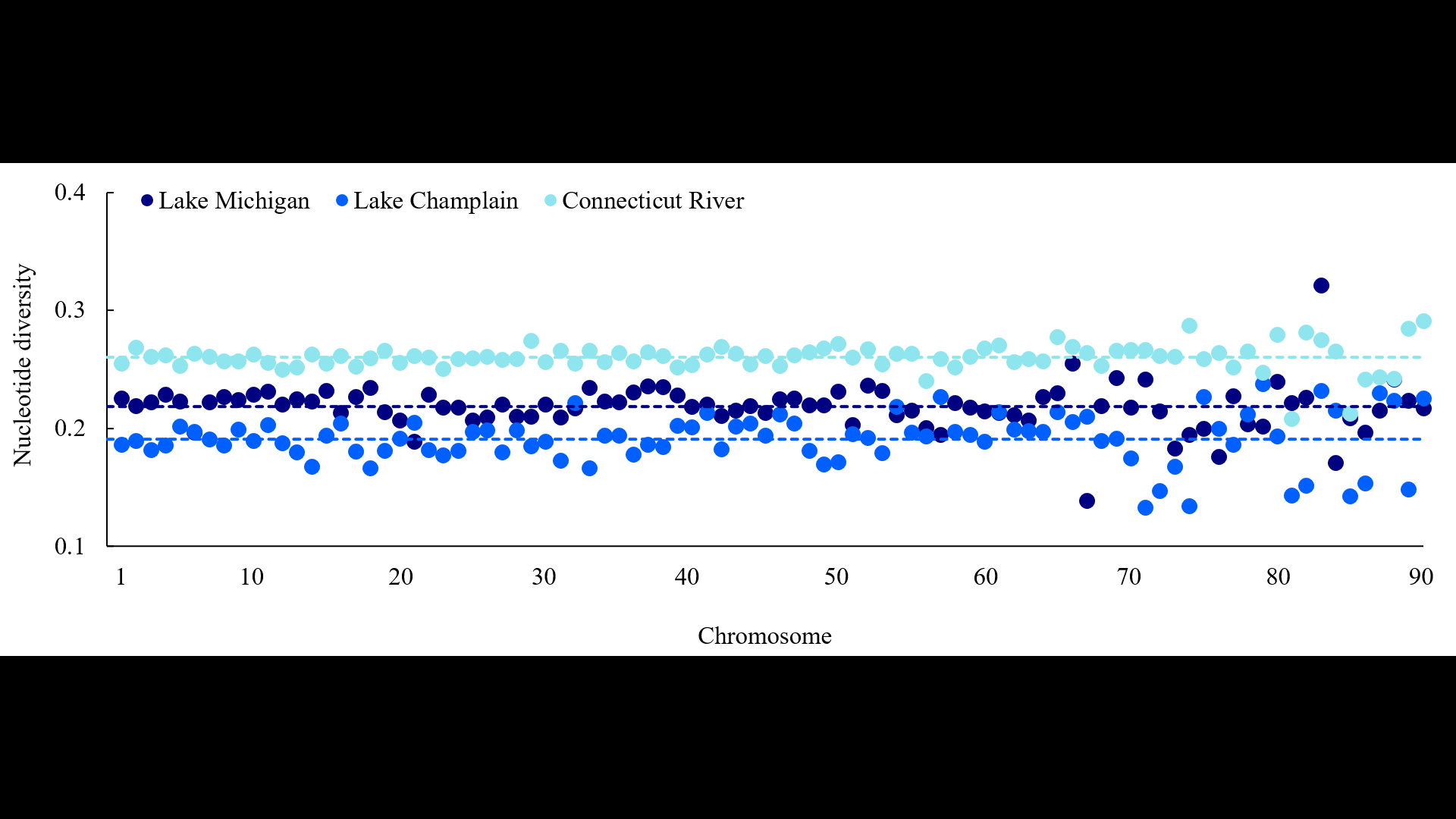


**Figure S3.** Observed heterozygosity and nucleotide diversity ($\pi$) of three populations. Sea lamprey have 99 chromosomes, of which the first 90 are assembled. Mean observed heterozygosity and mean nucleotide diversity across each chromosome are highly correlated in Lake Michigan (a), Lake Champlain (b), and Connecticut River (c) populations. Mean nucleotide diversity across the genome is indicated by dashed lines and nucleotide diversity of chromosomes is indicated by points (d). Nucleotide diversity ($\pi$) shows a similar pattern as observed heterozygosity, which is the highest in Connecticut River, followed by Lake Michigan and Lake Champlain (d).

(a) Lake Michigan vs. Lake Champlain

Z(*F_ST_*)

1 5 10 20 30 40 50 90

Chromosome


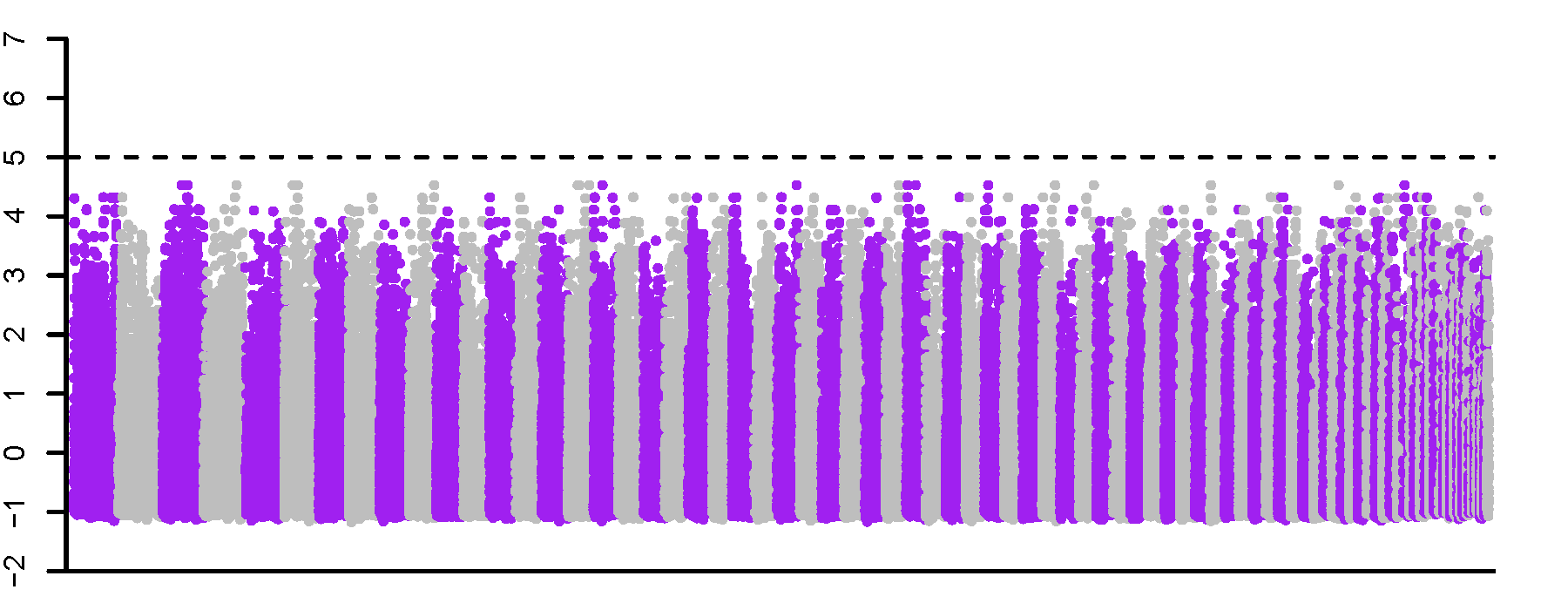


(b) Lake Michigan vs. Connecticut River


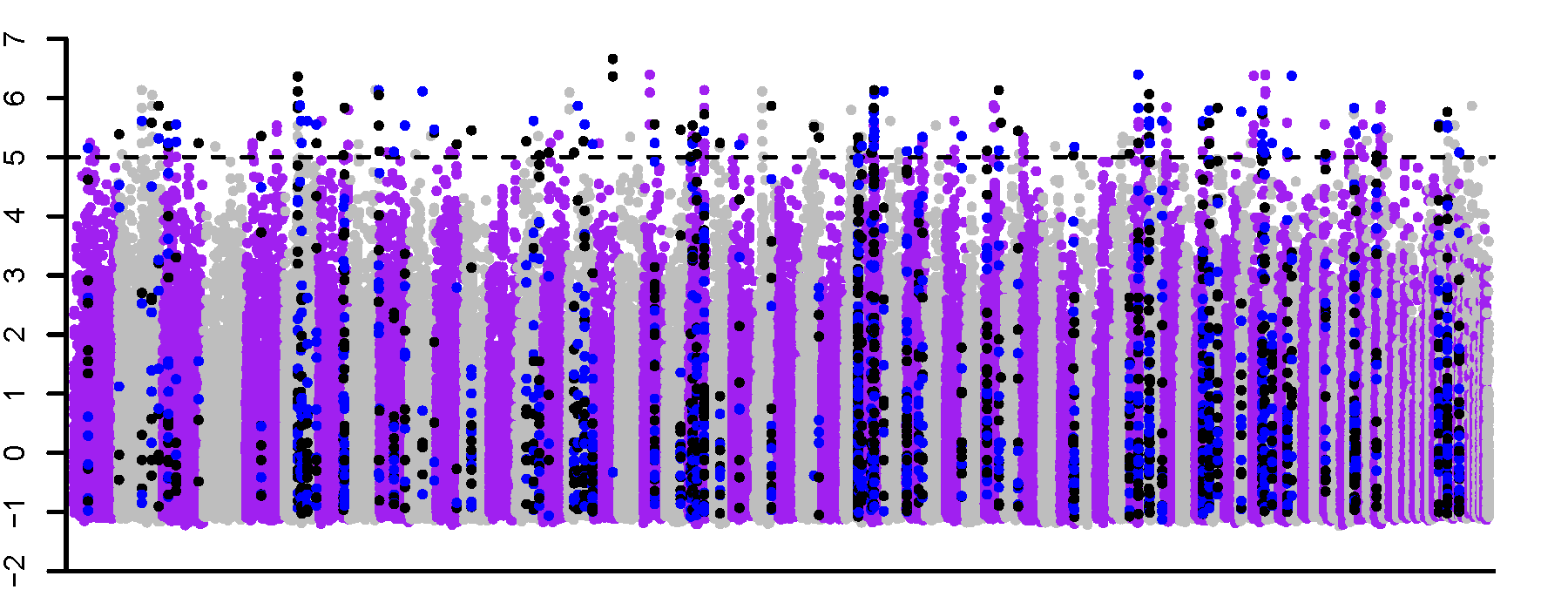


Z(*F_ST_*)

1 5 10 20 30 40 50 90

Chromosome

(c) Lake Champlain vs. Connecticut River


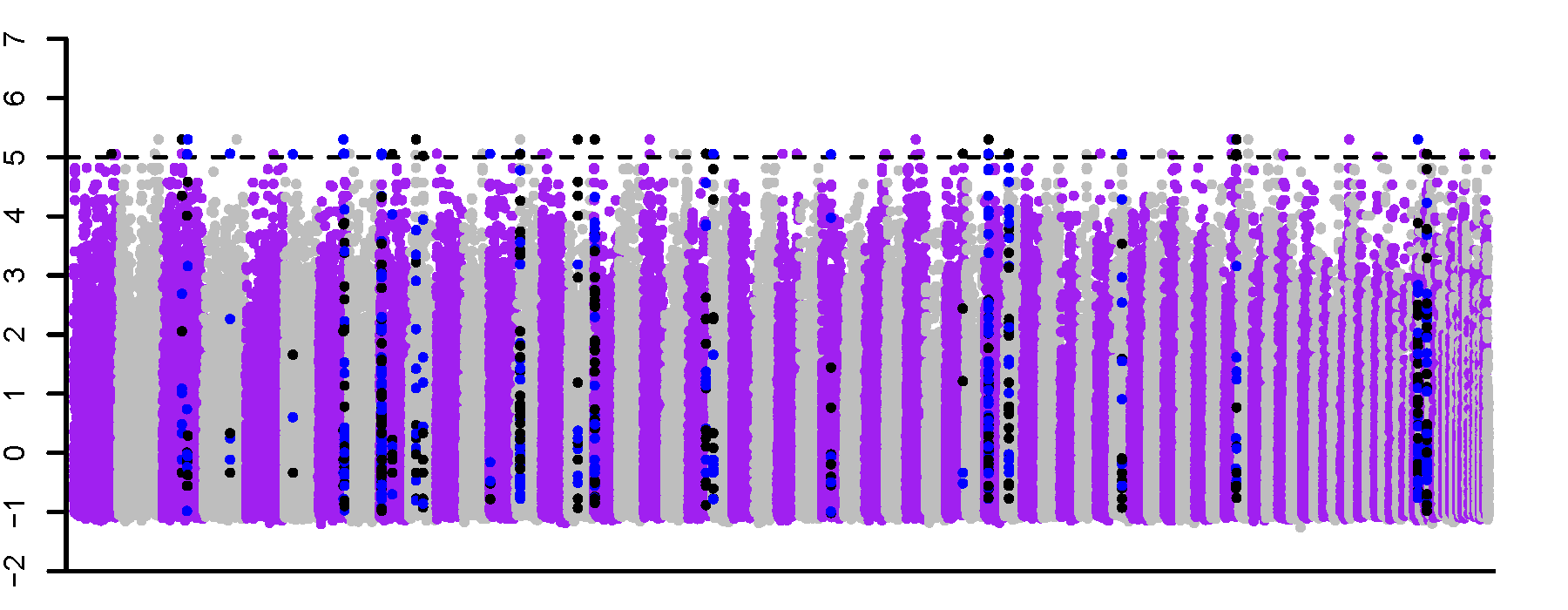


Z(*F_ST_*)

1 5 10 20 30 40 50 90

Chromosome

**Figure S4.** Z(*F_ST_*) along 90 chromosomes in three pairwise comparisons. Sea lamprey have 99 chromosomes, 90 of which are assembled. In comparison to Fig. 2 (a) & (b), this figure shows Z(*F_ST_*) of all SNPs in comparisons between Lake Michigan and Lake Champlain (a), Lake Michigan and Connecticut River (b), and Lake Champlain and Connecticut River (c), rather than mean Z(*F_ST_*) (Fig. 2 main text) of SNPs on genes without outlier SNPs. Alternating colors purple and grey represent Z(*F_ST_*) of SNPs on different chromosomes and alternating colors blue and black represent Z(*F_ST_*) of SNPs on neighboring outlier genes. Outlier SNPs with Z(*F_ST_*) but in purple and grey are those that are not located on any annotated gene.

(a)

(b)

**Figure S5.** Mean read depth of each population and number of shared loci in each pairwise comparison. Mean read depths in three populations are almost identical (a) and numbers of shared loci used to calculate *F_ST_* and *AFD* for three pairwise comparisons among Lake Michigan (LM), Lake Champlain (LC), and Connecticut River (CT) are almost identical (b).

(a) Lake Michigan vs. Lake Champlain


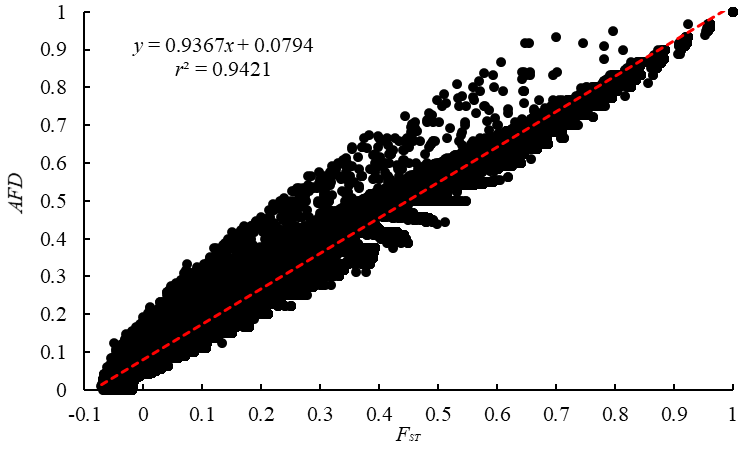


(b) Lake Michigan vs. Connecticut River


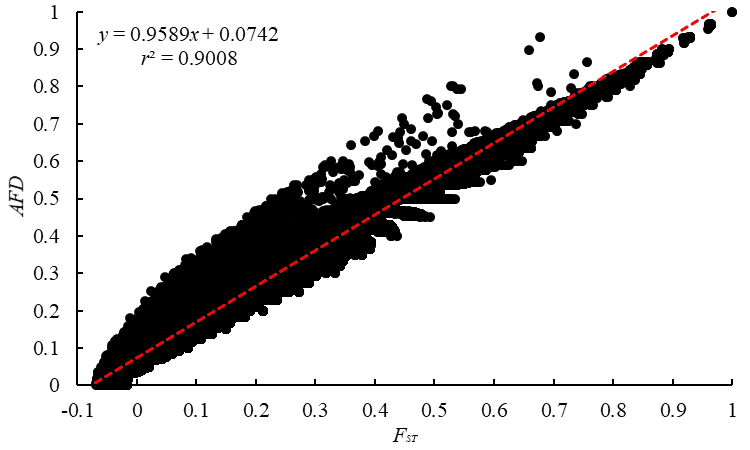


(c) Lake Champlain vs. Connecticut River


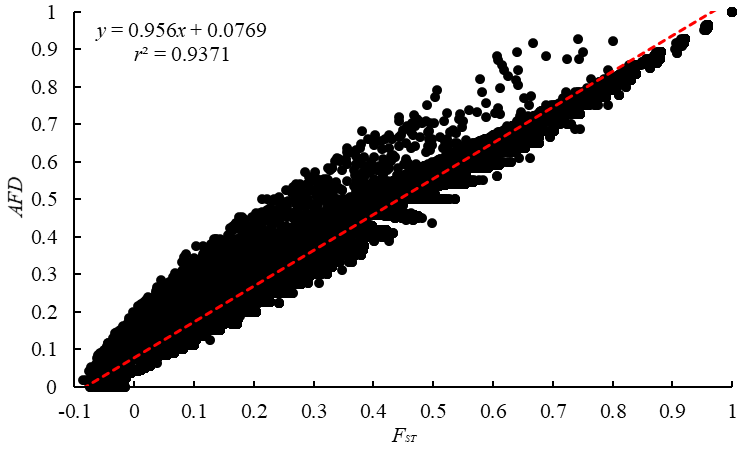


**Figure S6.** Correlations between *F_ST_* and *AFD* in three pairwise comparisons.

(a) Lake Michigan vs. Connecticut River


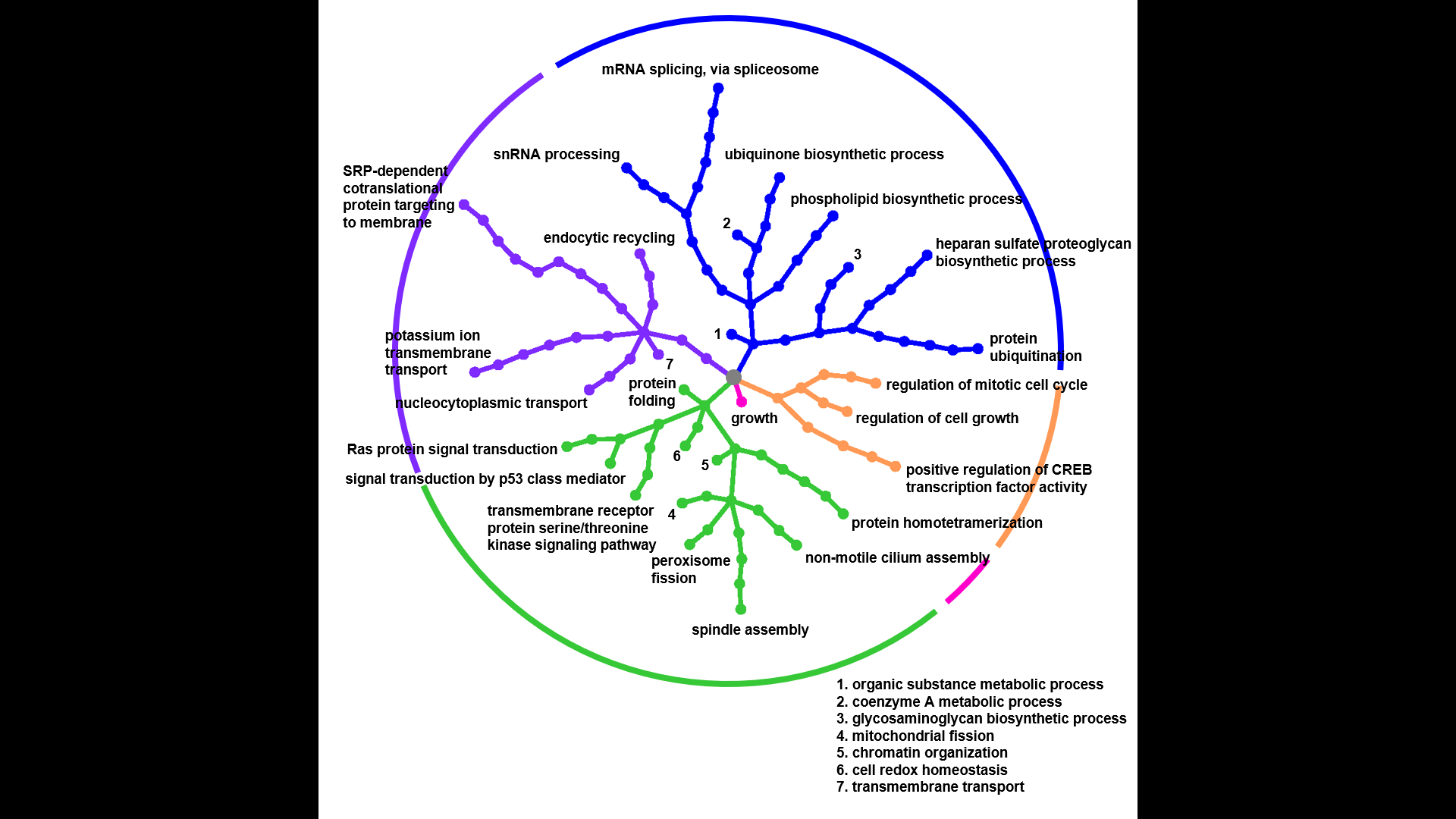


(b) Lake Champlain vs. Connecticut River


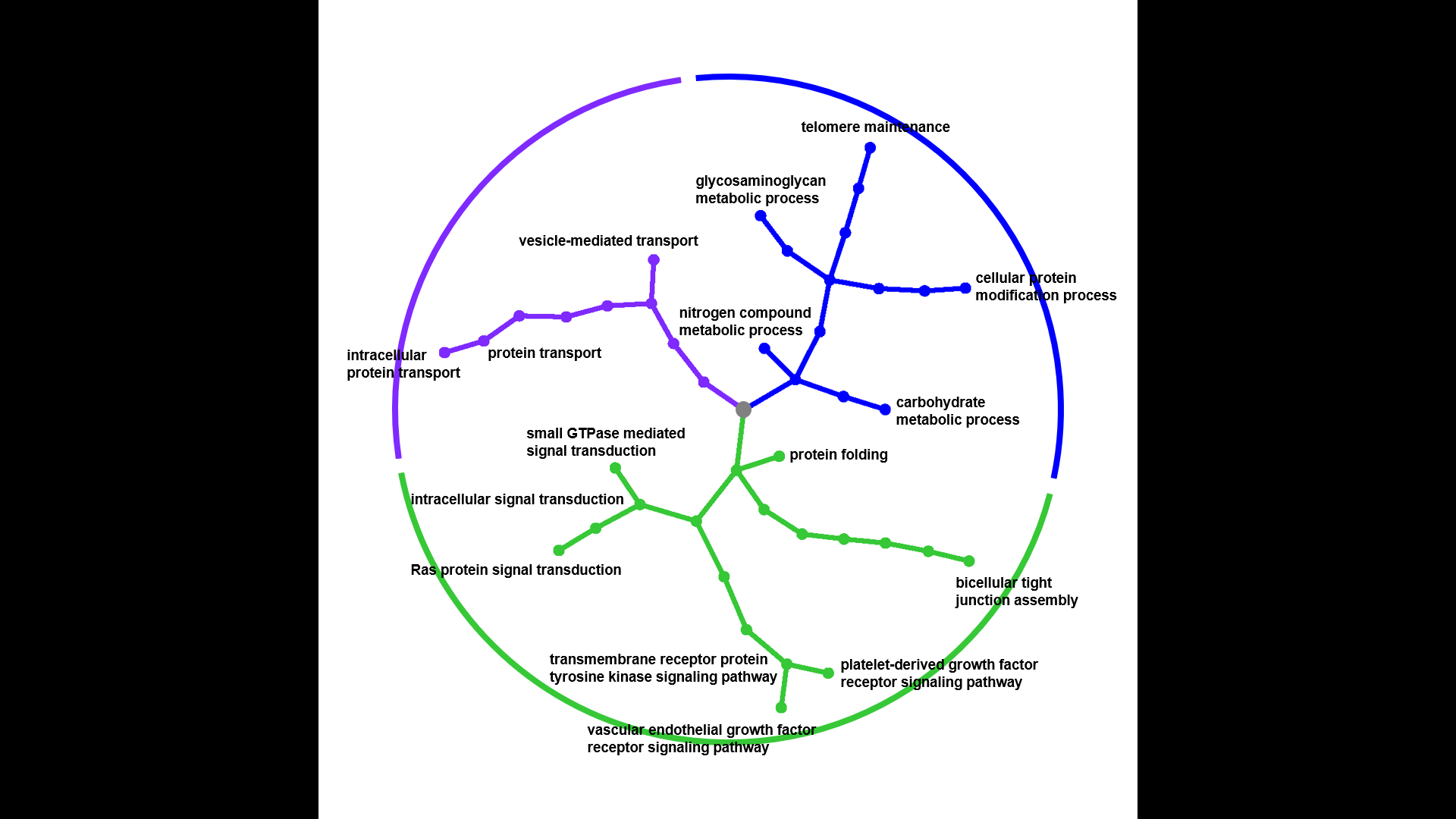


⚫ metabolic process ⚫ cellular process ⚫ biological regulation ⚫ localization ⚫ growth

**Figure S7.** Gene ontology (GO) hierarchy networks. The GO hierarchy networks are constructed from GO terms associated with one outlier gene in comparisons between Lake Michigan and Connecticut River (a) and between Lake Champlain and Connecticut River (b) using the *metacoder* package in R. Branch and node colors indicate the biological process child term to which distal nodes belong and the central grey node represents the biological process level of the GO hierarchy.

(a)


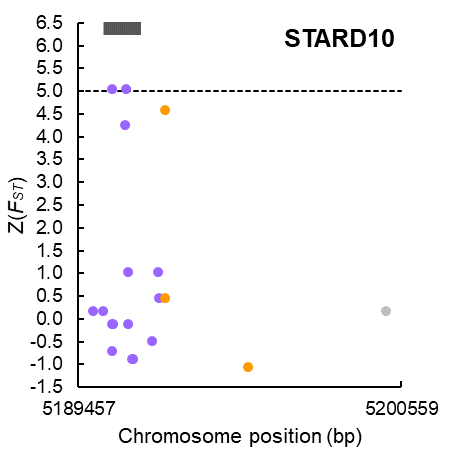

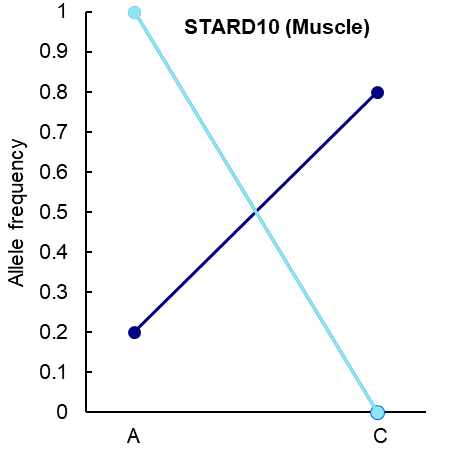

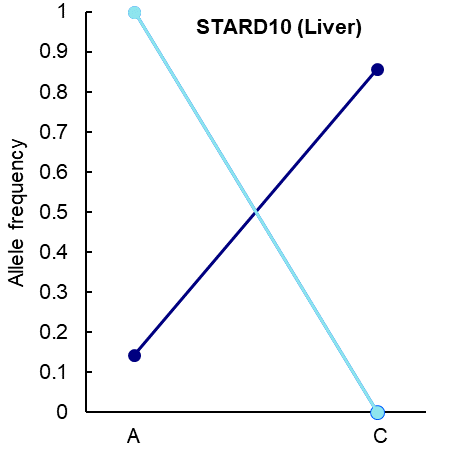


(b)


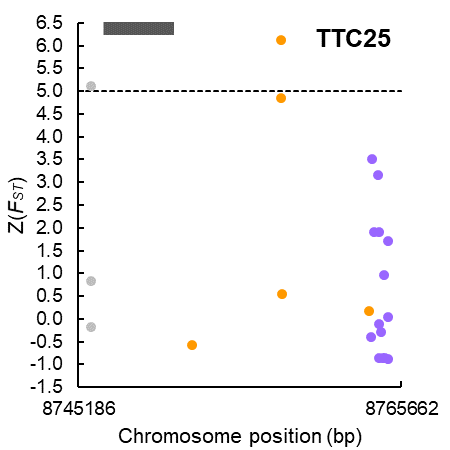

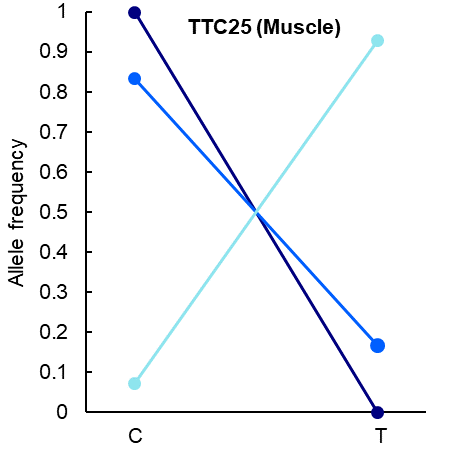

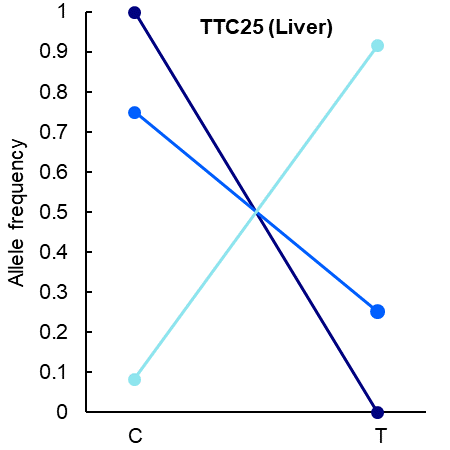


(c)


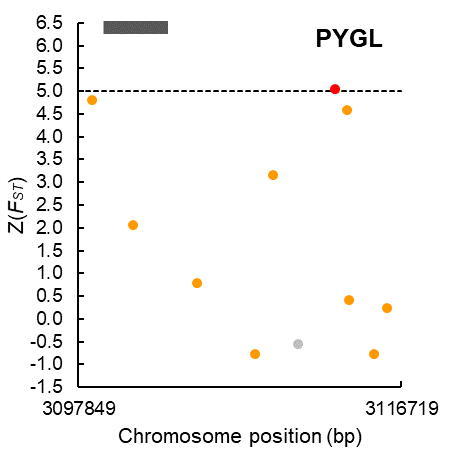

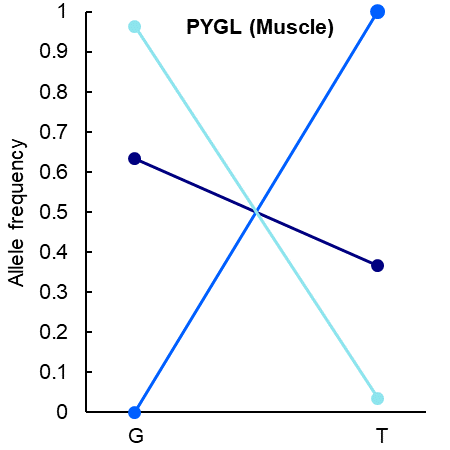

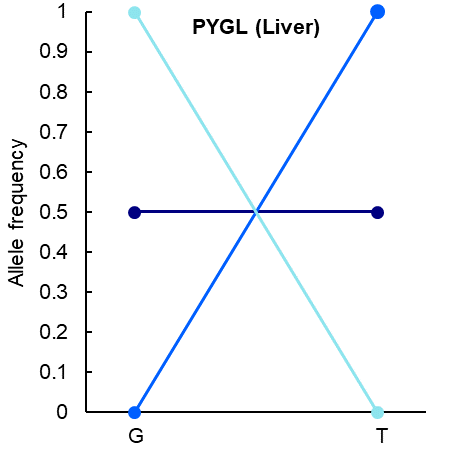


(d)


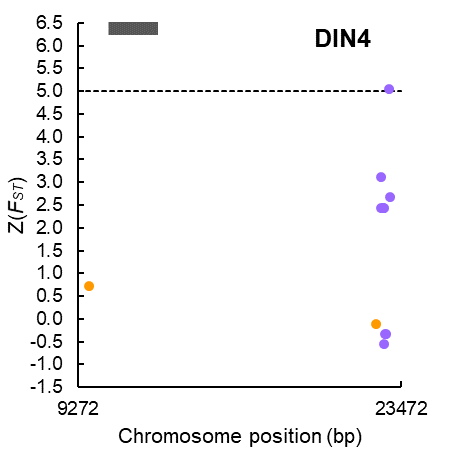

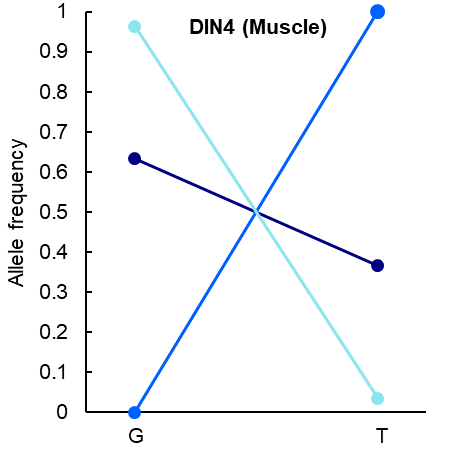

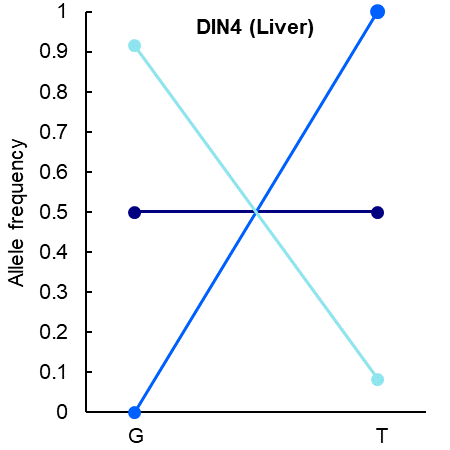


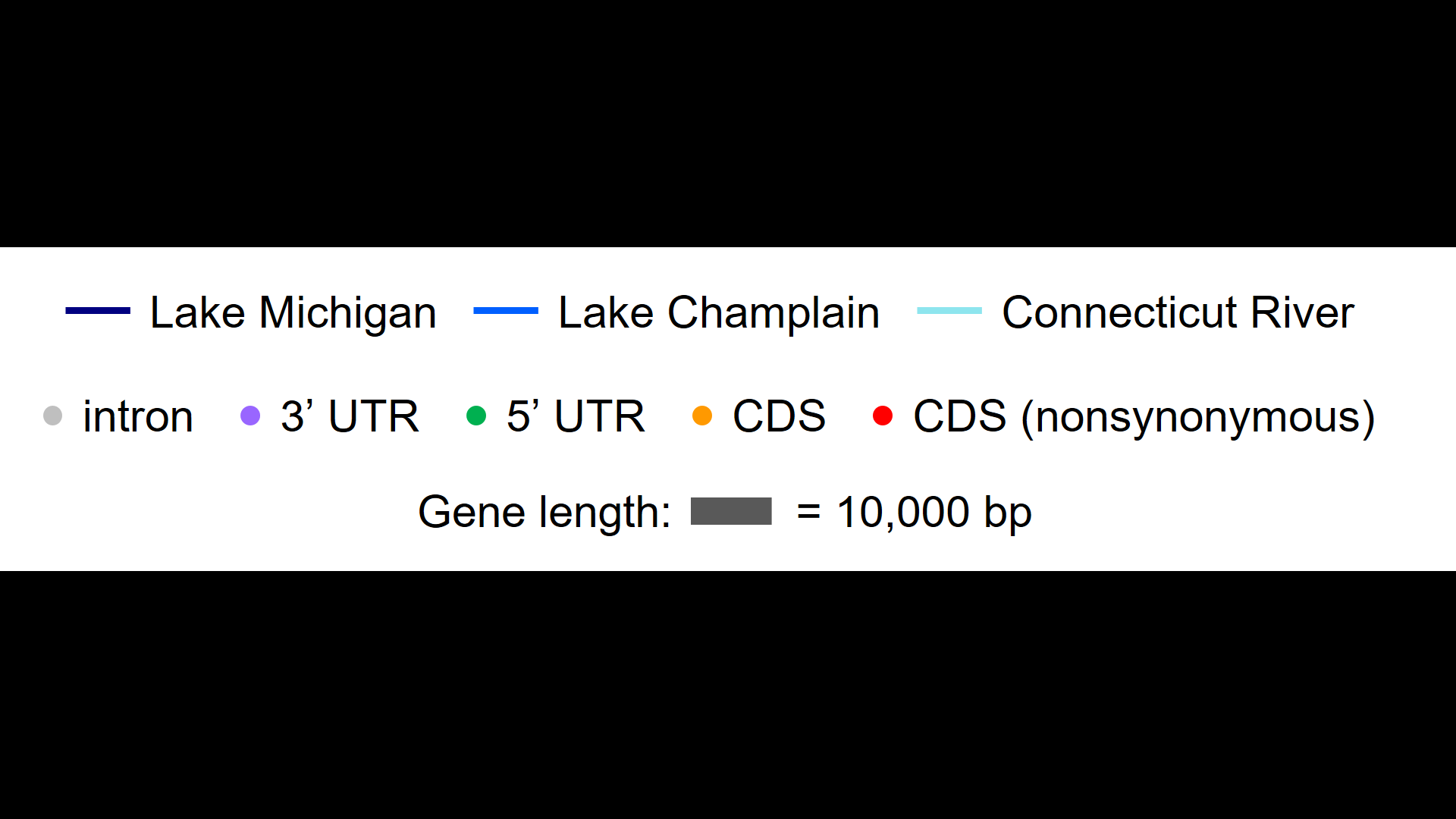


**Figure S8.** Z-transformed *F_ST_* (*i.e.,* Z(*F_ST_*)), genomic regions, and allele frequency of SNPs for additional outlier genes. Z(*F_ST_*) of all SNPs on outlier genes identified with muscle samples that may contribute to adaptation to local environments (*i.e.,* reproduction, bioenergetics) in sea lamprey are shown along corresponding chromosomes with SNPs on introns, untranslated regions (UTR), and coding regions (CDS) indicated in different colors (left panels). Shifts in allele frequency of SNPs with the highest Z(*F_ST_*) or causing nonsynonymous mutations on outlier genes in Lake Michigan, Lake Champlain, and Connecticut River populations are almost identical across different tissue types (*i.e.,* muscle and liver shown in middle and right panels, respectively). The dark grey horizontal bar on the top left of the left panel represents the length of an outlier gene. *STARD10* and *TTC25* are associated with reproduction (a, b), and *PYGL* and *DIN4* with bioenergetics (c, d).

*F_ST_* Nucleotide diversity within populations Nucleotide diversity between populations

**Figure S9**. *F_ST_*, nucleotide diversity within populations, and nucleotide diversity between populations at outlier and surrounding SNPs. Dashed lines represent average *F_ST_* (black lines), nucleotide diversity within populations (blue lines), and nucleotide diversity between populations (red lines) of all SNPs 50 Kb upstream and downstream (*i.e.,* 100 Kb window) from the outlier SNP with the highest *F_ST_*. Points indicate *F_ST_* (black), nucleotide diversity within populations (blue), and nucleotide diversity between populations (red) of outlier SNPs. Plots were qualitatively and nearly numerically identical for ±10 Kb, ±100 Kb, and ±500 Kb windows (with the exception of *PGR* at the ±10 Kb and ±500 Kb windows, which had slightly higher within population nucleotide diversity than the ±10 Kb and ±500 Kb averages). Single window averages were used instead of a standard sliding window approach because RNA-seq data generates SNP loci that are highly clustered within, but not between, genic regions. Where necessary, points were jittered for visual clarity. At all eight outlier genes, *F_ST_* and nucleotide diversity between populations tend to increase while nucleotide diversity within populations tends to decrease at outlier SNPs in comparison to the average, suggesting that divergence at these genes is driven by local adaptation.

**Table S1.** Summary of tissue samples used for RNA-seq.

| **Sample No.** | **ID** | **Experiment** | **Date** | **Population** | **Tissue type** | **TFM []** | **Sequencing machine** | **# mapped reads** | **Length (mm)** | **Weight (g)** |
| --- | --- | --- | --- | --- | --- | --- | --- | --- | --- | --- |
| 402 | lmc_liv_1 | GE 1 | 11/21/2016 | Lake Michigan | liver | 0 | NovaSeq 6000 | 19802319 | 91 | 0.795 |
| 403 | lmc_liv_2 | GE 1 | 11/21/2016 | Lake Michigan | liver | 0 | NovaSeq 6000 | 23747347 | 90 | 0.806 |
| 409 | lmc_liv_3 | GE 1 | 11/21/2016 | Lake Michigan | liver | 0 | NovaSeq 6000 | 28566552 | 92 | 0.93 |
| 407 | lmt_liv_1 | GE 1 | 11/21/2016 | Lake Michigan | liver | 0.2 | NovaSeq 6000 | 28658889 | 85 | 0.755 |
| 419 | lmt_liv_2 | GE 1 | 11/21/2016 | Lake Michigan | liver | 0.2 | NovaSeq 6000 | 28876080 | 81 | 0.699 |
| 422 | lmt_liv_3 | GE 1 | 11/21/2016 | Lake Michigan | liver | 0.2 | NovaSeq 6000 | 25875166 | 116 | 1.921 |
| 427 | lmt_liv_4 | GE 1 | 11/21/2016 | Lake Michigan | liver | 0.2 | NovaSeq 6000 | 20142378 | 76 | 0.558 |
| 402 | lm_c_1 | GE 1 | 11/21/2016 | Lake Michigan | muscle | 0 | HiSeq 2500 | 11765624 | 91 | 0.795 |
| 403 | lm_c_2 | GE 1 | 11/21/2016 | Lake Michigan | muscle | 0 | HiSeq 2500 | 16989420 | 90 | 0.806 |
| 409 | lm_c_4 | GE 1 | 11/21/2016 | Lake Michigan | muscle | 0 | HiSeq 2500 | 12741348 | 92 | 0.93 |
| 415 | lm_c_5 | GE 1 | 11/21/2016 | Lake Michigan | muscle | 0 | NovaSeq 6000 | 30008269 | 94 | 1.073 |
| 407 | lm_tl_1 | GE 1 | 11/21/2016 | Lake Michigan | muscle | 0.2 | HiSeq 2500 | 13866543 | 85 | 0.755 |
| 419 | lm_tl_3 | GE 1 | 11/21/2016 | Lake Michigan | muscle | 0.2 | NovaSeq 6000 | 24738305 | 81 | 0.669 |
| 422 | lm_tl_4 | GE 1 | 11/21/2016 | Lake Michigan | muscle | 0.2 | NovaSeq 6000 | 28538823 | 116 | 1.921 |
| 427 | lm_tl_5 | GE 1 | 11/21/2016 | Lake Michigan | muscle | 0.2 | NovaSeq 6000 | 26030162 | 76 | 0.558 |
| 418 | lm_th_1 | GE 1 | 11/21/2016 | Lake Michigan | muscle | 0.3 | HiSeq 2500 | 10924994 | 88 | 0.784 |
| 423 | lm_th_2 | GE 1 | 11/21/2016 | Lake Michigan | muscle | 0.3 | HiSeq 2500 | 15576432 | 87 | 0.747 |
| 400 | lcc_liv_1 | GE 1 | 11/21/2016 | Lake Champlain | liver | 0 | NovaSeq 6000 | 28506290 | 86 | 0.902 |
| 401 | lcc_liv_2 | GE 1 | 11/21/2016 | Lake Champlain | liver | 0 | NovaSeq 6000 | 28588031 | 70 | 0.391 |
| 426 | lcc_liv_3 | GE 1 | 11/21/2016 | Lake Champlain | liver | 0 | NovaSeq 6000 | 30269008 | 81 | 0.624 |
| 404 | lct_liv_1 | GE 1 | 11/21/2016 | Lake Champlain | liver | 0.2 | NovaSeq 6000 | 29966156 | 64 | 0.315 |
| 410 | lct_liv_2 | GE 1 | 11/21/2016 | Lake Champlain | liver | 0.2 | NovaSeq 6000 | 30003410 | 78 | 0.543 |
| 417 | lct_liv_3 | GE 1 | 11/21/2016 | Lake Champlain | liver | 0.2 | NovaSeq 6000 | 21071509 | 78 | 0.571 |
| 400 | lc_c_1 | GE 1 | 11/21/2016 | Lake Champlain | muscle | 0 | HiSeq 2500 | 11489986 | 86 | 0.902 |
| 401 | lc_c_2 | GE 1 | 11/21/2016 | Lake Champlain | muscle | 0 | HiSeq 2500 | 4771684 | 70 | 0.391 |
| 404 | lc_tl_1 | GE 1 | 11/21/2016 | Lake Champlain | muscle | 0.2 | NovaSeq 6000 | 25174478 | 64 | 0.315 |
| 410 | lc_tl_2 | GE 1 | 11/21/2016 | Lake Champlain | muscle | 0.2 | NovaSeq 6000 | 30140422 | 78 | 0.543 |
| 417 | lc_tl_3 | GE 1 | 11/21/2016 | Lake Champlain | muscle | 0.2 | NovaSeq 6000 | 24695462 | 78 | 0.571 |
| 405 | lc_th_1 | GE 1 | 11/21/2016 | Lake Champlain | muscle | 0.3 | HiSeq 2500 | 11612986 | 99 | 1.188 |
| 420 | lc_th_3 | GE 1 | 11/21/2016 | Lake Champlain | muscle | 0.3 | HiSeq 2500 | 14592886 | 74 | 0.542 |
| 412 | ctc_liv_1 | GE 1 | 11/21/2016 | Connecticut River | liver | 0 | NovaSeq 6000 | 20933885 | 93 | 1.232 |
| 413 | ctc_liv_2 | GE 1 | 11/21/2016 | Connecticut River | liver | 0 | NovaSeq 6000 | 32367561 | 142 | 4.299 |
| 424 | ctc_liv_3 | GE 1 | 11/21/2016 | Connecticut River | liver | 0 | NovaSeq 6000 | 19769485 | 137 | 2.716 |
| 411 | ctt_liv_1 | GE 1 | 11/21/2016 | Connecticut River | liver | 0.2 | NovaSeq 6000 | 17750173 | 82 | 0.716 |
| 414 | ctt_liv_2 | GE 1 | 11/21/2016 | Connecticut River | liver | 0.2 | NovaSeq 6000 | 22848428 | 122 | 1.905 |
| 416 | ctt_liv_3 | GE 1 | 11/21/2016 | Connecticut River | liver | 0.2 | NovaSeq 6000 | 26290947 | 92 | 0.933 |
| 412 | ct_c_1 | GE 1 | 11/21/2016 | Connecticut River | muscle | 0 | HiSeq 2500 | 16566749 | 93 | 1.232 |
| 413 | ct_c_2 | GE 1 | 11/21/2016 | Connecticut River | muscle | 0 | HiSeq 2500 | 13439299 | 142 | 4.299 |
| 424 | ct_c_3 | GE 1 | 11/21/2016 | Connecticut River | muscle | 0 | HiSeq 2500 | 14310452 | 137 | 2.716 |
| 411 | ct_tl_1 | GE 1 | 11/21/2016 | Connecticut River | muscle | 0.2 | NovaSeq 6000 | 19107299 | 82 | 0.716 |
| 414 | ct_tl_2 | GE 1 | 11/21/2016 | Connecticut River | muscle | 0.2 | NovaSeq 6000 | 26711086 | 122 | 1.905 |
| 416 | ct_tl_3 | GE 1 | 11/21/2016 | Connecticut River | muscle | 0.2 | NovaSeq 6000 | 19192978 | 92 | 0.933 |
| 406 | ct_th_1 | GE 1 | 11/21/2016 | Connecticut River | muscle | 0.3 | HiSeq 2500 | 15215385 | 113 | 1.607 |
| 421 | ct_th_2 | GE 1 | 11/21/2016 | Connecticut River | muscle | 0.3 | HiSeq 2500 | 11272452 | 103 | 2.262 |
| 429 | ct_th_3 | GE 1 | 11/21/2016 | Connecticut River | muscle | 0.3 | HiSeq 2500 | 15014323 | 105 | 1.253 |
| 567 | lmc_mus_ge2_1 | GE 2 | 11/30/2016 | Lake Michigan | muscle | 0 | NovaSeq 6000 | 32472014 | 94 | 0.951 |
| 581 | lmc_mus_ge2_2 | GE 2 | 11/30/2016 | Lake Michigan | muscle | 0 | NovaSeq 6000 | 26929835 | 90 | 0.766 |
| 573 | lmt_mus_ge2_3 | GE 2 | 11/30/2016 | Lake Michigan | muscle | 0.2 | NovaSeq 6000 | 25405100 | 90 | 1.002 |
| 576 | lmt_mus_ge2_4 | GE 2 | 11/30/2016 | Lake Michigan | muscle | 0.2 | NovaSeq 6000 | 25323666 | 90 | 0.908 |
| 577 | lmt_mus_ge2_1 | GE 2 | 11/30/2016 | Lake Michigan | muscle | 0.3 | NovaSeq 6000 | 29819726 | 95 | 1.04 |
| 591 | lmt_mus_ge2_2 | GE 2 | 11/30/2016 | Lake Michigan | muscle | 0.3 | NovaSeq 6000 | 21902654 | 100 | 1.084 |
| 566 | lcc_mus_ge2_1 | GE 2 | 11/30/2016 | Lake Champlain | muscle | 0 | NovaSeq 6000 | 28348812 | 95 | 0.973 |
| 572 | lcc_mus_ge2_2 | GE 2 | 11/30/2016 | Lake Champlain | muscle | 0 | NovaSeq 6000 | 31274558 | 77 | 0.584 |
| 580 | lcc_mus_ge2_3 | GE 2 | 11/30/2016 | Lake Champlain | muscle | 0 | NovaSeq 6000 | 29105459 | 85 | 0.781 |
| 571 | lct_mus_ge2_1 | GE 2 | 11/30/2016 | Lake Champlain | muscle | 0.2 | NovaSeq 6000 | 32878565 | 72 | 0.429 |
| 587 | lct_mus_ge2_2 | GE 2 | 11/30/2016 | Lake Champlain | muscle | 0.2 | NovaSeq 6000 | 15326492 | 70 | 0.407 |
| 588 | lct_mus_ge2_3 | GE 2 | 11/30/2016 | Lake Champlain | muscle | 0.2 | NovaSeq 6000 | 22899739 | 72 | 0.582 |
| 565 | ctc_mus_ge2_1 | GE 2 | 11/30/2016 | Connecticut River | muscle | 0 | NovaSeq 6000 | 21423126 | 74 | 0.48 |
| 583 | ctc_mus_ge2_2 | GE 2 | 11/30/2016 | Connecticut River | muscle | 0 | NovaSeq 6000 | 22933267 | 85 | 0.811 |
| 569 | ctt_mus_ge2_1 | GE 2 | 11/30/2016 | Connecticut River | muscle | 0.2 | NovaSeq 6000 | 23906705 | 140 | 3.335 |
| 579 | ctt_mus_ge2_2 | GE 2 | 11/30/2016 | Connecticut River | muscle | 0.2 | NovaSeq 6000 | 23538391 | 112 | 1.619 |
| 582 | ctt_mus_ge2_3 | GE 2 | 11/30/2016 | Connecticut River | muscle | 0.2 | NovaSeq 6000 | 23770608 | 110 | 1.541 |

**Table S2.** Summary of outlier SNPs and outlier genes. LM vs. CT stands for the pairwise comparison between Lake Michigan and Connecticut River populations, and LC vs. CT stands for the pairwise comparison between Lake Champlain and Connecticut River populations. See Table S3 for full gene names.

| **Comparison** | **Outlier SNP** | ***F_ST_*** | ***AFD*** | **∆*F_ST_*** | **Outlier gene** | **Gene name** | **Genomic region** | **Mutation type** | **Gene function** |
| --- | --- | --- | --- | --- | --- | --- | --- | --- | --- |
| LM vs. CT | scaf_00001:10123112 | 0.808 | 0.829 | 0.762 |  |  |  |  |  |
| LM vs. CT | scaf_00001:13035158 | 0.790 | 0.808 | 0.744 |  |  |  |  |  |
| LM vs. CT | scaf_00001:13035178 | 0.780 | 0.797 | 0.734 |  |  |  |  |  |
| LM vs. CT | scaf_00001:8835621 | 0.796 | 0.810 | 0.751 | scaf_00001:8825998-8840167 | Phactr1 | CDS | synonymous |  |
| LM vs. CT | scaf_00002:14563973 | 0.848 | 0.862 | 0.796 | scaf_00002:14556732-14576335 |  | CDS |  |  |
| LM vs. CT | scaf_00002:14565057 | 0.807 | 0.826 | 0.756 | scaf_00002:14556732-14576335 |  | intron |  |  |
| LM vs. CT | scaf_00002:14565067 | 0.807 | 0.826 | 0.756 | scaf_00002:14556732-14576335 |  | intron |  |  |
| LM vs. CT | scaf_00002:14565970 | 0.929 | 0.933 | 0.878 | scaf_00002:14556732-14576335 |  | intron |  |  |
| LM vs. CT | scaf_00002:14566432 | 0.888 | 0.898 | 0.837 | scaf_00002:14556732-14576335 |  | intron |  |  |
| LM vs. CT | scaf_00002:14566441 | 0.888 | 0.898 | 0.837 | scaf_00002:14556732-14576335 |  | intron |  |  |
| LM vs. CT | scaf_00002:14566476 | 0.888 | 0.898 | 0.837 | scaf_00002:14556732-14576335 |  | intron |  |  |
| LM vs. CT | scaf_00002:14566554 | 0.929 | 0.933 | 0.878 | scaf_00002:14556732-14576335 |  | intron |  |  |
| LM vs. CT | scaf_00002:14566770 | 0.929 | 0.933 | 0.878 | scaf_00002:14556732-14576335 |  | intron |  |  |
| LM vs. CT | scaf_00002:14567310 | 0.929 | 0.933 | 0.878 | scaf_00002:14556732-14576335 |  | intron |  |  |
| LM vs. CT | scaf_00002:14567311 | 0.929 | 0.933 | 0.878 | scaf_00002:14556732-14576335 |  | intron |  |  |
| LM vs. CT | scaf_00002:14573745 | 0.807 | 0.826 | 0.756 | scaf_00002:14556732-14576335 |  | intron |  |  |
| LM vs. CT | scaf_00002:14574416 | 0.807 | 0.826 | 0.756 | scaf_00002:14556732-14576335 |  | 3' UTR |  |  |
| LM vs. CT | scaf_00002:14574676 | 0.929 | 0.933 | 0.878 | scaf_00002:14556732-14576335 |  | 3' UTR |  |  |
| LM vs. CT | scaf_00002:14594865 | 0.858 | 0.867 | 0.807 | scaf_00002:14590314-14616860 | St5 | CDS | nonsynonymous | tumor related |
| LM vs. CT | scaf_00002:19165741 | 0.812 | 0.824 | 0.760 |  |  |  |  |  |
| LM vs. CT | scaf_00002:20647794 | 0.854 | 0.857 | 0.803 | scaf_00002:20575854-20648365 | EXT2 |  | synonymous |  |
| LM vs. CT | scaf_00002:20657240 | 0.854 | 0.857 | 0.803 |  |  |  |  |  |
| LM vs. CT | scaf_00002:20965626 | 0.821 | 0.821 | 0.770 |  |  |  |  |  |
| LM vs. CT | scaf_00002:20968753 | 0.893 | 0.893 | 0.842 |  |  |  |  |  |
| LM vs. CT | scaf_00002:20970835 | 0.917 | 0.929 | 0.866 |  |  |  |  |  |
| LM vs. CT | scaf_00002:2345066 | 0.782 | 0.804 | 0.730 | scaf_00002:2312819-2345502 |  | 3' UTR |  |  |
| LM vs. CT | scaf_00002:25028301 | 0.818 | 0.821 | 0.767 | scaf_00002:24992371-25031642 | USP47 | CDS | nonsynonymous | DNA replication, repair, modification |
| LM vs. CT | scaf_00002:25028328 | 0.893 | 0.893 | 0.842 | scaf_00002:24992371-25031642 | USP47 | CDS | nonsynonymous | DNA replication, repair, modification |
| LM vs. CT | scaf_00002:492146 | 0.828 | 0.800 | 0.777 | scaf_00002:465212-496992 | CD109 | CDS | nonsynonymous | cellular signaling |
| LM vs. CT | scaf_00003:22285030 | 0.807 | 0.826 | 0.761 | scaf_00003:22273477-22292330 | SRPR |  | synonymous |  |
| LM vs. CT | scaf_00003:3514727 | 0.847 | 0.860 | 0.801 | scaf_00003:3513229-3515830 | USP28 |  | synonymous | DNA replication, repair, modification |
| LM vs. CT | scaf_00003:3516222 | 0.847 | 0.860 | 0.801 |  |  |  |  |  |
| LM vs. CT | scaf_00003:3518608 | 0.808 | 0.829 | 0.762 |  |  |  |  |  |
| LM vs. CT | scaf_00003:3546917 | 0.800 | 0.821 | 0.754 | scaf_00003:3544931-3562671 | TBC1D23 |  | synonymous |  |
| LM vs. CT | scaf_00003:3546930 | 0.808 | 0.829 | 0.762 | scaf_00003:3544931-3562671 | TBC1D23 |  | synonymous |  |
| LM vs. CT | scaf_00003:3588013 | 0.807 | 0.826 | 0.761 |  |  |  |  |  |
| LM vs. CT | scaf_00003:3588246 | 0.787 | 0.815 | 0.741 |  |  |  |  |  |
| LM vs. CT | scaf_00003:8368697 | 0.850 | 0.864 | 0.805 | scaf_00003:8356671-8370662 | v1g239264 | CDS | synonymous |  |
| LM vs. CT | scaf_00003:8429112 | 0.810 | 0.821 | 0.764 | scaf_00003:8421456-8430783 | NDUFA9 |  | synonymous |  |
| LM vs. CT | scaf_00004:7090450 | 0.800 | 0.821 | 0.754 |  |  |  |  |  |
| LM vs. CT | scaf_00004:7090451 | 0.800 | 0.821 | 0.754 |  |  |  |  |  |
| LM vs. CT | scaf_00005:19581917 | 0.782 | 0.800 | 0.737 |  |  |  |  |  |
| LM vs. CT | scaf_00005:19617660 | 0.848 | 0.864 | 0.802 |  |  |  |  |  |
| LM vs. CT | scaf_00005:19617665 | 0.848 | 0.864 | 0.802 |  |  |  |  |  |
| LM vs. CT | scaf_00005:19617674 | 0.848 | 0.864 | 0.802 |  |  |  |  |  |
| LM vs. CT | scaf_00005:19617933 | 0.834 | 0.849 | 0.788 |  |  |  |  |  |
| LM vs. CT | scaf_00005:4664149 | 0.807 | 0.826 | 0.761 |  |  |  |  |  |
| LM vs. CT | scaf_00005:4664167 | 0.807 | 0.826 | 0.761 |  |  |  |  |  |
| LM vs. CT | scaf_00005:4664325 | 0.807 | 0.826 | 0.761 |  |  |  |  |  |
| LM vs. CT | scaf_00005:4664346 | 0.807 | 0.826 | 0.761 |  |  |  |  |  |
| LM vs. CT | scaf_00005:4664432 | 0.799 | 0.818 | 0.753 |  |  |  |  |  |
| LM vs. CT | scaf_00005:4664619 | 0.780 | 0.797 | 0.734 |  |  |  |  |  |
| LM vs. CT | scaf_00005:4664625 | 0.780 | 0.797 | 0.734 |  |  |  |  |  |
| LM vs. CT | scaf_00005:9935496 | 0.823 | 0.833 | 0.778 | scaf_00005:9934375-9985347 | SLC4A10 |  | synonymous | osmoregulation? |
| LM vs. CT | scaf_00005:9936080 | 0.823 | 0.833 | 0.778 | scaf_00005:9934375-9985347 | SLC4A10 |  | synonymous | osmoregulation? |
| LM vs. CT | scaf_00006:10163362 | 0.858 | 0.867 | 0.783 | scaf_00006:10144504-10166450 | crtc3 | CDS | synonymous |  |
| LM vs. CT | scaf_00006:10617528 | 0.808 | 0.829 | 0.732 | scaf_00006:10559654-10633178 | TJP2 | CDS | nonsynonymous | cellular component assembly |
| LM vs. CT | scaf_00006:14101018 | 0.858 | 0.867 | 0.783 | scaf_00006:14080605-14105391 | HEXB |  | synonymous | enzyme |
| LM vs. CT | scaf_00006:14101308 | 0.858 | 0.867 | 0.783 | scaf_00006:14080605-14105391 | HEXB |  | synonymous | enzyme |
| LM vs. CT | scaf_00006:14115685 | 0.858 | 0.867 | 0.783 | scaf_00006:14115645-14120297 |  | CDS |  |  |
| LM vs. CT | scaf_00006:19580720 | 0.850 | 0.860 | 0.774 |  |  |  |  |  |
| LM vs. CT | scaf_00006:19755442 | 0.807 | 0.826 | 0.732 | scaf_00006:19754899-19759825 | NMRK2 | CDS | nonsynonymous | tissue development |
| LM vs. CT | scaf_00006:19770269 | 0.850 | 0.860 | 0.774 | scaf_00006:19761580-19772377 | Ostf1 | CDS | synonymous | tissue development |
| LM vs. CT | scaf_00006:8062777 | 0.823 | 0.833 | 0.748 |  |  |  |  |  |
| LM vs. CT | scaf_00006:8116093 | 0.888 | 0.895 | 0.813 |  |  |  |  |  |
| LM vs. CT | scaf_00006:8116354 | 0.848 | 0.862 | 0.772 |  |  |  |  |  |
| LM vs. CT | scaf_00006:8161123 | 0.888 | 0.898 | 0.813 | scaf_00006:8161015-8178340 | mybB | CDS | nonsynonymous | cell cycle progression |
| LM vs. CT | scaf_00006:8180887 | 0.960 | 0.964 | 0.885 | scaf_00006:8180831-8190233 | RNASEH2A |  | synonymous |  |
| LM vs. CT | scaf_00006:8194922 | 0.926 | 0.931 | 0.851 | scaf_00006:8194338-8203385 | Alg6 | CDS | synonymous |  |
| LM vs. CT | scaf_00006:8295307 | 0.888 | 0.898 | 0.813 |  |  |  |  |  |
| LM vs. CT | scaf_00006:8300740 | 0.893 | 0.893 | 0.818 | scaf_00006:8297159-8321513 | SZT2 | CDS | nonsynonymous | stress response |
| LM vs. CT | scaf_00006:8333282 | 0.848 | 0.862 | 0.772 |  |  |  |  |  |
| LM vs. CT | scaf_00006:8333381 | 0.848 | 0.862 | 0.772 |  |  |  |  |  |
| LM vs. CT | scaf_00006:9402357 | 0.812 | 0.824 | 0.736 |  |  |  |  |  |
| LM vs. CT | scaf_00006:9615782 | 0.894 | 0.900 | 0.818 | scaf_00006:9614947-9645876 | AP1M1 |  | synonymous |  |
| LM vs. CT | scaf_00007:15892551 | 0.780 | 0.786 | 0.730 | scaf_00007:15885964-15901262 | Lztr1 |  | synonymous | cellular transporting |
| LM vs. CT | scaf_00007:16498424 | 0.888 | 0.898 | 0.838 | scaf_00007:16454955-16500014 | Cltc |  | synonymous | cellular transporting |
| LM vs. CT | scaf_00007:18773916 | 0.883 | 0.890 | 0.834 |  |  |  |  |  |
| LM vs. CT | scaf_00007:18773933 | 0.803 | 0.813 | 0.754 |  |  |  |  |  |
| LM vs. CT | scaf_00007:18773934 | 0.803 | 0.813 | 0.754 |  |  |  |  |  |
| LM vs. CT | scaf_00007:2165885 | 0.858 | 0.867 | 0.809 |  |  |  |  |  |
| LM vs. CT | scaf_00007:2165914 | 0.858 | 0.867 | 0.809 |  |  |  |  |  |
| LM vs. CT | scaf_00008:16328496 | 0.929 | 0.933 | 0.883 |  |  |  |  |  |
| LM vs. CT | scaf_00008:18393303 | 0.929 | 0.933 | 0.883 | scaf_00008:18383265-18399211 | Sf3b3 | CDS | synonymous |  |
| LM vs. CT | scaf_00008:18397644 | 0.917 | 0.929 | 0.872 | scaf_00008:18383265-18399211 | Sf3b3 |  | synonymous |  |
| LM vs. CT | scaf_00008:18486615 | 0.848 | 0.862 | 0.802 | scaf_00008:18478285-18486785 | DDB_G0271982 | CDS | synonymous |  |
| LM vs. CT | scaf_00008:18856425 | 0.789 | 0.800 | 0.743 | scaf_00008:18855513-18873107 | dye |  | synonymous |  |
| LM vs. CT | scaf_00008:18856426 | 0.789 | 0.800 | 0.743 | scaf_00008:18855513-18873107 | dye |  | synonymous |  |
| LM vs. CT | scaf_00008:5895458 | 0.805 | 0.792 | 0.759 |  |  |  |  |  |
| LM vs. CT | scaf_00008:5895532 | 0.805 | 0.792 | 0.759 |  |  |  |  |  |
| LM vs. CT | scaf_00009:15652724 | 0.848 | 0.862 | 0.802 | scaf_00009:15640027-15652962 | KCNK1 |  | synonymous |  |
| LM vs. CT | scaf_00009:4635121 | 0.786 | 0.800 | 0.740 |  |  |  |  |  |
| LM vs. CT | scaf_00009:4635615 | 0.786 | 0.800 | 0.740 |  |  |  |  |  |
| LM vs. CT | scaf_00009:8930488 | 0.788 | 0.813 | 0.742 | scaf_00009:8928984-8938824 | coq6 |  | synonymous | energy supply |
| LM vs. CT | scaf_00010:13783349 | 0.823 | 0.833 | 0.781 |  |  |  |  |  |
| LM vs. CT | scaf_00010:13784701 | 0.823 | 0.833 | 0.781 |  |  |  |  |  |
| LM vs. CT | scaf_00010:15965099 | 0.831 | 0.846 | 0.789 | scaf_00010:15949556-15968056 | Cdkl5 |  | synonymous |  |
| LM vs. CT | scaf_00010:15965129 | 0.838 | 0.854 | 0.796 | scaf_00010:15949556-15968056 | Cdkl5 |  | synonymous |  |
| LM vs. CT | scaf_00010:4383634 | 0.782 | 0.804 | 0.739 |  |  |  |  |  |
| LM vs. CT | scaf_00010:8722195 | 0.926 | 0.929 | 0.884 | scaf_00010:8711285-8726242 | hspa14 | CDS | synonymous |  |
| LM vs. CT | scaf_00011:11073373 | 0.783 | 0.800 | 0.741 |  |  |  |  |  |
| LM vs. CT | scaf_00011:12459380 | 0.805 | 0.792 | 0.763 | scaf_00011:12449551-12463962 | MAP2K3 | CDS | synonymous |  |
| LM vs. CT | scaf_00011:8627947 | 0.788 | 0.773 | 0.746 | scaf_00011:8603674-8648571 |  | CDS |  |  |
| LM vs. CT | scaf_00012:5196850 | 0.837 | 0.818 | 0.791 | scaf_00012:5180339-5205682 | Cdh23 | CDS | synonymous |  |
| LM vs. CT | scaf_00014:10986666 | 0.858 | 0.867 | 0.813 | scaf_00014:10974573-10988806 | MOB2 | CDS | synonymous | kinase activator |
| LM vs. CT | scaf_00014:13888053 | 0.823 | 0.833 | 0.778 |  |  |  |  |  |
| LM vs. CT | scaf_00014:14543750 | 0.780 | 0.786 | 0.734 | scaf_00014:14502262-14544458 | St5 |  | synonymous | tumor related |
| LM vs. CT | scaf_00014:6529657 | 0.812 | 0.824 | 0.766 | scaf_00014:6528290-6558952 | OSBPL5 |  | synonymous |  |
| LM vs. CT | scaf_00015:10243038 | 0.826 | 0.839 | 0.780 |  |  |  |  |  |
| LM vs. CT | scaf_00015:4219456 | 0.786 | 0.800 | 0.740 | scaf_00015:4219341-4228598 | Morn4 |  | synonymous |  |
| LM vs. CT | scaf_00015:4219567 | 0.786 | 0.800 | 0.740 | scaf_00015:4219341-4228598 | Morn4 |  | synonymous |  |
| LM vs. CT | scaf_00015:4219599 | 0.786 | 0.800 | 0.740 | scaf_00015:4219341-4228598 | Morn4 |  | synonymous |  |
| LM vs. CT | scaf_00015:5332998 | 0.808 | 0.829 | 0.762 |  |  |  |  |  |
| LM vs. CT | scaf_00016:10271281 | 0.850 | 0.864 | 0.805 | scaf_00016:10261998-10279456 | dthadh |  | synonymous | enzyme |
| LM vs. CT | scaf_00016:15164267 | 0.805 | 0.821 | 0.759 | scaf_00016:15152461-15165657 | ACVR1 |  | synonymous | cellular signaling |
| LM vs. CT | scaf_00016:3703083 | 0.786 | 0.800 | 0.740 | scaf_00016:3701654-3717437 | Pofut2 | CDS | nonsynonymous | enzyme |
| LM vs. CT | scaf_00016:6048289 | 0.893 | 0.893 | 0.847 | scaf_00016:6041862-6050713 | Rala |  | synonymous | cellular signaling |
| LM vs. CT | scaf_00016:791476 | 0.885 | 0.885 | 0.839 |  |  |  |  |  |
| LM vs. CT | scaf_00016:791494 | 0.885 | 0.893 | 0.840 |  |  |  |  |  |
| LM vs. CT | scaf_00016:791512 | 0.923 | 0.929 | 0.878 |  |  |  |  |  |
| LM vs. CT | scaf_00016:9521876 | 0.812 | 0.824 | 0.766 | scaf_00016:9519718-9526159 | Phb | CDS | synonymous |  |
| LM vs. CT | scaf_00017:11887123 | 1.000 | 1.000 | 0.966 | scaf_00017:11872290-11888569 | VSNL1 |  | synonymous |  |
| LM vs. CT | scaf_00017:11887137 | 0.960 | 0.967 | 0.926 | scaf_00017:11872290-11888569 | VSNL1 |  | synonymous |  |
| LM vs. CT | scaf_00017:11887144 | 0.960 | 0.967 | 0.926 | scaf_00017:11872290-11888569 | VSNL1 |  | synonymous |  |
| LM vs. CT | scaf_00017:3214803 | 0.807 | 0.826 | 0.773 |  |  |  |  |  |
| LM vs. CT | scaf_00018:7300566 | 0.821 | 0.821 | 0.776 |  |  |  |  |  |
| LM vs. CT | scaf_00019:3935062 | 0.964 | 0.967 | 0.918 |  |  |  |  |  |
| LM vs. CT | scaf_00019:3979942 | 0.850 | 0.860 | 0.804 | scaf_00019:3957040-3986041 |  | intron |  |  |
| LM vs. CT | scaf_00019:3983067 | 0.923 | 0.928 | 0.877 | scaf_00019:3957040-3986041 |  | CDS |  |  |
| LM vs. CT | scaf_00019:6987315 | 0.808 | 0.829 | 0.762 | scaf_00019:6966780-6989673 | Tmc7 |  | synonymous | cellular transporting |
| LM vs. CT | scaf_00019:6987396 | 0.850 | 0.864 | 0.805 | scaf_00019:6966780-6989673 | Tmc7 |  | synonymous | cellular transporting |
| LM vs. CT | scaf_00019:6987652 | 0.850 | 0.864 | 0.805 | scaf_00019:6966780-6989673 | Tmc7 |  | synonymous | cellular transporting |
| LM vs. CT | scaf_00020:9712277 | 0.838 | 0.849 | 0.792 | scaf_00020:9706189-9731976 | oplah | CDS | synonymous |  |
| LM vs. CT | scaf_00021:1461693 | 0.776 | 0.786 | 0.726 | scaf_00021:1460415-1494086 | Aff3 |  | synonymous |  |
| LM vs. CT | scaf_00021:2633915 | 0.813 | 0.831 | 0.763 | scaf_00021:2627703-2637761 | ucp2 | CDS | synonymous | energy supply |
| LM vs. CT | scaf_00021:2636147 | 0.806 | 0.828 | 0.757 | scaf_00021:2627703-2637761 | ucp2 |  | synonymous | energy supply |
| LM vs. CT | scaf_00021:2636264 | 0.848 | 0.857 | 0.798 | scaf_00021:2627703-2637761 | ucp2 |  | synonymous | energy supply |
| LM vs. CT | scaf_00021:2730971 | 0.823 | 0.833 | 0.774 | scaf_00021:2725081-2731213 |  | intron |  |  |
| LM vs. CT | scaf_00021:2731427 | 0.823 | 0.833 | 0.774 |  |  |  |  |  |
| LM vs. CT | scaf_00021:2735013 | 0.812 | 0.833 | 0.763 |  |  |  |  |  |
| LM vs. CT | scaf_00021:2735470 | 0.823 | 0.833 | 0.774 |  |  |  |  |  |
| LM vs. CT | scaf_00021:5075765 | 0.820 | 0.833 | 0.771 | scaf_00021:5071783-5084898 | ARAP2 | CDS | synonymous | cellular signaling |
| LM vs. CT | scaf_00021:5190635 | 0.782 | 0.800 | 0.733 | scaf_00021:5189847-5208509 | Stard10 |  | synonymous | reproduction |
| LM vs. CT | scaf_00021:5191091 | 0.782 | 0.800 | 0.733 | scaf_00021:5189847-5208509 | Stard10 |  | synonymous | reproduction |
| LM vs. CT | scaf_00021:5220596 | 0.782 | 0.800 | 0.733 | scaf_00021:5215702-5268693 | RELL1 |  | synonymous | cellular signaling |
| LM vs. CT | scaf_00021:5278795 | 0.810 | 0.831 | 0.760 |  |  |  |  |  |
| LM vs. CT | scaf_00021:5278820 | 0.810 | 0.831 | 0.760 |  |  |  |  |  |
| LM vs. CT | scaf_00021:5278832 | 0.810 | 0.831 | 0.760 |  |  |  |  |  |
| LM vs. CT | scaf_00021:9722010 | 0.835 | 0.852 | 0.785 | scaf_00021:9718637-9730460 | ACRC |  | synonymous | tumor related |
| LM vs. CT | scaf_00021:9883404 | 0.874 | 0.881 | 0.824 | scaf_00021:9877432-9891556 | SLC25A15 |  | synonymous | ornithine mitochondrial transporter |
| LM vs. CT | scaf_00021:9892037 | 0.783 | 0.786 | 0.733 |  |  |  |  |  |
| LM vs. CT | scaf_00021:9892395 | 0.929 | 0.933 | 0.879 |  |  |  |  |  |
| LM vs. CT | scaf_00021:9892523 | 0.888 | 0.898 | 0.838 |  |  |  |  |  |
| LM vs. CT | scaf_00021:9912380 | 0.929 | 0.933 | 0.879 |  |  |  |  |  |
| LM vs. CT | scaf_00021:9914566 | 0.800 | 0.821 | 0.750 |  |  |  |  |  |
| LM vs. CT | scaf_00021:9914888 | 0.807 | 0.826 | 0.757 |  |  |  |  |  |
| LM vs. CT | scaf_00021:9915341 | 0.807 | 0.826 | 0.757 |  |  |  |  |  |
| LM vs. CT | scaf_00022:4738067 | 0.798 | 0.819 | 0.752 |  |  |  |  |  |
| LM vs. CT | scaf_00022:4981790 | 0.808 | 0.829 | 0.762 | scaf_00022:4978700-5003112 | Dnm1l |  | synonymous |  |
| LM vs. CT | scaf_00023:4444527 | 0.803 | 0.813 | 0.754 | scaf_00023:4444198-4461813 | mtg1 |  | synonymous | energy supply |
| LM vs. CT | scaf_00023:6715601 | 0.813 | 0.831 | 0.763 |  |  |  |  |  |
| LM vs. CT | scaf_00023:6715643 | 0.813 | 0.831 | 0.763 |  |  |  |  |  |
| LM vs. CT | scaf_00023:6715673 | 0.813 | 0.831 | 0.763 |  |  |  |  |  |
| LM vs. CT | scaf_00023:6715730 | 0.818 | 0.821 | 0.769 |  |  |  |  |  |
| LM vs. CT | scaf_00024:10489126 | 0.893 | 0.893 | 0.843 | scaf_00024:10488414-10527952 | C05D11.1 |  | synonymous |  |
| LM vs. CT | scaf_00024:4769603 | 0.926 | 0.931 | 0.876 |  |  |  |  |  |
| LM vs. CT | scaf_00024:4770918 | 0.850 | 0.860 | 0.800 |  |  |  |  |  |
| LM vs. CT | scaf_00024:4816369 | 0.812 | 0.824 | 0.762 |  |  |  |  |  |
| LM vs. CT | scaf_00024:4816434 | 0.812 | 0.824 | 0.762 |  |  |  |  |  |
| LM vs. CT | scaf_00024:4817274 | 0.812 | 0.824 | 0.762 |  |  |  |  |  |
| LM vs. CT | scaf_00024:4817491 | 0.812 | 0.824 | 0.762 |  |  |  |  |  |
| LM vs. CT | scaf_00024:4818195 | 0.812 | 0.824 | 0.762 | scaf_00024:4817578-4821969 |  | 3' UTR |  |  |
| LM vs. CT | scaf_00024:4818406 | 0.812 | 0.824 | 0.762 | scaf_00024:4817578-4821969 |  | 3' UTR |  |  |
| LM vs. CT | scaf_00024:4846085 | 0.888 | 0.895 | 0.838 |  |  |  |  |  |
| LM vs. CT | scaf_00024:5534963 | 0.776 | 0.786 | 0.726 |  |  |  |  |  |
| LM vs. CT | scaf_00026:12092245 | 0.820 | 0.833 | 0.775 | scaf_00026:12090500-12102803 | KLHL8 |  | synonymous |  |
| LM vs. CT | scaf_00026:12229062 | 0.844 | 0.857 | 0.798 |  |  |  |  |  |
| LM vs. CT | scaf_00026:7667539 | 0.786 | 0.786 | 0.740 |  |  |  |  |  |
| LM vs. CT | scaf_00026:7667675 | 0.783 | 0.786 | 0.737 |  |  |  |  |  |
| LM vs. CT | scaf_00026:7668941 | 0.786 | 0.786 | 0.740 |  |  |  |  |  |
| LM vs. CT | scaf_00026:8953868 | 0.850 | 0.864 | 0.805 |  |  |  |  |  |
| LM vs. CT | scaf_00026:9019235 | 0.844 | 0.857 | 0.798 | scaf_00026:9019145-9032627 | PGR | CDS | synonymous | reproduction |
| LM vs. CT | scaf_00028:11072595 | 0.786 | 0.800 | 0.736 |  |  |  |  |  |
| LM vs. CT | scaf_00028:11072801 | 0.821 | 0.821 | 0.772 |  |  |  |  |  |
| LM vs. CT | scaf_00028:11074944 | 0.783 | 0.786 | 0.733 |  |  |  |  |  |
| LM vs. CT | scaf_00028:11075258 | 0.783 | 0.786 | 0.733 |  |  |  |  |  |
| LM vs. CT | scaf_00028:11089606 | 0.801 | 0.792 | 0.751 | scaf_00028:11084083-11108337 | Dnajc14 | CDS | synonymous | cellular transporting |
| LM vs. CT | scaf_00028:4590459 | 0.790 | 0.800 | 0.740 |  |  |  |  |  |
| LM vs. CT | scaf_00028:4590477 | 0.883 | 0.890 | 0.834 |  |  |  |  |  |
| LM vs. CT | scaf_00028:8852545 | 0.820 | 0.833 | 0.771 | scaf_00028:8743806-8895023 | Ttn | CDS | synonymous | tissue development |
| LM vs. CT | scaf_00028:8863864 | 0.796 | 0.810 | 0.747 | scaf_00028:8743806-8895023 | Ttn | CDS | synonymous | tissue development |
| LM vs. CT | scaf_00028:9250660 | 0.808 | 0.829 | 0.758 | scaf_00028:9250060-9267279 | DNPEP |  | synonymous | protein synthesis, folding, metabolism |
| LM vs. CT | scaf_00029:11720184 | 0.926 | 0.931 | 0.875 | scaf_00029:11713274-11727240 | his-71 |  | synonymous | chromatin assembly and stabilization |
| LM vs. CT | scaf_00029:3961175 | 0.786 | 0.800 | 0.734 | scaf_00029:3960696-3966152 | mrpl33 |  | synonymous | energy supply |
| LM vs. CT | scaf_00029:3961176 | 0.786 | 0.800 | 0.734 | scaf_00029:3960696-3966152 | mrpl33 |  | synonymous | energy supply |
| LM vs. CT | scaf_00029:4631704 | 0.786 | 0.800 | 0.734 | scaf_00029:4631546-4669357 | CRIM1 |  | synonymous |  |
| LM vs. CT | scaf_00029:5542761 | 0.816 | 0.821 | 0.765 |  |  |  |  |  |
| LM vs. CT | scaf_00029:5544146 | 0.823 | 0.833 | 0.772 |  |  |  |  |  |
| LM vs. CT | scaf_00029:5544179 | 0.823 | 0.833 | 0.772 |  |  |  |  |  |
| LM vs. CT | scaf_00029:5546380 | 0.823 | 0.833 | 0.772 | scaf_00029:5544539-5556225 | ZNF106 | CDS | nonsynonymous | tissue development |
| LM vs. CT | scaf_00029:5557492 | 0.823 | 0.833 | 0.772 |  |  |  |  |  |
| LM vs. CT | scaf_00029:5557536 | 0.823 | 0.833 | 0.772 |  |  |  |  |  |
| LM vs. CT | scaf_00029:5575960 | 0.810 | 0.821 | 0.759 |  |  |  |  |  |
| LM vs. CT | scaf_00029:5575970 | 0.810 | 0.821 | 0.759 |  |  |  |  |  |
| LM vs. CT | scaf_00029:5575994 | 0.848 | 0.857 | 0.796 |  |  |  |  |  |
| LM vs. CT | scaf_00029:5637752 | 0.857 | 0.857 | 0.806 | scaf_00029:5623437-5639402 | Pak4 |  | synonymous | cellular signaling |
| LM vs. CT | scaf_00029:5640918 | 0.818 | 0.821 | 0.767 | scaf_00029:5637773-5649136 | PLCB1 |  | nonsynonymous | cellular signaling |
| LM vs. CT | scaf_00029:5643341 | 0.800 | 0.813 | 0.749 | scaf_00029:5637773-5649136 | PLCB1 | CDS | nonsynonymous | cellular signaling |
| LM vs. CT | scaf_00029:5644508 | 0.878 | 0.864 | 0.827 | scaf_00029:5637773-5649136 | PLCB1 | CDS | nonsynonymous | cellular signaling |
| LM vs. CT | scaf_00029:5654703 | 0.888 | 0.895 | 0.837 | scaf_00029:5649422-5671034 | PLCB1 |  | synonymous | cellular signaling |
| LM vs. CT | scaf_00029:5780414 | 0.884 | 0.895 | 0.833 |  |  |  |  |  |
| LM vs. CT | scaf_00029:5780460 | 0.799 | 0.818 | 0.748 |  |  |  |  |  |
| LM vs. CT | scaf_00029:5780484 | 0.799 | 0.818 | 0.748 |  |  |  |  |  |
| LM vs. CT | scaf_00029:5781897 | 0.799 | 0.818 | 0.748 |  |  |  |  |  |
| LM vs. CT | scaf_00029:5782216 | 0.841 | 0.856 | 0.790 |  |  |  |  |  |
| LM vs. CT | scaf_00029:5841986 | 0.821 | 0.821 | 0.770 | scaf_00029:5835983-5845333 | EHD3 | CDS | synonymous |  |
| LM vs. CT | scaf_00029:5847749 | 0.835 | 0.850 | 0.783 | scaf_00029:5846425-5853524 | Rdh14 |  | synonymous | enzyme |
| LM vs. CT | scaf_00029:5848080 | 0.920 | 0.917 | 0.868 | scaf_00029:5846425-5853524 | Rdh14 |  | synonymous | enzyme |
| LM vs. CT | scaf_00029:5850561 | 0.929 | 0.933 | 0.878 | scaf_00029:5846425-5853524 | Rdh14 |  | synonymous | enzyme |
| LM vs. CT | scaf_00029:5854754 | 0.929 | 0.933 | 0.878 |  |  |  |  |  |
| LM vs. CT | scaf_00030:2923790 | 0.858 | 0.867 | 0.787 |  |  |  |  |  |
| LM vs. CT | scaf_00030:8136841 | 0.786 | 0.786 | 0.715 |  |  |  |  |  |
| LM vs. CT | scaf_00031:10061798 | 0.818 | 0.821 | 0.737 |  |  |  |  |  |
| LM vs. CT | scaf_00031:10158924 | 0.821 | 0.821 | 0.740 | scaf_00031:10155477-10171948 | HMGCR | CDS | synonymous |  |
| LM vs. CT | scaf_00031:10265666 | 0.806 | 0.828 | 0.725 |  |  |  |  |  |
| LM vs. CT | scaf_00031:10265983 | 0.813 | 0.831 | 0.731 |  |  |  |  |  |
| LM vs. CT | scaf_00031:520366 | 0.789 | 0.800 | 0.707 | scaf_00031:513030-522853 | RAB11A | CDS | nonsynonymous | cellular transporting |
| LM vs. CT | scaf_00031:537257 | 0.789 | 0.800 | 0.707 | scaf_00031:529294-556939 | MARCH3 |  | synonymous | cellular transporting |
| LM vs. CT | scaf_00031:760466 | 0.789 | 0.800 | 0.707 |  |  |  |  |  |
| LM vs. CT | scaf_00031:7816195 | 0.790 | 0.800 | 0.709 | scaf_00031:7797987-7865580 | Sin3a | CDS | synonymous |  |
| LM vs. CT | scaf_00032:6209998 | 0.848 | 0.862 | 0.809 |  |  |  |  |  |
| LM vs. CT | scaf_00033:5344043 | 0.792 | 0.816 | 0.710 |  |  |  |  |  |
| LM vs. CT | scaf_00033:5349218 | 0.858 | 0.867 | 0.777 |  |  |  |  |  |
| LM vs. CT | scaf_00033:9845841 | 0.823 | 0.837 | 0.742 | scaf_00033:9835302-9853611 | Bbs2 |  | synonymous |  |
| LM vs. CT | scaf_00035:10067639 | 0.854 | 0.857 | 0.773 | scaf_00035:10063053-10070180 | Ola1 | CDS | synonymous |  |
| LM vs. CT | scaf_00035:1489720 | 0.790 | 0.808 | 0.708 | scaf_00035:1429259-1496489 | ACE |  | synonymous | osmoregulation? |
| LM vs. CT | scaf_00035:1489729 | 0.790 | 0.808 | 0.708 | scaf_00035:1429259-1496489 | ACE |  | synonymous | osmoregulation? |
| LM vs. CT | scaf_00035:1489753 | 0.790 | 0.808 | 0.708 | scaf_00035:1429259-1496489 | ACE |  | synonymous | osmoregulation? |
| LM vs. CT | scaf_00035:5489709 | 0.894 | 0.900 | 0.812 | scaf_00035:5489009-5495657 |  | 3' UTR |  |  |
| LM vs. CT | scaf_00035:5786214 | 0.844 | 0.833 | 0.763 |  |  |  |  |  |
| LM vs. CT | scaf_00035:6244160 | 0.888 | 0.898 | 0.807 |  |  |  |  |  |
| LM vs. CT | scaf_00035:6244551 | 0.888 | 0.895 | 0.806 |  |  |  |  |  |
| LM vs. CT | scaf_00035:8745998 | 0.791 | 0.773 | 0.709 | scaf_00035:8730458-8764910 | ttc25 |  | synonymous | cilia |
| LM vs. CT | scaf_00035:8758057 | 0.929 | 0.929 | 0.847 | scaf_00035:8730458-8764910 | ttc25 | CDS | synonymous | cilia |
| LM vs. CT | scaf_00036:8775008 | 0.835 | 0.846 | 0.794 | scaf_00036:8773964-8801165 | BAHD1 |  | synonymous | RNA synthesis |
| LM vs. CT | scaf_00036:9459237 | 0.783 | 0.786 | 0.741 |  |  |  |  |  |
| LM vs. CT | scaf_00037:292242 | 0.807 | 0.826 | 0.765 |  |  |  |  |  |
| LM vs. CT | scaf_00037:292543 | 0.807 | 0.826 | 0.765 | scaf_00037:292533-293835 |  | CDS |  |  |
| LM vs. CT | scaf_00037:293334 | 0.820 | 0.833 | 0.778 | scaf_00037:292533-293835 |  | intron |  |  |
| LM vs. CT | scaf_00037:298150 | 0.787 | 0.821 | 0.745 |  |  |  |  |  |
| LM vs. CT | scaf_00038:8288714 | 0.800 | 0.813 | 0.758 |  |  |  |  |  |
| LM vs. CT | scaf_00039:8149303 | 0.780 | 0.797 | 0.741 | scaf_00039:8136540-8152860 | PARK7 |  | synonymous | stress response |
| LM vs. CT | scaf_00039:8758170 | 0.799 | 0.818 | 0.760 | scaf_00039:8751351-8760008 | DTX3 | CDS | synonymous | cell cycle progression |
| LM vs. CT | scaf_00042:10080817 | 0.783 | 0.810 | 0.728 | scaf_00042:10078923-10119982 | ARHGEF3 |  | synonymous | tissue development |
| LM vs. CT | scaf_00042:10760972 | 0.776 | 0.786 | 0.721 | scaf_00042:10759987-10764842 |  | CDS |  |  |
| LM vs. CT | scaf_00042:10762636 | 0.813 | 0.831 | 0.758 | scaf_00042:10759987-10764842 |  | CDS |  |  |
| LM vs. CT | scaf_00042:5523277 | 0.807 | 0.826 | 0.752 |  |  |  |  |  |
| LM vs. CT | scaf_00042:6052814 | 0.821 | 0.821 | 0.767 |  |  |  |  |  |
| LM vs. CT | scaf_00042:6052858 | 0.783 | 0.786 | 0.728 |  |  |  |  |  |
| LM vs. CT | scaf_00043:4696539 | 0.964 | 0.967 | 0.913 | scaf_00043:4665840-4707262 | GHR |  | synonymous | growth |
| LM vs. CT | scaf_00043:4788709 | 0.888 | 0.895 | 0.837 | scaf_00043:4779991-4810658 | OXCT1 | CDS | nonsynonymous | food supply |
| LM vs. CT | scaf_00043:4791382 | 0.808 | 0.829 | 0.756 | scaf_00043:4779991-4810658 | OXCT1 | CDS | nonsynonymous | food supply |
| LM vs. CT | scaf_00043:4960528 | 0.846 | 0.867 | 0.794 |  |  |  |  |  |
| LM vs. CT | scaf_00043:4960741 | 0.794 | 0.800 | 0.743 |  |  |  |  |  |
| LM vs. CT | scaf_00043:4961421 | 0.855 | 0.867 | 0.804 |  |  |  |  |  |
| LM vs. CT | scaf_00043:4961855 | 0.855 | 0.867 | 0.804 |  |  |  |  |  |
| LM vs. CT | scaf_00043:9528384 | 0.816 | 0.821 | 0.765 |  |  |  |  |  |
| LM vs. CT | scaf_00044:1073684 | 0.807 | 0.826 | 0.761 | scaf_00044:1073613-1080615 | RBM22 |  | synonymous | mRNA splicing |
| LM vs. CT | scaf_00044:479149 | 0.807 | 0.826 | 0.761 |  |  |  |  |  |
| LM vs. CT | scaf_00044:499248 | 0.888 | 0.898 | 0.842 | scaf_00044:480242-523789 | MIPEP |  | synonymous |  |
| LM vs. CT | scaf_00044:741316 | 0.920 | 0.917 | 0.874 | scaf_00044:675262-743793 | Fam13c |  | synonymous | unknown |
| LM vs. CT | scaf_00044:741324 | 0.848 | 0.846 | 0.802 | scaf_00044:675262-743793 | Fam13c |  | synonymous | unknown |
| LM vs. CT | scaf_00044:741535 | 0.821 | 0.821 | 0.776 | scaf_00044:675262-743793 | Fam13c |  | synonymous | unknown |
| LM vs. CT | scaf_00044:742366 | 0.857 | 0.857 | 0.811 | scaf_00044:675262-743793 | Fam13c |  | synonymous | unknown |
| LM vs. CT | scaf_00044:742380 | 0.857 | 0.857 | 0.811 | scaf_00044:675262-743793 | Fam13c |  | synonymous | unknown |
| LM vs. CT | scaf_00044:8814071 | 0.858 | 0.867 | 0.813 | scaf_00044:8803426-8821768 | ANKHD1 | CDS | synonymous |  |
| LM vs. CT | scaf_00045:1219291 | 0.848 | 0.862 | 0.806 |  |  |  |  |  |
| LM vs. CT | scaf_00045:1219301 | 0.848 | 0.862 | 0.806 |  |  |  |  |  |
| LM vs. CT | scaf_00045:1219304 | 0.807 | 0.826 | 0.765 |  |  |  |  |  |
| LM vs. CT | scaf_00045:1219563 | 0.842 | 0.857 | 0.800 |  |  |  |  |  |
| LM vs. CT | scaf_00045:1219640 | 0.889 | 0.893 | 0.848 |  |  |  |  |  |
| LM vs. CT | scaf_00045:1220034 | 0.783 | 0.786 | 0.741 |  |  |  |  |  |
| LM vs. CT | scaf_00047:4778144 | 0.789 | 0.800 | 0.743 |  |  |  |  |  |
| LM vs. CT | scaf_00047:4788854 | 0.848 | 0.857 | 0.802 | scaf_00047:4780138-4817658 | YLPM1 |  | synonymous | protein synthesis, folding, metabolism |
| LM vs. CT | scaf_00047:4800690 | 0.857 | 0.857 | 0.811 | scaf_00047:4780138-4817658 | YLPM1 |  | synonymous | protein synthesis, folding, metabolism |
| LM vs. CT | scaf_00047:8765931 | 0.813 | 0.831 | 0.767 |  |  |  |  |  |
| LM vs. CT | scaf_00047:8838887 | 0.854 | 0.867 | 0.808 | scaf_00047:8827592-8867467 | Clasrp | CDS | synonymous | mRNA splicing |
| LM vs. CT | scaf_00047:8845325 | 0.806 | 0.828 | 0.760 | scaf_00047:8827592-8867467 | Clasrp |  | synonymous | mRNA splicing |
| LM vs. CT | scaf_00047:8845583 | 0.882 | 0.900 | 0.836 | scaf_00047:8827592-8867467 | Clasrp |  | synonymous | mRNA splicing |
| LM vs. CT | scaf_00048:4866377 | 0.888 | 0.898 | 0.807 | scaf_00048:4841930-4868942 | slc25a36a |  | synonymous | energy supply |
| LM vs. CT | scaf_00048:4866389 | 0.888 | 0.898 | 0.807 | scaf_00048:4841930-4868942 | slc25a36a |  | synonymous | energy supply |
| LM vs. CT | scaf_00050:1474944 | 0.879 | 0.875 | 0.808 | scaf_00050:1473492-1582900 | ascc3 |  | synonymous | DNA replication, repair, modification |
| LM vs. CT | scaf_00050:7362470 | 0.807 | 0.826 | 0.736 |  |  |  |  |  |
| LM vs. CT | scaf_00051:6282874 | 0.786 | 0.800 | 0.734 | scaf_00051:6280726-6288416 | SELT | CDS | synonymous |  |
| LM vs. CT | scaf_00051:6302100 | 0.804 | 0.826 | 0.753 | scaf_00051:6301806-6310380 | TSC22D1 |  | synonymous | tumor related |
| LM vs. CT | scaf_00051:6305691 | 0.874 | 0.887 | 0.823 | scaf_00051:6301806-6310380 | TSC22D1 |  | synonymous | tumor related |
| LM vs. CT | scaf_00051:6305694 | 0.828 | 0.845 | 0.777 | scaf_00051:6301806-6310380 | TSC22D1 |  | synonymous | tumor related |
| LM vs. CT | scaf_00051:6306573 | 0.792 | 0.816 | 0.740 | scaf_00051:6301806-6310380 | TSC22D1 |  | synonymous | tumor related |
| LM vs. CT | scaf_00051:6306574 | 0.792 | 0.816 | 0.740 | scaf_00051:6301806-6310380 | TSC22D1 |  | synonymous | tumor related |
| LM vs. CT | scaf_00051:6309462 | 0.882 | 0.900 | 0.831 | scaf_00051:6301806-6310380 | TSC22D1 |  | synonymous | tumor related |
| LM vs. CT | scaf_00051:7630284 | 0.855 | 0.867 | 0.804 |  |  |  |  |  |
| LM vs. CT | scaf_00051:7678466 | 0.779 | 0.800 | 0.728 | scaf_00051:7659893-7685971 | ESD |  | synonymous | formaldehyde detoxification |
| LM vs. CT | scaf_00051:7680755 | 0.786 | 0.800 | 0.734 | scaf_00051:7659893-7685971 | ESD | CDS | synonymous | formaldehyde detoxification |
| LM vs. CT | scaf_00051:8143373 | 0.920 | 0.925 | 0.869 |  |  |  |  |  |
| LM vs. CT | scaf_00051:8144245 | 0.923 | 0.928 | 0.872 |  |  |  |  |  |
| LM vs. CT | scaf_00051:8145139 | 0.964 | 0.964 | 0.913 |  |  |  |  |  |
| LM vs. CT | scaf_00051:8145146 | 0.964 | 0.964 | 0.913 |  |  |  |  |  |
| LM vs. CT | scaf_00051:8145291 | 0.963 | 0.964 | 0.912 |  |  |  |  |  |
| LM vs. CT | scaf_00051:8145906 | 0.963 | 0.962 | 0.912 |  |  |  |  |  |
| LM vs. CT | scaf_00051:8148075 | 0.926 | 0.931 | 0.875 |  |  |  |  |  |
| LM vs. CT | scaf_00051:8148293 | 0.926 | 0.931 | 0.875 |  |  |  |  |  |
| LM vs. CT | scaf_00051:8148816 | 0.926 | 0.931 | 0.875 |  |  |  |  |  |
| LM vs. CT | scaf_00051:8148955 | 0.926 | 0.931 | 0.875 |  |  |  |  |  |
| LM vs. CT | scaf_00051:8150875 | 0.926 | 0.931 | 0.875 |  |  |  |  |  |
| LM vs. CT | scaf_00051:997258 | 0.848 | 0.857 | 0.796 |  |  |  |  |  |
| LM vs. CT | scaf_00051:997322 | 0.808 | 0.808 | 0.757 |  |  |  |  |  |
| LM vs. CT | scaf_00051:997475 | 0.961 | 0.962 | 0.910 |  |  |  |  |  |
| LM vs. CT | scaf_00051:997492 | 0.831 | 0.846 | 0.780 |  |  |  |  |  |
| LM vs. CT | scaf_00052:4089608 | 0.808 | 0.829 | 0.762 | scaf_00052:4088811-4102136 | Arrdc3 |  | synonymous | cellular signaling |
| LM vs. CT | scaf_00053:5440510 | 0.854 | 0.857 | 0.819 |  |  |  |  |  |
| LM vs. CT | scaf_00053:5542943 | 0.786 | 0.786 | 0.751 | scaf_00053:5527252-5547522 | MRC1 |  | synonymous | stress response |
| LM vs. CT | scaf_00053:5542987 | 0.786 | 0.786 | 0.751 | scaf_00053:5527252-5547522 | MRC1 |  | synonymous | stress response |
| LM vs. CT | scaf_00053:5542992 | 0.786 | 0.786 | 0.751 | scaf_00053:5527252-5547522 | MRC1 |  | synonymous | stress response |
| LM vs. CT | scaf_00053:5543036 | 0.786 | 0.786 | 0.751 | scaf_00053:5527252-5547522 | MRC1 |  | synonymous | stress response |
| LM vs. CT | scaf_00054:796563 | 0.962 | 0.964 | 0.912 | scaf_00054:784018-804398 | ZZEF1 |  | synonymous |  |
| LM vs. CT | scaf_00054:796586 | 0.961 | 0.958 | 0.912 | scaf_00054:784018-804398 | ZZEF1 |  | synonymous |  |
| LM vs. CT | scaf_00057:1264430 | 0.776 | 0.786 | 0.730 | scaf_00057:1255791-1266415 | PRELP |  | synonymous | connective tissue extracellular matrix |
| LM vs. CT | scaf_00057:1264467 | 0.783 | 0.786 | 0.737 | scaf_00057:1255791-1266415 | PRELP |  | synonymous | connective tissue extracellular matrix |
| LM vs. CT | scaf_00057:4696799 | 0.779 | 0.810 | 0.733 |  |  |  |  |  |
| LM vs. CT | scaf_00057:785693 | 0.850 | 0.864 | 0.805 |  |  |  |  |  |
| LM vs. CT | scaf_00059:5536730 | 0.782 | 0.809 | 0.744 |  |  |  |  |  |
| LM vs. CT | scaf_00059:5537889 | 0.808 | 0.829 | 0.769 |  |  |  |  |  |
| LM vs. CT | scaf_00060:2851210 | 0.874 | 0.864 | 0.828 |  |  |  |  |  |
| LM vs. CT | scaf_00060:2851229 | 0.833 | 0.818 | 0.787 |  |  |  |  |  |
| LM vs. CT | scaf_00060:2932816 | 0.888 | 0.895 | 0.842 | scaf_00060:2925220-2939403 | Mrpl9 |  | synonymous |  |
| LM vs. CT | scaf_00060:3154079 | 0.835 | 0.850 | 0.789 | scaf_00060:3144506-3161861 | Snrpa1 |  | synonymous | mRNA splicing |
| LM vs. CT | scaf_00060:3154348 | 0.835 | 0.852 | 0.789 | scaf_00060:3144506-3161861 | Snrpa1 |  | synonymous | mRNA splicing |
| LM vs. CT | scaf_00060:3994934 | 0.828 | 0.845 | 0.782 | scaf_00060:3986850-3997974 | Dcaf12 | CDS | synonymous |  |
| LM vs. CT | scaf_00061:1593548 | 0.798 | 0.808 | 0.748 |  |  |  |  |  |
| LM vs. CT | scaf_00061:1747239 | 0.787 | 0.798 | 0.737 |  |  |  |  |  |
| LM vs. CT | scaf_00061:3084080 | 0.850 | 0.860 | 0.800 |  |  |  |  |  |
| LM vs. CT | scaf_00062:58311 | 0.813 | 0.831 | 0.775 |  |  |  |  |  |
| LM vs. CT | scaf_00063:1163967 | 0.779 | 0.800 | 0.733 | scaf_00063:1154375-1165246 | fs(1)h |  | synonymous | unknown |
| LM vs. CT | scaf_00063:1164019 | 0.840 | 0.867 | 0.794 | scaf_00063:1154375-1165246 | fs(1)h |  | synonymous | unknown |
| LM vs. CT | scaf_00063:3492283 | 0.793 | 0.810 | 0.747 |  |  |  |  |  |
| LM vs. CT | scaf_00063:3493128 | 0.841 | 0.856 | 0.796 |  |  |  |  |  |
| LM vs. CT | scaf_00063:3493206 | 0.835 | 0.850 | 0.789 |  |  |  |  |  |
| LM vs. CT | scaf_00063:3493919 | 0.828 | 0.845 | 0.782 |  |  |  |  |  |
| LM vs. CT | scaf_00063:3493931 | 0.828 | 0.845 | 0.782 |  |  |  |  |  |
| LM vs. CT | scaf_00063:3494271 | 0.885 | 0.893 | 0.840 |  |  |  |  |  |
| LM vs. CT | scaf_00063:3494519 | 0.834 | 0.849 | 0.788 |  |  |  |  |  |
| LM vs. CT | scaf_00063:3494707 | 0.843 | 0.851 | 0.798 |  |  |  |  |  |
| LM vs. CT | scaf_00063:3494913 | 0.839 | 0.858 | 0.793 |  |  |  |  |  |
| LM vs. CT | scaf_00063:3495169 | 0.894 | 0.900 | 0.848 |  |  |  |  |  |
| LM vs. CT | scaf_00063:3497440 | 0.814 | 0.808 | 0.768 |  |  |  |  |  |
| LM vs. CT | scaf_00063:4184871 | 0.799 | 0.818 | 0.753 |  |  |  |  |  |
| LM vs. CT | scaf_00063:4184973 | 0.807 | 0.826 | 0.761 |  |  |  |  |  |
| LM vs. CT | scaf_00063:4185023 | 0.799 | 0.818 | 0.753 |  |  |  |  |  |
| LM vs. CT | scaf_00063:4185044 | 0.790 | 0.808 | 0.744 |  |  |  |  |  |
| LM vs. CT | scaf_00063:4185315 | 0.807 | 0.826 | 0.761 |  |  |  |  |  |
| LM vs. CT | scaf_00063:4185383 | 0.807 | 0.826 | 0.761 |  |  |  |  |  |
| LM vs. CT | scaf_00063:4185736 | 0.807 | 0.826 | 0.761 |  |  |  |  |  |
| LM vs. CT | scaf_00063:4185857 | 0.799 | 0.818 | 0.753 |  |  |  |  |  |
| LM vs. CT | scaf_00063:4185860 | 0.799 | 0.818 | 0.753 |  |  |  |  |  |
| LM vs. CT | scaf_00063:4185861 | 0.799 | 0.818 | 0.753 |  |  |  |  |  |
| LM vs. CT | scaf_00063:4185933 | 0.790 | 0.808 | 0.744 |  |  |  |  |  |
| LM vs. CT | scaf_00063:4186027 | 0.799 | 0.818 | 0.753 |  |  |  |  |  |
| LM vs. CT | scaf_00063:4186162 | 0.807 | 0.826 | 0.761 |  |  |  |  |  |
| LM vs. CT | scaf_00063:4186335 | 0.807 | 0.826 | 0.761 |  |  |  |  |  |
| LM vs. CT | scaf_00063:4186544 | 0.807 | 0.826 | 0.761 |  |  |  |  |  |
| LM vs. CT | scaf_00063:4186603 | 0.807 | 0.826 | 0.761 |  |  |  |  |  |
| LM vs. CT | scaf_00064:3596763 | 0.819 | 0.837 | 0.773 |  |  |  |  |  |
| LM vs. CT | scaf_00073:3002248 | 0.850 | 0.860 | 0.768 | scaf_00073:2999204-3043545 | eral1 |  | synonymous | energy supply |
| LM vs. CT | scaf_00073:3015021 | 0.846 | 0.846 | 0.765 | scaf_00073:2999204-3043545 | eral1 |  | synonymous | energy supply |
| LM vs. CT | scaf_00073:3015204 | 0.844 | 0.833 | 0.763 | scaf_00073:2999204-3043545 | eral1 |  | synonymous | energy supply |
| LM vs. CT | scaf_00074:650929 | 0.850 | 0.860 | 0.768 |  |  |  |  |  |
| LM vs. CT | scaf_00076:1051663 | 0.848 | 0.862 | 0.766 |  |  |  |  |  |
| LM vs. CT | scaf_00076:1051709 | 0.848 | 0.862 | 0.766 |  |  |  |  |  |
| LM vs. CT | scaf_00076:1051824 | 0.848 | 0.862 | 0.766 |  |  |  |  |  |
| LM vs. CT | scaf_00076:1052025 | 0.812 | 0.824 | 0.730 |  |  |  |  |  |
| LM vs. CT | scaf_00076:413835 | 0.848 | 0.862 | 0.766 | scaf_00076:407411-418727 | SRRM2 |  | synonymous | mRNA splicing |
| LM vs. CT | scaf_00076:462534 | 0.879 | 0.887 | 0.797 | scaf_00076:457647-473742 | Ints10 |  | synonymous |  |
| LM vs. CT | scaf_00076:515600 | 0.843 | 0.857 | 0.761 | scaf_00076:503319-517572 | MTHFSD | CDS | synonymous |  |
| LM vs. CT | scaf_00078:552273 | 0.848 | 0.862 | 0.798 |  |  |  |  |  |
| LM vs. CT | scaf_00078:552354 | 0.848 | 0.862 | 0.798 |  |  |  |  |  |
| LM vs. CT | scaf_00078:552367 | 0.848 | 0.862 | 0.798 |  |  |  |  |  |
| LM vs. CT | scaf_00078:552369 | 0.848 | 0.862 | 0.798 |  |  |  |  |  |
| LM vs. CT | scaf_00078:552390 | 0.848 | 0.862 | 0.798 |  |  |  |  |  |
| LM vs. CT | scaf_00078:552588 | 0.804 | 0.829 | 0.755 |  |  |  |  |  |
| LM vs. CT | scaf_00079:1462105 | 0.786 | 0.786 | 0.704 | scaf_00079:1435929-1462624 | GRIP2 |  | synonymous | cellular signaling |
| LM vs. CT | scaf_00084:58740 | 0.893 | 0.893 | 0.838 |  |  |  |  |  |
| LM vs. CT | scaf_00097:379268 | 0.828 | 0.830 | 0.773 | scaf_00097:316687-411305 | PGM3 |  | synonymous |  |
| LM vs. CT | scaf_00099:567893 | 0.808 | 0.829 | 0.726 |  |  |  |  |  |
| LM vs. CT | scaf_00108:314463 | 0.874 | 0.888 | 0.793 | scaf_00108:312297-314477 | Pkd2 |  | synonymous |  |
| LM vs. CT | scaf_00108:314608 | 0.838 | 0.854 | 0.757 |  |  |  |  |  |
| LM vs. CT | scaf_00108:314625 | 0.838 | 0.854 | 0.757 |  |  |  |  |  |
| LM vs. CT | scaf_00108:317145 | 0.800 | 0.823 | 0.718 |  |  |  |  |  |
| LM vs. CT | scaf_00108:657768 | 0.929 | 0.933 | 0.847 | scaf_00108:655578-669507 | CTSL | CDS | nonsynonymous | protein synthesis, folding, metabolism |
| LM vs. CT | scaf_00109:487084 | 0.835 | 0.850 | 0.785 | scaf_00109:470513-488917 | IDI1 |  | synonymous |  |
| LM vs. CT | scaf_00110:101666 | 0.798 | 0.819 | 0.798 |  |  |  |  |  |
| LM vs. CT | scaf_00111:670894 | 0.793 | 0.821 | 0.736 |  |  |  |  |  |
| LM vs. CT | scaf_00112:73465 | 0.874 | 0.885 | 0.793 | scaf_00112:72162-74747 | Cdkn1a |  | synonymous |  |
| LM vs. CT | scaf_00120:449716 | 0.929 | 0.929 | 0.869 |  |  |  |  |  |
| LM vs. CT | scaf_00120:450824 | 0.960 | 0.955 | 0.900 |  |  |  |  |  |
| LM vs. CT | scaf_00122:486351 | 0.850 | 0.860 | 0.800 | scaf_00122:483944-494313 | Deptor | CDS | synonymous |  |
| LM vs. CT | scaf_00122:494097 | 0.807 | 0.826 | 0.757 | scaf_00122:483944-494313 | Deptor |  | synonymous |  |
| LM vs. CT | scaf_00122:495737 | 0.848 | 0.862 | 0.798 |  |  |  |  |  |
| LM vs. CT | scaf_00122:509034 | 0.779 | 0.786 | 0.730 | scaf_00122:506440-532793 | Ept1 |  | synonymous |  |
| LM vs. CT | scaf_00125:636711 | 0.841 | 0.846 | 0.784 |  |  |  |  |  |
| LM vs. CT | scaf_00127:6835 | 0.783 | 0.786 | 0.737 |  |  |  |  |  |
| LM vs. CT | scaf_00136:300853 | 0.858 | 0.867 |  | scaf_00136:298999-334004 | CD109 | CDS | synonymous | cellular signaling |
| LM vs. CT | scaf_00136:365269 | 0.964 | 0.964 |  |  |  |  |  |  |
| LM vs. CT | scaf_00136:454319 | 0.780 | 0.786 |  | scaf_00136:449651-463700 | PRPF6 |  | synonymous |  |
| LM vs. CT | scaf_00136:486412 | 0.893 | 0.893 |  |  |  |  |  |  |
| LM vs. CT | scaf_00147:138965 | 0.782 | 0.800 | 0.701 | scaf_00147:127611-152976 | Itsn1 |  | synonymous | cellular transporting |
| LM vs. CT | scaf_00147:217247 | 0.881 | 0.885 | 0.800 | scaf_00147:206323-218247 | slc16a10 | CDS | synonymous |  |
| LM vs. CT | scaf_00158:263573 | 0.858 | 0.867 | 0.777 |  |  |  |  |  |
| LM vs. CT | scaf_00179:271177 | 0.780 | 0.797 | 0.729 |  |  |  |  |  |
| LM vs. CT | scaf_00184:27047 | 0.786 | 0.786 |  | scaf_00184:12469-62551 | CHD4 |  | synonymous |  |
| LM vs. CT | scaf_00256:34936 | 0.888 | 0.895 | 0.806 |  |  |  |  |  |
| LM vs. CT | scaf_00256:35271 | 0.926 | 0.933 | 0.845 |  |  |  |  |  |
| LM vs. CT | scaf_00257:32209 | 0.779 | 0.785 | 0.779 | scaf_00257:11244-33430 |  | intron |  |  |
| LM vs. CT | scaf_00257:32447 | 1.000 | 1.000 | 1.000 | scaf_00257:11244-33430 |  | intron |  |  |
| LM vs. CT | scaf_00522:14907 | 0.821 | 0.821 |  | scaf_00522:3001-14930 | LOXL3 |  | synonymous |  |
| LM vs. CT | scaf_00633:28004 | 0.844 | 0.833 |  | scaf_00633:27938-30336 | PCDHGC3 | CDS | nonsynonymous | cellular component assembly |
| LM vs. CT | scaf_01104:8004 | 0.799 | 0.818 |  | scaf_01104:6791-11897 | BDH1 | CDS | synonymous |  |
| LM vs. CT | scaf_01338:18308 | 0.804 | 0.829 |  |  |  |  |  |  |
| LM vs. CT | scaf_01631:23557 | 0.873 | 0.876 |  |  |  |  |  |  |
| LM vs. CT | scaf_01973:57 | 0.813 | 0.831 |  |  |  |  |  |  |
| LM vs. CT | scaf_03269:2492 | 0.786 | 0.786 | 0.760 |  |  |  |  |  |
| LM vs. CT | scaf_03807:6929 | 0.792 | 0.816 |  |  |  |  |  |  |
| LM vs. CT | scaf_05144:2960 | 0.789 | 0.800 |  |  |  |  |  |  |
| LM vs. CT | scaf_05762:2886 | 0.964 | 0.967 |  |  |  |  |  |  |
| LM vs. CT | scaf_06110:4189 | 0.800 | 0.813 | 0.800 |  |  |  |  |  |
| LM vs. CT | scaf_06591:6484 | 0.808 | 0.829 |  |  |  |  |  |  |
| LM vs. CT | scaf_06870:4561 | 0.813 | 0.821 |  |  |  |  |  |  |
| LC vs. CT | scaf_00001:23443287 | 0.960 | 0.958 | 0.923 | scaf_00001:23426749-23446970 | WIPF2 |  | synonymous |  |
| LC vs. CT | scaf_00001:23453508 | 0.956 | 0.955 | 0.919 |  |  |  |  |  |
| LC vs. CT | scaf_00001:25658247 | 0.956 | 0.955 | 0.919 |  |  |  |  |  |
| LC vs. CT | scaf_00001:25741282 | 0.960 | 0.958 | 0.923 |  |  |  |  |  |
| LC vs. CT | scaf_00001:25746179 | 0.960 | 0.958 | 0.923 |  |  |  |  |  |
| LC vs. CT | scaf_00001:26238542 | 0.957 | 0.958 | 0.919 |  |  |  |  |  |
| LC vs. CT | scaf_00002:22554464 | 0.960 | 0.964 | 0.921 |  |  |  |  |  |
| LC vs. CT | scaf_00002:22554654 | 0.960 | 0.964 | 0.921 |  |  |  |  |  |
| LC vs. CT | scaf_00002:22636985 | 0.960 | 0.958 | 0.921 |  |  |  |  |  |
| LC vs. CT | scaf_00002:22637983 | 0.960 | 0.958 | 0.921 |  |  |  |  |  |
| LC vs. CT | scaf_00002:24949897 | 1.000 | 1.000 | 0.961 |  |  |  |  |  |
| LC vs. CT | scaf_00003:11948741 | 0.960 | 0.964 | 0.923 |  |  |  |  |  |
| LC vs. CT | scaf_00003:11960734 | 1.000 | 1.000 | 0.963 | scaf_00003:11955837-11969202 | pomc |  | synonymous | preprotein |
| LC vs. CT | scaf_00003:15127212 | 0.958 | 0.958 | 0.921 | scaf_00003:15108590-15127767 | KDR |  | synonymous |  |
| LC vs. CT | scaf_00003:15127860 | 0.960 | 0.958 | 0.923 |  |  |  |  |  |
| LC vs. CT | scaf_00003:15608899 | 1.000 | 1.000 | 0.963 | scaf_00003:15605441-15615652 | nit2b | CDS | synonymous |  |
| LC vs. CT | scaf_00004:16299574 | 0.960 | 0.964 | 0.928 | scaf_00004:16298997-16305565 | krt13 |  | synonymous |  |
| LC vs. CT | scaf_00004:20307429 | 1.000 | 1.000 | 0.968 |  |  |  |  |  |
| LC vs. CT | scaf_00005:17461742 | 0.958 | 0.955 | 0.926 |  |  |  |  |  |
| LC vs. CT | scaf_00006:5212951 | 0.959 | 0.964 | 0.898 | scaf_00006:5183553-5213087 | OCLN |  | synonymous |  |
| LC vs. CT | scaf_00007:15849814 | 1.000 | 1.000 | 0.963 | scaf_00007:15840843-15859409 | Tctn2 |  | synonymous | cellular signaling |
| LC vs. CT | scaf_00007:15853914 | 0.960 | 0.964 | 0.923 | scaf_00007:15840843-15859409 | Tctn2 |  | synonymous | cellular signaling |
| LC vs. CT | scaf_00007:15903648 | 0.960 | 0.964 | 0.923 | scaf_00007:15903405-15919758 |  | 3' UTR |  |  |
| LC vs. CT | scaf_00007:16498424 | 0.960 | 0.964 | 0.923 | scaf_00007:16454955-16500014 | Cltc |  | synonymous | cellular transporting |
| LC vs. CT | scaf_00008:316170 | 0.960 | 0.964 | 0.928 |  |  |  |  |  |
| LC vs. CT | scaf_00008:316234 | 0.960 | 0.964 | 0.928 |  |  |  |  |  |
| LC vs. CT | scaf_00009:1096276 | 0.960 | 0.958 | 0.923 | scaf_00009:1060270-1096956 | WDR81 |  | synonymous | cellular transporting |
| LC vs. CT | scaf_00009:1155389 | 0.956 | 0.955 | 0.919 | scaf_00009:1140574-1155770 | NPRL2 |  | synonymous |  |
| LC vs. CT | scaf_00009:1284408 | 0.960 | 0.964 | 0.923 | scaf_00009:1222605-1303270 | Pdgfra |  | synonymous | tissue development |
| LC vs. CT | scaf_00009:7851759 | 0.960 | 0.958 | 0.923 | scaf_00009:7846190-7862752 | ARHGAP35 |  | synonymous |  |
| LC vs. CT | scaf_00010:4480649 | 0.957 | 0.964 | 0.926 |  |  |  |  |  |
| LC vs. CT | scaf_00010:4645716 | 1.000 | 1.000 | 0.969 |  |  |  |  |  |
| LC vs. CT | scaf_00010:4651137 | 1.000 | 1.000 | 0.969 | scaf_00010:4645722-4677525 | POT1 |  | synonymous | chromatin assembly and stabilization |
| LC vs. CT | scaf_00010:8992010 | 0.954 | 0.950 | 0.924 | scaf_00010:8989313-9010960 | Asb14 | CDS | synonymous |  |
| LC vs. CT | scaf_00011:119323 | 0.960 | 0.964 | 0.928 |  |  |  |  |  |
| LC vs. CT | scaf_00012:12062868 | 0.960 | 0.964 | 0.928 |  |  |  |  |  |
| LC vs. CT | scaf_00013:468270 | 0.960 | 0.958 | 0.929 | scaf_00013:467757-474897 | OAZ | CDS | synonymous |  |
| LC vs. CT | scaf_00014:2673166 | 0.960 | 0.964 | 0.923 |  |  |  |  |  |
| LC vs. CT | scaf_00014:2673175 | 0.960 | 0.964 | 0.923 |  |  |  |  |  |
| LC vs. CT | scaf_00014:2673177 | 0.960 | 0.964 | 0.923 |  |  |  |  |  |
| LC vs. CT | scaf_00014:2673314 | 1.000 | 1.000 | 0.963 |  |  |  |  |  |
| LC vs. CT | scaf_00014:2673318 | 0.960 | 0.964 | 0.923 |  |  |  |  |  |
| LC vs. CT | scaf_00014:2673319 | 0.960 | 0.964 | 0.923 |  |  |  |  |  |
| LC vs. CT | scaf_00014:2673330 | 0.957 | 0.962 | 0.919 |  |  |  |  |  |
| LC vs. CT | scaf_00014:2673357 | 0.959 | 0.964 | 0.921 |  |  |  |  |  |
| LC vs. CT | scaf_00014:2673358 | 0.959 | 0.964 | 0.921 |  |  |  |  |  |
| LC vs. CT | scaf_00014:2673380 | 0.959 | 0.964 | 0.921 |  |  |  |  |  |
| LC vs. CT | scaf_00014:2737498 | 0.960 | 0.964 | 0.923 |  |  |  |  |  |
| LC vs. CT | scaf_00014:2738067 | 0.960 | 0.964 | 0.923 |  |  |  |  |  |
| LC vs. CT | scaf_00014:2738148 | 0.960 | 0.964 | 0.923 |  |  |  |  |  |
| LC vs. CT | scaf_00014:2738183 | 0.960 | 0.964 | 0.923 |  |  |  |  |  |
| LC vs. CT | scaf_00014:2738185 | 0.960 | 0.964 | 0.923 |  |  |  |  |  |
| LC vs. CT | scaf_00014:2738570 | 0.960 | 0.964 | 0.923 |  |  |  |  |  |
| LC vs. CT | scaf_00014:2738761 | 0.960 | 0.964 | 0.923 |  |  |  |  |  |
| LC vs. CT | scaf_00014:2738788 | 0.960 | 0.964 | 0.923 |  |  |  |  |  |
| LC vs. CT | scaf_00014:2738996 | 0.960 | 0.964 | 0.923 |  |  |  |  |  |
| LC vs. CT | scaf_00014:2739098 | 0.960 | 0.964 | 0.923 |  |  |  |  |  |
| LC vs. CT | scaf_00014:2740820 | 0.960 | 0.964 | 0.923 | scaf_00014:2740231-2763574 | dhdh |  | nonsynonymous | enzyme |
| LC vs. CT | scaf_00014:2740939 | 0.960 | 0.964 | 0.923 | scaf_00014:2740231-2763574 | dhdh | CDS | nonsynonymous | enzyme |
| LC vs. CT | scaf_00014:2741182 | 0.958 | 0.962 | 0.921 | scaf_00014:2740231-2763574 | dhdh |  | nonsynonymous | enzyme |
| LC vs. CT | scaf_00015:276980 | 0.958 | 0.958 | 0.926 |  |  |  |  |  |
| LC vs. CT | scaf_00015:3031149 | 0.960 | 0.964 | 0.928 | scaf_00015:3030628-3032312 |  | intron |  |  |
| LC vs. CT | scaf_00016:6041903 | 1.000 | 1.000 | 0.968 | scaf_00016:6041862-6050713 | Rala | CDS | nonsynonymous | cellular signaling |
| LC vs. CT | scaf_00017:616506 | 1.000 | 1.000 | 0.971 | scaf_00017:603400-628436 | RIOK3 |  | synonymous | cellular component assembly |
| LC vs. CT | scaf_00017:658978 | 1.000 | 1.000 | 0.971 | scaf_00017:651637-669253 | SRSF6 |  | synonymous | mRNA splicing |
| LC vs. CT | scaf_00019:3935062 | 1.000 | 1.000 | 0.963 |  |  |  |  |  |
| LC vs. CT | scaf_00019:3983067 | 0.958 | 0.962 | 0.921 | scaf_00019:3957040-3986041 |  | CDS |  |  |
| LC vs. CT | scaf_00020:13525735 | 0.960 | 0.958 | 0.927 |  |  |  |  |  |
| LC vs. CT | scaf_00020:5467002 | 0.960 | 0.958 | 0.927 |  |  |  |  |  |
| LC vs. CT | scaf_00021:10875290 | 0.960 | 0.964 | 0.923 | scaf_00021:10820076-10878117 | SHROOM2 |  | synonymous | retinal pigmentation |
| LC vs. CT | scaf_00021:10877632 | 0.960 | 0.964 | 0.923 | scaf_00021:10820076-10878117 | SHROOM2 |  | synonymous | retinal pigmentation |
| LC vs. CT | scaf_00022:807960 | 0.960 | 0.958 | 0.923 | scaf_00022:778434-810753 | Gnai1 |  | synonymous | cellular signaling |
| LC vs. CT | scaf_00022:807982 | 0.960 | 0.958 | 0.923 | scaf_00022:778434-810753 | Gnai1 |  | synonymous | cellular signaling |
| LC vs. CT | scaf_00025:12126967 | 0.960 | 0.964 | 0.928 |  |  |  |  |  |
| LC vs. CT | scaf_00025:3250296 | 0.960 | 0.958 | 0.927 |  |  |  |  |  |
| LC vs. CT | scaf_00027:5707010 | 0.958 | 0.958 | 0.921 | scaf_00027:5706616-5737644 | Eogt |  | synonymous | cellular signaling |
| LC vs. CT | scaf_00029:12368830 | 0.958 | 0.962 | 0.919 |  |  |  |  |  |
| LC vs. CT | scaf_00029:12483263 | 0.954 | 0.950 | 0.915 |  |  |  |  |  |
| LC vs. CT | scaf_00029:12863305 | 0.960 | 0.958 | 0.921 |  |  |  |  |  |
| LC vs. CT | scaf_00031:6072568 | 0.957 | 0.958 | 0.889 |  |  |  |  |  |
| LC vs. CT | scaf_00031:6075658 | 1.000 | 1.000 | 0.933 |  |  |  |  |  |
| LC vs. CT | scaf_00031:8583148 | 0.960 | 0.958 | 0.893 |  |  |  |  |  |
| LC vs. CT | scaf_00033:10612740 | 0.960 | 0.964 | 0.893 | scaf_00033:10607183-10617895 | DNAJA1 | CDS | nonsynonymous | stress response |
| LC vs. CT | scaf_00035:2279965 | 0.960 | 0.958 | 0.893 | scaf_00035:2264046-2298216 | DHX8 |  | synonymous | RNA synthesis |
| LC vs. CT | scaf_00035:2282037 | 0.960 | 0.958 | 0.893 | scaf_00035:2264046-2298216 | DHX8 | CDS | synonymous | RNA synthesis |
| LC vs. CT | scaf_00035:2297623 | 0.960 | 0.958 | 0.893 | scaf_00035:2264046-2298216 | DHX8 |  | synonymous | RNA synthesis |
| LC vs. CT | scaf_00035:2297664 | 0.960 | 0.958 | 0.893 | scaf_00035:2264046-2298216 | DHX8 |  | synonymous | RNA synthesis |
| LC vs. CT | scaf_00035:2306642 | 0.958 | 0.958 | 0.891 | scaf_00035:2306478-2337834 | Hat1 |  | synonymous | chromatin assembly and stabilization |
| LC vs. CT | scaf_00035:2319705 | 0.960 | 0.958 | 0.893 | scaf_00035:2306478-2337834 | Hat1 |  | synonymous | chromatin assembly and stabilization |
| LC vs. CT | scaf_00035:2320059 | 0.958 | 0.955 | 0.891 | scaf_00035:2306478-2337834 | Hat1 |  | synonymous | chromatin assembly and stabilization |
| LC vs. CT | scaf_00035:2320292 | 0.958 | 0.958 | 0.891 | scaf_00035:2306478-2337834 | Hat1 |  | synonymous | chromatin assembly and stabilization |
| LC vs. CT | scaf_00035:2320691 | 0.956 | 0.955 | 0.889 | scaf_00035:2306478-2337834 | Hat1 |  | synonymous | chromatin assembly and stabilization |
| LC vs. CT | scaf_00035:2320898 | 0.956 | 0.950 | 0.889 | scaf_00035:2306478-2337834 | Hat1 |  | synonymous | chromatin assembly and stabilization |
| LC vs. CT | scaf_00035:2320900 | 0.956 | 0.950 | 0.889 | scaf_00035:2306478-2337834 | Hat1 |  | synonymous | chromatin assembly and stabilization |
| LC vs. CT | scaf_00035:2320928 | 0.956 | 0.950 | 0.889 | scaf_00035:2306478-2337834 | Hat1 |  | synonymous | chromatin assembly and stabilization |
| LC vs. CT | scaf_00035:2320978 | 0.958 | 0.955 | 0.891 | scaf_00035:2306478-2337834 | Hat1 |  | synonymous | chromatin assembly and stabilization |
| LC vs. CT | scaf_00035:2321378 | 0.958 | 0.955 | 0.891 | scaf_00035:2306478-2337834 | Hat1 |  | synonymous | chromatin assembly and stabilization |
| LC vs. CT | scaf_00035:2322076 | 0.960 | 0.958 | 0.893 | scaf_00035:2306478-2337834 | Hat1 |  | synonymous | chromatin assembly and stabilization |
| LC vs. CT | scaf_00035:2334343 | 1.000 | 1.000 | 0.933 | scaf_00035:2306478-2337834 | Hat1 | CDS | synonymous | chromatin assembly and stabilization |
| LC vs. CT | scaf_00035:2336677 | 0.960 | 0.964 | 0.893 | scaf_00035:2306478-2337834 | Hat1 |  | synonymous | chromatin assembly and stabilization |
| LC vs. CT | scaf_00036:3063891 | 0.960 | 0.964 | 0.928 | scaf_00036:3061820-3066170 | SPRTN | CDS | nonsynonymous | DNA replication, repair, modification |
| LC vs. CT | scaf_00036:3112859 | 0.960 | 0.964 | 0.928 | scaf_00036:3089224-3115939 | PYGL | CDS | nonsynonymous | food supply |
| LC vs. CT | scaf_00036:3118431 | 0.960 | 0.964 | 0.928 |  |  |  |  |  |
| LC vs. CT | scaf_00036:3121169 | 0.960 | 0.964 | 0.928 | scaf_00036:3120012-3127801 | Capns1 |  | synonymous | cellular signaling |
| LC vs. CT | scaf_00036:3172071 | 0.960 | 0.958 | 0.927 | scaf_00036:3170097-3175900 | CAPN3 | CDS | synonymous |  |
| LC vs. CT | scaf_00036:3221629 | 0.960 | 0.964 | 0.928 | scaf_00036:3211078-3222867 | TMEM87A |  | synonymous | cellular transporting |
| LC vs. CT | scaf_00036:3221641 | 0.960 | 0.964 | 0.928 | scaf_00036:3211078-3222867 | TMEM87A |  | synonymous | cellular transporting |
| LC vs. CT | scaf_00040:5372559 | 0.958 | 0.962 | 0.941 |  |  |  |  |  |
| LC vs. CT | scaf_00040:5372620 | 0.958 | 0.962 | 0.941 |  |  |  |  |  |
| LC vs. CT | scaf_00041:2870572 | 0.960 | 0.964 | 0.928 | scaf_00041:2863263-2871085 |  | CDS |  |  |
| LC vs. CT | scaf_00042:5443182 | 0.960 | 0.958 | 0.920 | scaf_00042:5430872-5443225 | Rbm17 |  | synonymous |  |
| LC vs. CT | scaf_00044:8553473 | 0.960 | 0.964 | 0.928 |  |  |  |  |  |
| LC vs. CT | scaf_00045:5000399 | 0.957 | 0.958 | 0.924 |  |  |  |  |  |
| LC vs. CT | scaf_00045:5000400 | 0.957 | 0.958 | 0.924 |  |  |  |  |  |
| LC vs. CT | scaf_00049:4607385 | 1.000 | 1.000 | 0.963 |  |  |  |  |  |
| LC vs. CT | scaf_00049:4608104 | 1.000 | 1.000 | 0.963 |  |  |  |  |  |
| LC vs. CT | scaf_00049:4608498 | 0.960 | 0.964 | 0.923 |  |  |  |  |  |
| LC vs. CT | scaf_00049:4608500 | 0.960 | 0.964 | 0.923 |  |  |  |  |  |
| LC vs. CT | scaf_00049:4619908 | 1.000 | 1.000 | 0.963 | scaf_00049:4609394-4620022 |  | 5' UTR |  |  |
| LC vs. CT | scaf_00049:4619924 | 1.000 | 1.000 | 0.963 | scaf_00049:4609394-4620022 |  | 5' UTR |  |  |
| LC vs. CT | scaf_00049:7212369 | 1.000 | 1.000 | 0.963 | scaf_00049:7211839-7214827 | LAGE3 |  | nonsynonymous |  |
| LC vs. CT | scaf_00049:7212516 | 1.000 | 1.000 | 0.963 | scaf_00049:7211839-7214827 | LAGE3 |  | nonsynonymous |  |
| LC vs. CT | scaf_00049:7212609 | 1.000 | 1.000 | 0.963 | scaf_00049:7211839-7214827 | LAGE3 |  | nonsynonymous |  |
| LC vs. CT | scaf_00049:7212998 | 0.955 | 0.958 | 0.917 | scaf_00049:7211839-7214827 | LAGE3 | CDS | nonsynonymous |  |
| LC vs. CT | scaf_00049:7597313 | 0.959 | 0.964 | 0.921 | scaf_00049:7591730-7601413 | Isy1 | CDS | synonymous |  |
| LC vs. CT | scaf_00049:7632507 | 1.000 | 1.000 | 0.963 | scaf_00049:7624332-7637090 |  | CDS |  |  |
| LC vs. CT | scaf_00049:7641689 | 1.000 | 1.000 | 0.963 | scaf_00049:7638659-7654927 | Cog5 | CDS | synonymous |  |
| LC vs. CT | scaf_00049:7647882 | 1.000 | 1.000 | 0.963 | scaf_00049:7638659-7654927 | Cog5 |  | synonymous |  |
| LC vs. CT | scaf_00050:5846654 | 1.000 | 1.000 | 0.944 |  |  |  |  |  |
| LC vs. CT | scaf_00050:5847175 | 0.960 | 0.964 | 0.904 |  |  |  |  |  |
| LC vs. CT | scaf_00050:5850704 | 0.960 | 0.964 | 0.904 |  |  |  |  |  |
| LC vs. CT | scaf_00052:7892070 | 0.957 | 0.962 | 0.924 | scaf_00052:7887020-7893056 |  | intron |  |  |
| LC vs. CT | scaf_00052:7933096 | 0.960 | 0.964 | 0.928 |  |  |  |  |  |
| LC vs. CT | scaf_00052:7936982 | 0.958 | 0.962 | 0.926 |  |  |  |  |  |
| LC vs. CT | scaf_00053:3237661 | 0.955 | 0.958 | 0.928 |  |  |  |  |  |
| LC vs. CT | scaf_00053:3237740 | 0.957 | 0.958 | 0.930 |  |  |  |  |  |
| LC vs. CT | scaf_00053:3237781 | 0.958 | 0.958 | 0.932 |  |  |  |  |  |
| LC vs. CT | scaf_00059:5641033 | 1.000 | 1.000 | 0.969 |  |  |  |  |  |
| LC vs. CT | scaf_00063:2208588 | 0.952 | 0.950 | 0.920 |  |  |  |  |  |
| LC vs. CT | scaf_00069:2438112 | 1.000 | 1.000 | 0.968 | scaf_00069:2437817-2445211 | Dok1 |  | synonymous | cellular signaling |
| LC vs. CT | scaf_00071:1761963 | 0.958 | 0.958 | 0.926 | scaf_00071:1747403-1783310 | POLR2A |  | synonymous | RNA synthesis |
| LC vs. CT | scaf_00071:1762817 | 0.960 | 0.958 | 0.927 | scaf_00071:1747403-1783310 | POLR2A |  | synonymous | RNA synthesis |
| LC vs. CT | scaf_00071:1785788 | 0.960 | 0.958 | 0.927 |  |  |  |  |  |
| LC vs. CT | scaf_00071:21576 | 0.960 | 0.964 | 0.928 |  |  |  |  |  |
| LC vs. CT | scaf_00081:1248476 | 0.960 | 0.964 | 0.960 |  |  |  |  |  |
| LC vs. CT | scaf_00081:1248791 | 0.960 | 0.964 | 0.960 |  |  |  |  |  |
| LC vs. CT | scaf_00081:1249139 | 0.960 | 0.964 | 0.960 |  |  |  |  |  |
| LC vs. CT | scaf_00081:1249257 | 0.958 | 0.962 | 0.958 |  |  |  |  |  |
| LC vs. CT | scaf_00081:1249340 | 0.958 | 0.962 | 0.958 |  |  |  |  |  |
| LC vs. CT | scaf_00081:1249537 | 0.958 | 0.962 | 0.958 |  |  |  |  |  |
| LC vs. CT | scaf_00081:1249686 | 0.960 | 0.964 | 0.960 |  |  |  |  |  |
| LC vs. CT | scaf_00081:1249698 | 0.958 | 0.962 | 0.958 |  |  |  |  |  |
| LC vs. CT | scaf_00081:1250194 | 0.955 | 0.962 | 0.955 |  |  |  |  |  |
| LC vs. CT | scaf_00089:710413 | 0.960 | 0.958 | 0.929 |  |  |  |  |  |
| LC vs. CT | scaf_00089:711633 | 0.958 | 0.958 | 0.928 |  |  |  |  |  |
| LC vs. CT | scaf_00099:297650 | 0.960 | 0.958 | 0.893 |  |  |  |  |  |
| LC vs. CT | scaf_00099:326665 | 0.960 | 0.958 | 0.893 | scaf_00099:304609-328067 | IGF2BP2 |  | synonymous |  |
| LC vs. CT | scaf_00122:539016 | 0.960 | 0.958 | 0.927 | scaf_00122:535289-541147 | Mapre3 |  | synonymous | tumor related |
| LC vs. CT | scaf_00143:216200 | 1.000 | 1.000 | 0.933 | scaf_00143:212465-216502 | NUDT3 |  | synonymous |  |
| LC vs. CT | scaf_00143:328072 | 0.960 | 0.958 | 0.893 | scaf_00143:308233-330850 | GNS |  | synonymous | enzyme |
| LC vs. CT | scaf_00169:241990 | 0.959 | 0.964 | 0.959 |  |  |  |  |  |
| LC vs. CT | scaf_00194:167128 | 0.960 | 0.964 | 0.893 |  |  |  |  |  |
| LC vs. CT | scaf_00194:167175 | 0.960 | 0.964 | 0.893 |  |  |  |  |  |
| LC vs. CT | scaf_00202:22476 | 1.000 | 1.000 |  |  |  |  |  |  |
| LC vs. CT | scaf_00202:40252 | 0.957 | 0.958 |  |  |  |  |  |  |
| LC vs. CT | scaf_00202:40253 | 0.957 | 0.958 |  |  |  |  |  |  |
| LC vs. CT | scaf_00202:40693 | 0.958 | 0.958 |  |  |  |  |  |  |
| LC vs. CT | scaf_00206:190317 | 0.960 | 0.958 | 0.923 |  |  |  |  |  |
| LC vs. CT | scaf_00208:165005 | 0.958 | 0.962 | 0.910 |  |  |  |  |  |
| LC vs. CT | scaf_00208:165795 | 0.959 | 0.964 | 0.910 |  |  |  |  |  |
| LC vs. CT | scaf_00208:95961 | 0.960 | 0.958 | 0.911 | scaf_00208:63575-96216 | ikbkap |  | synonymous | cellular signaling |
| LC vs. CT | scaf_00260:112139 | 1.000 | 1.000 | 0.933 |  |  |  |  |  |
| LC vs. CT | scaf_00260:112142 | 1.000 | 1.000 | 0.933 |  |  |  |  |  |
| LC vs. CT | scaf_00260:44886 | 0.957 | 0.962 | 0.890 |  |  |  |  |  |
| LC vs. CT | scaf_00260:45236 | 0.958 | 0.962 | 0.891 |  |  |  |  |  |
| LC vs. CT | scaf_00260:45244 | 0.958 | 0.962 | 0.891 |  |  |  |  |  |
| LC vs. CT | scaf_00260:45712 | 0.954 | 0.955 | 0.887 |  |  |  |  |  |
| LC vs. CT | scaf_00260:45922 | 0.958 | 0.962 | 0.891 |  |  |  |  |  |
| LC vs. CT | scaf_00260:45948 | 0.960 | 0.964 | 0.893 |  |  |  |  |  |
| LC vs. CT | scaf_00260:45988 | 0.960 | 0.964 | 0.893 |  |  |  |  |  |
| LC vs. CT | scaf_00260:45996 | 0.960 | 0.964 | 0.893 |  |  |  |  |  |
| LC vs. CT | scaf_00260:46044 | 0.960 | 0.964 | 0.893 |  |  |  |  |  |
| LC vs. CT | scaf_00260:46074 | 0.958 | 0.962 | 0.891 |  |  |  |  |  |
| LC vs. CT | scaf_00260:46134 | 0.958 | 0.962 | 0.891 |  |  |  |  |  |
| LC vs. CT | scaf_00260:46271 | 0.960 | 0.964 | 0.893 |  |  |  |  |  |
| LC vs. CT | scaf_00260:46335 | 1.000 | 1.000 | 0.933 |  |  |  |  |  |
| LC vs. CT | scaf_00260:46373 | 0.960 | 0.964 | 0.893 |  |  |  |  |  |
| LC vs. CT | scaf_00260:46374 | 0.960 | 0.964 | 0.893 |  |  |  |  |  |
| LC vs. CT | scaf_00260:46482 | 1.000 | 1.000 | 0.933 |  |  |  |  |  |
| LC vs. CT | scaf_00260:46512 | 0.958 | 0.962 | 0.891 |  |  |  |  |  |
| LC vs. CT | scaf_00260:46600 | 0.958 | 0.962 | 0.891 |  |  |  |  |  |
| LC vs. CT | scaf_00260:46636 | 0.958 | 0.962 | 0.891 |  |  |  |  |  |
| LC vs. CT | scaf_00260:46888 | 0.960 | 0.964 | 0.893 |  |  |  |  |  |
| LC vs. CT | scaf_00260:47004 | 1.000 | 1.000 | 0.933 |  |  |  |  |  |
| LC vs. CT | scaf_00260:47023 | 0.957 | 0.964 | 0.890 |  |  |  |  |  |
| LC vs. CT | scaf_00260:54291 | 1.000 | 1.000 | 0.933 |  |  |  |  |  |
| LC vs. CT | scaf_00260:54863 | 1.000 | 1.000 | 0.933 |  |  |  |  |  |
| LC vs. CT | scaf_00260:55079 | 1.000 | 1.000 | 0.933 |  |  |  |  |  |
| LC vs. CT | scaf_00260:55260 | 1.000 | 1.000 | 0.933 |  |  |  |  |  |
| LC vs. CT | scaf_00260:55345 | 1.000 | 1.000 | 0.933 |  |  |  |  |  |
| LC vs. CT | scaf_00260:56372 | 1.000 | 1.000 | 0.933 |  |  |  |  |  |
| LC vs. CT | scaf_00260:57077 | 1.000 | 1.000 | 0.933 |  |  |  |  |  |
| LC vs. CT | scaf_00260:6431 | 0.960 | 0.964 | 0.893 |  |  |  |  |  |
| LC vs. CT | scaf_00260:6799 | 1.000 | 1.000 | 0.933 |  |  |  |  |  |
| LC vs. CT | scaf_00398:22926 | 0.960 | 0.964 | 0.893 | scaf_00398:6479-23058 | DIN4 | 3' UTR |  | food supply |
| LC vs. CT | scaf_00467:39596 | 1.000 | 1.000 | 0.938 |  |  |  |  |  |
| LC vs. CT | scaf_00513:45653 | 1.000 | 1.000 | 0.896 |  |  |  |  |  |
| LC vs. CT | scaf_02432:8497 | 0.959 | 0.964 |  |  |  |  |  |  |
| LC vs. CT | scaf_04805:7157 | 1.000 | 1.000 |  | scaf_04805:5015-7799 | Ttll12 |  | synonymous | cell cycle progression |

**Table S3.** Full gene names of outlier genes retrieved from <https://david.ncifcrf.gov/list.jsp>.

| **Outlier gene** | **Full gene name** |
| --- | --- |
| ACE | angiotensin I converting enzyme(ACE) |
| ACRC | acidic repeat containing(ACRC) |
| ACVR1 | activin A receptor type 1(ACVR1) |
| Aff3 | AF4/FMR2 family member 3(AFF3) |
| Alg6 | ALG6, alpha-1,3-glucosyltransferase(ALG6) |
| ANKHD1 | ankyrin repeat and KH domain containing 1(ANKHD1) |
| AP1M1 | adaptor related protein complex 1 mu 1 subunit(AP1M1) |
| ARAP2 | ArfGAP with RhoGAP domain, ankyrin repeat and PH domain 2(ARAP2) |
| ARHGAP35 | Rho GTPase activating protein 35(ARHGAP35) |
| ARHGEF3 | Rho guanine nucleotide exchange factor 3(ARHGEF3) |
| Arrdc3 | arrestin domain containing 3(arrdc3) |
| Asb14 | ankyrin repeat and SOCS box containing 14(ASB14) |
| ascc3 | activating signal cointegrator 1 complex subunit 3(ASCC3) |
| BAHD1 | bromo adjacent homology domain containing 1(bahd1) |
| Bbs2 | Bardet-Biedl syndrome 2(BBS2) |
| BDH1 | 3-hydroxybutyrate dehydrogenase, type 1(BDH1) |
| C05D11.1 | hypothetical protein(C05D11.1) |
| CAPN3 | calpain 3(CAPN3) |
| Capns1 | calpain small subunit 1(CAPNS1) |
| CD109 | CD109 molecule(Cd109) |
| Cdh23 | cadherin related 23(CDH23) |
| Cdkl5 | cyclin dependent kinase like 5(CDKL5) |
| Cdkn1a | cyclin dependent kinase inhibitor 1A(CDKN1A) |
| CHD4 | chromodomain helicase DNA binding protein 4(CHD4) |
| Clasrp | CLK4 associating serine/arginine rich protein(CLASRP) |
| Cltc | clathrin heavy chain(CLTC) |
| Cog5 | component of oligomeric golgi complex 5(COG5) |
| coq6 | coenzyme Q6, monooxygenase(COQ6) |
| CRIM1 | cysteine rich transmembrane BMP regulator 1(CRIM1) |
| crtc3 | CREB regulated transcription coactivator 3(CRTC3) |
| CTSL | cathepsin L(ctsl) |
| Dcaf12 | DDB1 and CUL4 associated factor 12(DCAF12) |
| DDB_G0271982 | hypothetical protein(DDB_G0271982) |
| Deptor | DEP domain containing MTOR-interacting protein(DEPTOR) |
| dhdh | dihydrodiol dehydrogenase(DHDH) |
| DHX8 | DEAH-box helicase 8(DHX8) |
| DIN4 | Transketolase family protein(DIN4) |
| DNAJA1 | DnaJ heat shock protein family (Hsp40) member A1(DNAJA1) |
| Dnajc14 | DnaJ heat shock protein family (Hsp40) member C14(DNAJC14) |
| Dnm1l | dynamin 1 like(DNM1L) |
| DNPEP | aspartyl aminopeptidase(DNPEP) |
| Dok1 | docking protein 1(DOK1) |
| DTX3 | deltex E3 ubiquitin ligase 3(DTX3) |
| EHD3 | EH domain containing 3(EHD3) |
| Eogt | EGF domain specific O-linked N-acetylglucosamine transferase(EOGT) |
| Ept1 | ethanolaminephosphotransferase 1(Ept1) |
| eral1 | Era like 12S mitochondrial rRNA chaperone 1(ERAL1) |
| ESD | esterase D(ESD) |
| EXT2 | exostosin glycosyltransferase 2(ext2) |
| Fam13c | family with sequence similarity 13 member C(Fam13c) |
| fs(1)h | female sterile (1) homeotic(fs(1)h) |
| GHR | growth hormone receptor(GHR) |
| Gnai1 | G protein subunit alpha i1(GNAI1) |
| GNS | glucosamine (N-acetyl)-6-sulfatase(GNS) |
| GRIP2 | glutamate receptor interacting protein 2(GRIP2) |
| Hat1 | histone acetyltransferase 1(HAT1) |
| HEXB | hexosaminidase subunit beta(HEXB) |
| his-71 | Histone H3.3 type 1(his-71) |
| HMGCR | 3-hydroxy-3-methylglutaryl-CoA reductase(hmgcr) |
| hspa14 | heat shock protein family A (Hsp70) member 14(HSPA14) |
| IDI1 | isopentenyl-diphosphate delta isomerase 1(idi1) |
| IGF2BP2 | insulin like growth factor 2 mRNA binding protein 2(IGF2BP2) |
| ikbkap | inhibitor of kappa light polypeptide gene enhancer in B-cells, kinase complex-associated protein(IKBKAP) |
| Ints10 | integrator complex subunit 10(INTS10) |
| Isy1 | ISY1 splicing factor homolog(ISY1) |
| Itsn1 | intersectin 1(ITSN1) |
| KCNK1 | potassium two pore domain channel subfamily K member 1(KCNK1) |
| KDR | kinase insert domain receptor(KDR) |
| KLHL8 | kelch like family member 8(klhl8) |
| krt13 | keratin 13(KRT13) |
| LAGE3 | L antigen family member 3(LAGE3) |
| LOXL3 | lysyl oxidase like 3(LOXL3) |
| Lztr1 | leucine zipper like transcription regulator 1(LZTR1) |
| MAP2K3 | mitogen-activated protein kinase kinase 3(MAP2K3) |
| Mapre3 | microtubule associated protein RP/EB family member 3(MAPRE3) |
| MARCH3 | membrane associated ring-CH-type finger 3(MARCH3) |
| MIPEP | mitochondrial intermediate peptidase(MIPEP) |
| MOB2 | MOB kinase activator 2(MOB2) |
| Morn4 | MORN repeat containing 4(MORN4) |
| MRC1 | mannose receptor, C type 1(MRC1) |
| mrpl33 | ribosomal protein L33(mrpl33) |
| Mrpl9 | mitochondrial ribosomal protein L9(MRPL9) |
| mtg1 | mitochondrial ribosome associated GTPase 1(Mtg1) |
| MTHFSD | methenyltetrahydrofolate synthetase domain containing(mthfsd) |
| mybB | myb domain-containing protein(mybB) |
| NDUFA9 | NADH:ubiquinone oxidoreductase subunit A9(NDUFA9) |
| NMRK2 | nicotinamide riboside kinase 2(NMRK2) |
| NPRL2 | NPR2-like, GATOR1 complex subunit(NPRL2) |
| NUDT3 | nudix hydrolase 3(NUDT3) |
| OAZ | zinc finger protein 423 homolog(Oaz) |
| OCLN | occludin(OCLN) |
| Ola1 | Obg like ATPase 1(OLA1) |
| oplah | 5-oxoprolinase (ATP-hydrolysing)(OPLAH) |
| OSBPL5 | oxysterol binding protein like 5(OSBPL5) |
| Ostf1 | osteoclast stimulating factor 1(OSTF1) |
| OXCT1 | 3-oxoacid CoA-transferase 1(OXCT1) |
| Pak4 | p21 (RAC1) activated kinase 4(Pak4) |
| PARK7 | Parkinsonism associated deglycase(PARK7) |
| PCDHGC3 | protocadherin gamma subfamily C, 3(PCDHGC3) |
| Pdgfra | platelet derived growth factor receptor alpha(PDGFRA) |
| PGM3 | phosphoglucomutase 3(pgm3) |
| PGR | progesterone receptor(PGR) |
| Phactr1 | phosphatase and actin regulator 1(PHACTR1) |
| Phb | prohibitin(Phb) |
| Pkd2 | polycystin 2, transient receptor potential cation channel(PKD2) |
| PLCB1 | phospholipase C beta 1(Plcb1) |
| Pofut2 | protein O-fucosyltransferase 2(pofut2) |
| POLR2A | RNA polymerase II subunit A(polr2a) |
| pomc | proopiomelanocortin(POMC) |
| POT1 | protection of telomeres 1(Pot1) |
| PRELP | proline and arginine rich end leucine rich repeat protein(Prelp) |
| PRPF6 | pre-mRNA processing factor 6(prpf6) |
| PYGL | phosphorylase, glycogen, liver(PYGL) |
| RAB11A | RAB11A, member RAS oncogene family(RAB11A) |
| Rala | RAS like proto-oncogene A(Rala) |
| Rbm17 | RNA binding motif protein 17(RBM17) |
| RBM22 | RNA binding motif protein 22(RBM22) |
| Rdh14 | retinol dehydrogenase 14 (all-trans/9-cis/11-cis)(RDH14) |
| RELL1 | RELT like 1(RELL1) |
| RIOK3 | RIO kinase 3(RIOK3) |
| RNASEH2A | ribonuclease H2 subunit A(RNASEH2A) |
| SELT | selenoprotein T(selt) |
| Sf3b3 | splicing factor 3b subunit 3(SF3B3) |
| SHROOM2 | shroom family member 2(SHROOM2) |
| Sin3a | SIN3 transcription regulator family member A(SIN3A) |
| slc16a10 | solute carrier family 16 member 10(SLC16A10) |
| SLC25A15 | solute carrier family 25 member 15(Slc25a15) |
| slc25a36a | solute carrier family 25 (pyrimidine nucleotide carrier ), member 36a(slc25a36a) |
| SLC4A10 | solute carrier family 4 member 10(slc4a10) |
| Snrpa1 | small nuclear ribonucleoprotein polypeptide A'(snrpa1) |
| SPRTN | SprT-like N-terminal domain(SPRTN) |
| SRPR | signal recognition particle receptor ('docking protein')(Srpr) |
| SRRM2 | serine/arginine repetitive matrix 2(SRRM2) |
| SRSF6 | serine and arginine rich splicing factor 6(Srsf6) |
| St5 | suppression of tumorigenicity 5(st5) |
| Stard10 | StAR related lipid transfer domain containing 10(STARD10) |
| SZT2 | seizure threshold 2 homolog (mouse)(SZT2) |
| TBC1D23 | TBC1 domain family member 23(TBC1D23) |
| Tctn2 | tectonic family member 2(TCTN2) |
| TJP2 | tight junction protein 2(TJP2) |
| Tmc7 | transmembrane channel like 7(TMC7) |
| TMEM87A | transmembrane protein 87A(TMEM87A) |
| TSC22D1 | TSC22 domain family member 1(TSC22D1) |
| ttc25 | tetratricopeptide repeat domain 25(TTC25) |
| Ttll12 | tubulin tyrosine ligase-like family, member 12(ttll12) |
| Ttn | titin(TTN) |
| ucp2 | uncoupling protein 2(UCP2) |
| USP28 | ubiquitin specific peptidase 28(USP28) |
| USP47 | ubiquitin specific peptidase 47(USP47) |
| VSNL1 | visinin like 1(VSNL1) |
| WDR81 | WD repeat domain 81(Wdr81) |
| WIPF2 | WAS/WASL interacting protein family member 2(WIPF2) |
| YLPM1 | YLP motif containing 1(YLPM1) |
| ZNF106 | zinc finger protein 106(Znf106) |
| ZZEF1 | zinc finger ZZ-type and EF-hand domain containing 1(ZZEF1) |

**Table S4.** Gene ontology (GO) terms of biological process associated with outlier genes retrieved from <https://www.ebi.ac.uk/QuickGO/>.

| **Population** | **GO ID** | **GO term** | **No. outlier genes involved** |
| --- | --- | --- | --- |
| Lake Michigan vs. Connecticut River | GO:0006820 | anion transport | 2 |
| Lake Michigan vs. Connecticut River | GO:0019722 | calcium-mediated signaling | 3 |
| Lake Michigan vs. Connecticut River | GO:0005975 | carbohydrate metabolic process | 3 |
| Lake Michigan vs. Connecticut River | GO:0007155 | cell adhesion | 2 |
| Lake Michigan vs. Connecticut River | GO:0007050 | cell cycle arrest | 15 |
| Lake Michigan vs. Connecticut River | GO:0045454 | cell redox homeostasis | 1 |
| Lake Michigan vs. Connecticut River | GO:0007268 | chemical synaptic transmission | 4 |
| Lake Michigan vs. Connecticut River | GO:0006325 | chromatin organization | 1 |
| Lake Michigan vs. Connecticut River | GO:0015936 | coenzyme A metabolic process | 1 |
| Lake Michigan vs. Connecticut River | GO:0032456 | endocytic recycling | 1 |
| Lake Michigan vs. Connecticut River | GO:0046294 | formaldehyde catabolic process | 2 |
| Lake Michigan vs. Connecticut River | GO:0006024 | glycosaminoglycan biosynthetic process | 1 |
| Lake Michigan vs. Connecticut River | GO:0040007 | growth | 1 |
| Lake Michigan vs. Connecticut River | GO:0015012 | heparan sulfate proteoglycan biosynthetic process | 1 |
| Lake Michigan vs. Connecticut River | GO:0007156 | homophilic cell adhesion via plasma membrane adhesion molecules | 2 |
| Lake Michigan vs. Connecticut River | GO:0006886 | intracellular protein transport | 4 |
| Lake Michigan vs. Connecticut River | GO:0035556 | intracellular signal transduction | 7 |
| Lake Michigan vs. Connecticut River | GO:0008299 | isoprenoid biosynthetic process | 2 |
| Lake Michigan vs. Connecticut River | GO:0016042 | lipid catabolic process | 3 |
| Lake Michigan vs. Connecticut River | GO:0006629 | lipid metabolic process | 4 |
| Lake Michigan vs. Connecticut River | GO:0008152 | metabolic process | 6 |
| Lake Michigan vs. Connecticut River | GO:0000266 | mitochondrial fission | 1 |
| Lake Michigan vs. Connecticut River | GO:0006839 | mitochondrial transport | 5 |
| Lake Michigan vs. Connecticut River | GO:0000398 | mRNA splicing, via spliceosome | 1 |
| Lake Michigan vs. Connecticut River | GO:1905515 | non-motile cilium assembly | 1 |
| Lake Michigan vs. Connecticut River | GO:0006913 | nucleocytoplasmic transport | 1 |
| Lake Michigan vs. Connecticut River | GO:0071704 | organic substance metabolic process | 1 |
| Lake Michigan vs. Connecticut River | GO:0055114 | oxidation-reduction process | 5 |
| Lake Michigan vs. Connecticut River | GO:0016559 | peroxisome fission | 1 |
| Lake Michigan vs. Connecticut River | GO:0008654 | phospholipid biosynthetic process | 1 |
| Lake Michigan vs. Connecticut River | GO:0032793 | positive regulation of CREB transcription factor activity | 1 |
| Lake Michigan vs. Connecticut River | GO:0071805 | potassium ion transmembrane transport | 1 |
| Lake Michigan vs. Connecticut River | GO:0006457 | protein folding | 1 |
| Lake Michigan vs. Connecticut River | GO:0051289 | protein homotetramerization | 1 |
| Lake Michigan vs. Connecticut River | GO:0016560 | protein import into peroxisome matrix, docking | 3 |
| Lake Michigan vs. Connecticut River | GO:0006468 | protein phosphorylation | 10 |
| Lake Michigan vs. Connecticut River | GO:0015031 | protein transport | 2 |
| Lake Michigan vs. Connecticut River | GO:0016567 | protein ubiquitination | 1 |
| Lake Michigan vs. Connecticut River | GO:0006508 | proteolysis | 7 |
| Lake Michigan vs. Connecticut River | GO:0007265 | Ras protein signal transduction | 1 |
| Lake Michigan vs. Connecticut River | GO:0001558 | regulation of cell growth | 1 |
| Lake Michigan vs. Connecticut River | GO:0007346 | regulation of mitotic cell cycle | 1 |
| Lake Michigan vs. Connecticut River | GO:0035023 | regulation of Rho protein signal transduction | 2 |
| Lake Michigan vs. Connecticut River | GO:0006355 | regulation of transcription, DNA-templated | 9 |
| Lake Michigan vs. Connecticut River | GO:0007165 | signal transduction | 11 |
| Lake Michigan vs. Connecticut River | GO:0072331 | signal transduction by p53 class mediator | 1 |
| Lake Michigan vs. Connecticut River | GO:0007264 | small GTPase mediated signal transduction | 2 |
| Lake Michigan vs. Connecticut River | GO:0016180 | snRNA processing | 1 |
| Lake Michigan vs. Connecticut River | GO:0051225 | spindle assembly | 1 |
| Lake Michigan vs. Connecticut River | GO:0006614 | SRP-dependent cotranslational protein targeting to membrane | 1 |
| Lake Michigan vs. Connecticut River | GO:0006412 | translation | 3 |
| Lake Michigan vs. Connecticut River | GO:0007178 | transmembrane receptor protein serine/threonine kinase signaling pathway | 1 |
| Lake Michigan vs. Connecticut River | GO:0055085 | transmembrane transport | 1 |
| Lake Michigan vs. Connecticut River | GO:0006810 | transport | 6 |
| Lake Michigan vs. Connecticut River | GO:0006744 | ubiquinone biosynthetic process | 1 |
| Lake Michigan vs. Connecticut River | GO:0006511 | ubiquitin-dependent protein catabolic process | 2 |
| Lake Michigan vs. Connecticut River | GO:0016192 | vesicle-mediated transport | 2 |
| Lake Champlain vs. Connecticut River | GO:0007188 | adenylate cyclase-modulating G protein-coupled receptor signaling pathway | 2 |
| Lake Champlain vs. Connecticut River | GO:0070830 | bicellular tight junction assembly | 1 |
| Lake Champlain vs. Connecticut River | GO:0005975 | carbohydrate metabolic process | 1 |
| Lake Champlain vs. Connecticut River | GO:0006464 | cellular protein modification process | 1 |
| Lake Champlain vs. Connecticut River | GO:0006325 | chromatin organization | 13 |
| Lake Champlain vs. Connecticut River | GO:0006348 | chromatin silencing at telomere | 13 |
| Lake Champlain vs. Connecticut River | GO:0007186 | G protein-coupled receptor signaling pathway | 2 |
| Lake Champlain vs. Connecticut River | GO:0030203 | glycosaminoglycan metabolic process | 1 |
| Lake Champlain vs. Connecticut River | GO:0016573 | histone acetylation | 13 |
| Lake Champlain vs. Connecticut River | GO:0006886 | intracellular protein transport | 1 |
| Lake Champlain vs. Connecticut River | GO:0035556 | intracellular signal transduction | 1 |
| Lake Champlain vs. Connecticut River | GO:0006891 | intra-Golgi vesicle-mediated transport | 2 |
| Lake Champlain vs. Connecticut River | GO:0008152 | metabolic process | 2 |
| Lake Champlain vs. Connecticut River | GO:0006807 | nitrogen compound metabolic process | 1 |
| Lake Champlain vs. Connecticut River | GO:0048008 | platelet-derived growth factor receptor signaling pathway | 1 |
| Lake Champlain vs. Connecticut River | GO:0006457 | protein folding | 1 |
| Lake Champlain vs. Connecticut River | GO:0006468 | protein phosphorylation | 3 |
| Lake Champlain vs. Connecticut River | GO:0015031 | protein transport | 1 |
| Lake Champlain vs. Connecticut River | GO:0007265 | Ras protein signal transduction | 1 |
| Lake Champlain vs. Connecticut River | GO:0006355 | regulation of transcription, DNA-templated | 13 |
| Lake Champlain vs. Connecticut River | GO:0007165 | signal transduction | 4 |
| Lake Champlain vs. Connecticut River | GO:0007264 | small GTPase mediated signal transduction | 1 |
| Lake Champlain vs. Connecticut River | GO:0000723 | telomere maintenance | 1 |
| Lake Champlain vs. Connecticut River | GO:0007169 | transmembrane receptor protein tyrosine kinase signaling pathway | 1 |
| Lake Champlain vs. Connecticut River | GO:0048010 | vascular endothelial growth factor receptor signaling pathway | 1 |
| Lake Champlain vs. Connecticut River | GO:0016192 | vesicle-mediated transport | 1 |
